# Functional Characterization of *HGD* Gene Variants by Minigene Splicing Assay

**DOI:** 10.3390/ijms262110639

**Published:** 2025-10-31

**Authors:** Andrey Nekrasov, Elza Shchukina, Beatrisa Rimskaya, Ekaterina Zakharova

**Affiliations:** 1 Research Centre for Medical Genetics, 115522 Moscow, Russia; captainlabman@gmail.com (A.N.);; 2 Petrovsky Russian Research Center for Surgery, 119435 Moscow, Russia

**Keywords:** alkaptonuria, *HGD*, homogentisate 1,2-dioxygenase, homogentisic acid, minigene, pseudoexon, splicing, AKU

## Abstract

The *HGD* gene encodes homogentisate 1,2-dioxygenase. A deficiency of this enzyme causes alkaptonuria (AKU; OMIM 203500), a monogenic autosomal recessive metabolic disorder. The global incidence of alkaptonuria is estimated at 1 in 250,000 to 1,000,000 live births. A large number of pathogenic nucleotide variants disrupt pre-mRNA splicing, leading to hereditary diseases. Many potentially splice-disruptive variants, including those in coding regions, remain uncharacterized. This lack of data makes clinical interpretation more difficult and can complicate diagnosis. We systematically analyzed 27 *HGD* variants predicted to affect splicing. Candidate variants from public databases (ClinVar, HGDdatabase) and our patient cohort were prioritized using in silico splicing predictions and evaluated with a minigene splicing assay in HEK293T cells. Based on the obtained functional analysis data, the variants were reclassified according to ACMG/AMP guidelines. In total, 13 variants changed their classification (9 were upgraded and 4 were downgraded), while 5 variants retained their pathogenicity class after analysis. Ten missense/nonsense variants were not reclassified, as no significant splicing disruption was detected. These findings improve the pathogenicity assessment of *HGD* variants, support more accurate diagnosis, and lay the foundation for future therapeutic strategies targeting splicing defects in AKU.

## 1. Introduction

Alkaptonuria (AKU; OMIM 203500) is an autosomal-recessive monogenic metabolic disorder caused by pathogenic variants in the *HGD* gene, resulting in a deficiency of homogentisate 1,2-dioxygenase [1]. Under normal conditions, this enzyme catalyzes a step in the catabolism of phenylalanine and tyrosine; its loss leads to the accumulation of homogentisic acid in all tissues, with a preferential deposition in connective tissue. Homogentisic acid is then oxidized to benzoquinone acetic acid (BQA), a black pigment. BQA polymerizes into an ochronotic, melanin-like pigment that accumulates in connective structures [1,2]. The consequences of pigment deposition most frequently include ochronosis of the eyes, ears, large-joint cartilages, and heart valves, as well as formation of stones in the kidneys, prostate, and gallbladder—often necessitating surgical intervention [3]. Recent studies have also demonstrated an association between ochronotic pigment and AA amyloid deposition in cartilage, indicating secondary amyloidosis [4].

The global incidence of alkaptonuria is estimated at 1 in 250,000 to 1,000,000 live births [5]. Cases of alkaptonuria have been reported in 40 countries. The Dominican Republic, India, Jordan, and Slovakia are among the countries with the highest number of reported cases, with a prevalence of 1 in 19,000 individuals in Slovakia [6].

The most common variants leading to the development of alkaptonuria are missense variants (64.2%), splice-affecting variants (16.1%), frameshift variants (10.6%), nonsense variants (4.6%), large deletions (4.1%), and a single stop-loss variant (0.5%) [6]. Although splice-affecting variants in *HGD* account for 16.1% and rank second in frequency, the true prevalence of splice disruption is likely underestimated: incorporating RNA-seq into diagnostic workflows increases diagnostic yield by ~8–36% across diverse rare-disease cohorts by revealing aberrant splicing missed by DNA-only testing [7]. This underrepresentation likely arises from limited intronic coverage in exome sequencing and from the difficulty of functionally and clinically interpreting intronic variants even with whole-genome sequencing. Another reason is the misinterpretation of the mechanism of missense, nonsense, and synonymous variants, which in fact may act as splice-disrupting variants [8]. This significantly complicates the clinical interpretation of genetic variants in suspected alkaptonuria, making it more difficult to establish an accurate diagnosis. At the same time, clarifying the exact pathogenic mechanisms of such variants may contribute to the development and application of innovative personalized therapies.

Pre-mRNA splicing is an essential step in transcript processing, carried out by the spliceosome—a complex of small nuclear ribonucleoproteins and auxiliary splicing factors that recognize *cis*-elements on the nascent transcript, including the 5′ and 3′ splice sites, the branchpoint, and the polypyrimidine tract [9]. Variants within these elements may be splice-disrupting, leading to the production of aberrant transcripts and disease [10].

In the absence of functional RNA studies, many predicted splice-disrupting variants—excluding canonical ±1/±2 dinucleotide variants—are classified as variants of uncertain significance (VUS) and may be misannotated in terms of their pathogenic mechanism (for example, missense, nonsense, or synonymous variants that actually disrupt splicing). Therefore, RNA analysis is critical for accurate clinical classification. However, patient-derived RNA is not always available, and endogenous gene expression may be too low or masked by the wild-type allele [11]. As an alternative, a minigene splicing assay can functionally characterize putative splice-disrupting variants [12].

To date, eleven *HGD* variants have been functionally characterized by various research groups, including two synonymous variants—c.753C>T (p.Gly251=) in exon 10, which activates a cryptic donor site resulting in a 23-nt deletion and frameshift [13], and c.15G>A (p.Lys5=) at the intron 1 donor site, causing exon 1 skipping and use of an alternative start codon [14]—the deep-intronic variant c.16-2063A>C, which induces pseudoexon inclusion between exons 1 and 2 [15], and five variants (in exons 9 and 10: c.649+39T>G, c.650-13T>G, c.650-17G>A, c.650-56T>G) that disrupt splicing and reduce the level of the normal isoform to 1–73%, one (c.650-85A>G) that increases it to 92%, and three (c.343-11G>A, c.372C>T, c.1191A>C) that show no significant effect on splicing [16].

The aim of this study is to analyze the splicing effects of HGD variants that are predicted to affect splicing but for which no experimental data have previously been available. Variants are sourced from ClinVar, the HGDdatabase, and a patient cohort with confirmed alkaptonuria at the Research Center for Medical Genetics, Moscow, Russia. Splicing effects are tested with a minigene assay, and most variants are reclassified under ACMG/AMP guidelines [12,17]. This work improves the accuracy of variant classification for alkaptonuria diagnosis, clarifies the pathogenic mechanisms of splicing-disrupting *HGD* variants, and lays the groundwork for future therapeutic strategies targeting splicing defects.

## 2. Results

### 2.1. Assessment of the Spliceogenic Potential of HGD Gene Variants

To evaluate the spliceogenic potential of variants in the *HGD* gene, a comprehensive in silico analysis was performed using the splicing prediction algorithm SpliceAI. All possible single-nucleotide variant (SNV) substitutions at each genomic position were analyzed, yielding a total of 162,291 unique SNVs assessed for their potential impact on splicing.

**Figure 1:**
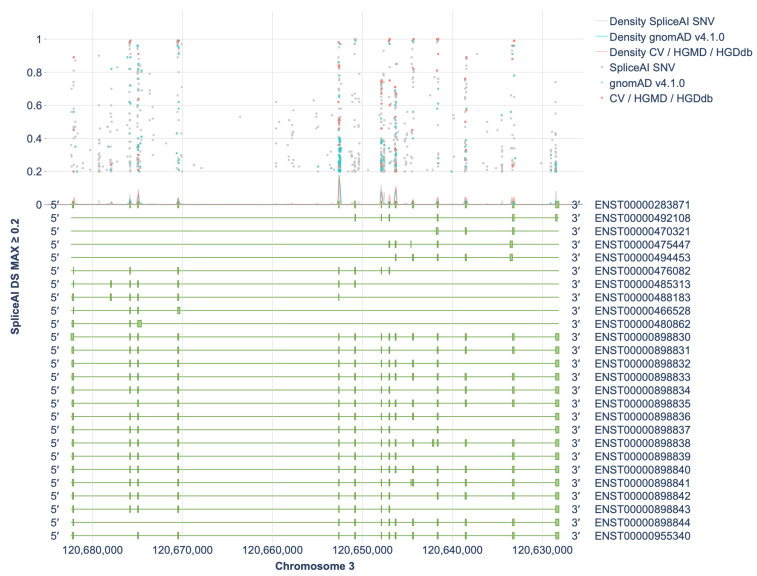
Distribution of SpliceAI DS MAX Scores ≥ 0.2 Across Transcripts of the *HGD* Gene. CV—ClinVar, HGDdb—HGDdatabase.

Of these, 1098 variants (0.67%) were predicted to have a SpliceAI DS MAX (Delta Score Maximum) score ≥ 0.2, indicating a potential to affect splicing (Figure 1). Figures for each exon individually (together with 500 bp of flanking intronic sequences) are provided in Supplementary S6. Among them, 655 variants (59.6%) had scores ranging from 0.2 to 0.49, 238 variants (21.6%) had scores between 0.5 and 0.79, and 205 variants (18.6%) scored between 0.8 and 1.0. As an external performance reference, SpliceAI achieved 94.5% sensitivity and 94.3% specificity at an optimal Δ-score > 0.22 (AUC = 0.975) in an *NF1* cohort with cDNA-validated outcomes (285 variants) [8]. The *NF1* benchmark at Δ ≈ 0.22 aligns with our ≥0.2 entry threshold, supporting its use as a sensitive screen, while higher Δ-bins are more likely to harbor true spliceogenic events.

**Figure 2:**
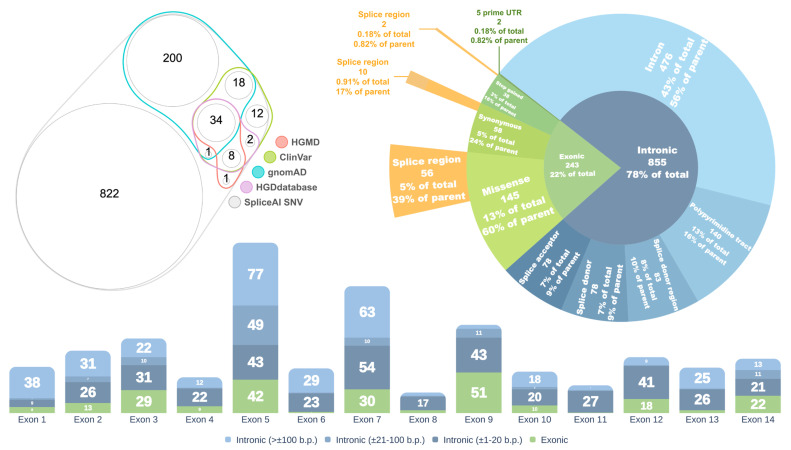
**Left**—Venn diagram showing the distribution of *HGD* gene variants with SpliceAI DS MAX ≥ 0.2 across different databases. **Right**—Distribution of variants by consequence. **Bottom**—Distribution of variants relative to exons of the canonical *HGD* transcript. Values in parentheses indicate the distance from the nearest exon.

Out of the 1098 SNVs with a DS MAX ≥ 0.2, 253 (23%) are reported in the gnomAD database, 75 (6.8%) are annotated in ClinVar, 43 (3.9%) in HGMD [18], and 45 (4.1%) in the HGDdatabase (Figure 2, left panel).

The majority of spliceogenic variants (855, or 78%) are located within intronic regions, while 243 (22%) reside in exons. Among the intronic variants, 476 (56%) are classified as deep intronic, located outside canonical splice site elements. The remaining 44% are situated within defined splice site regions (Figure 2, right panel).

Exonic variants are predominantly missense (145 variants, 60%), followed by synonymous (58 variants, 24%), stop-gain (38 variants, 16%), and two variants (0.82%) located in the 5′ untranslated region (5′ UTR). A significant proportion of exonic variants are situated within splice regions, comprising 56 (39%) of missense, 10 (17%) of synonymous, and 2 (5%) of stop-gain variants.

The highest concentration of spliceogenic variants was observed in or near exons 5, 7, and 9 of the *HGD* gene (intronic variants were assigned based on proximity to the nearest exon) (Figure 2, bottom panel).

Thus, the proportion of variants with the potential to affect splicing is estimated at 0.67% of all possible single-nucleotide variants (SNVs), 23% of which are observed in population databases. Naturally, not all of these in silico–predicted variants will actually disrupt splicing or cause disease, but these data provide a robust in silico assessment of the spliceogenic potential of *HGD* variants and allow us to approximate their number, genomic distribution, and spectrum. Notably, many potentially splice-disruptive variants lie outside the canonical splice sites—that is, they are deep intronic—underscoring the importance of exploring these regions for novel variants and of conducting functional assays to validate their splicing-disruptive effects.

### 2.2. Selection of Variants for Functional Validation Using a Minigene Assay

**Figure 3:**
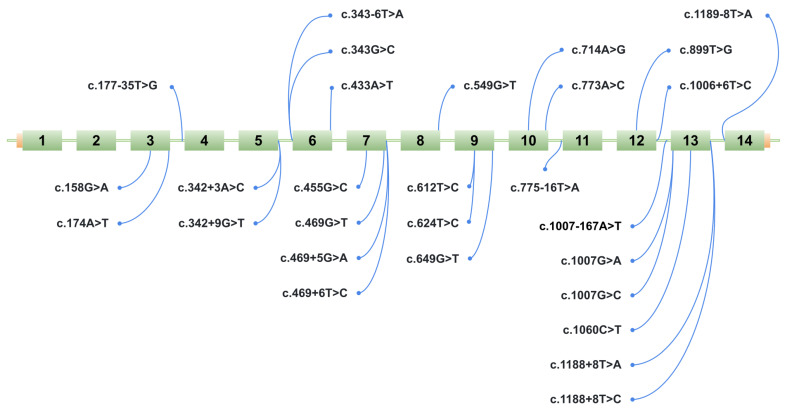
Schematic representation of the location of 28 potentially splice-disruptive variants in the *HGD* gene. The figure shows a schematic of the ManeSelect transcript of the *HGD* gene—NM_000187.4/ENST00000283871.10—including all 14 exons (numbered 1 to 14), along with the positions of the studied variants relative to the transcript.

To identify variants likely to disrupt splicing, a group of variants was selected for further experimental validation using a minigene assay. Based on the selection criteria described in the Materials and Methods Section, 28 potentially splice-disruptive variants were prioritized from a total of 394 unique variants identified in ClinVar, HGDdatabase, and our internal patient cohort, for downstream analysis via minigene assay (Figure 3, Table 1). A total of 28 variants were analyzed; however, the construct for variant c.549G>T (p.Gln183His) contained a cloning artifact, and therefore this variant was excluded from the main results. Its data are provided in Supplementary S7, and a detailed description is available in the Discussion Section.

### 2.3. Minigene Analysis

**Figure 4:**
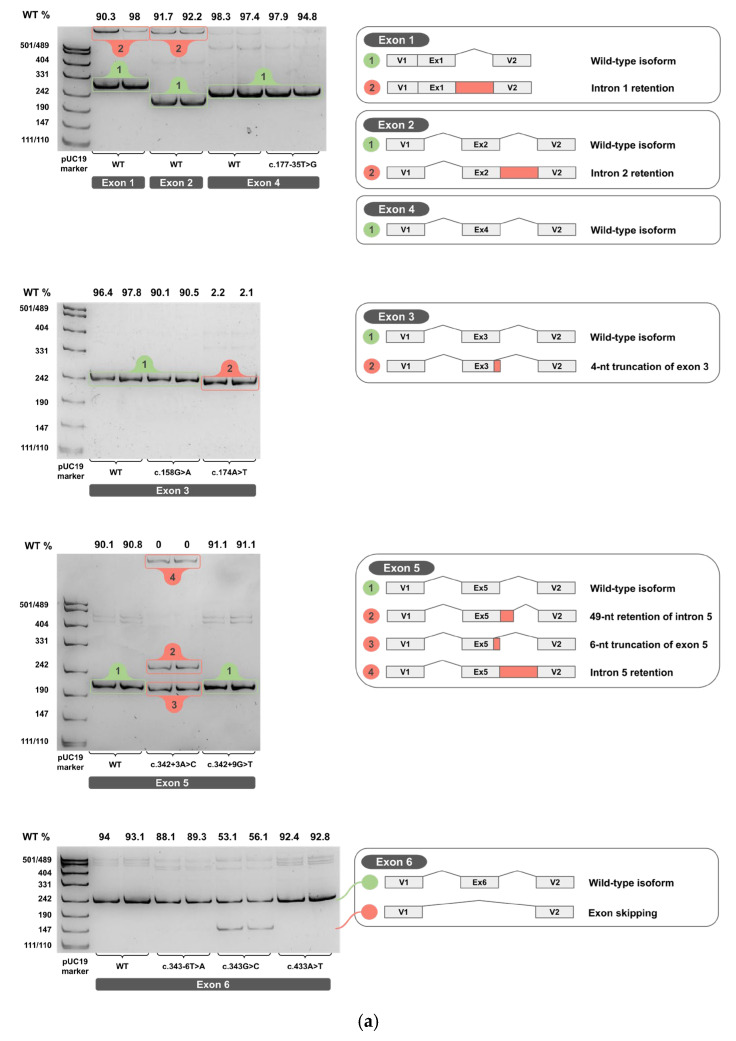

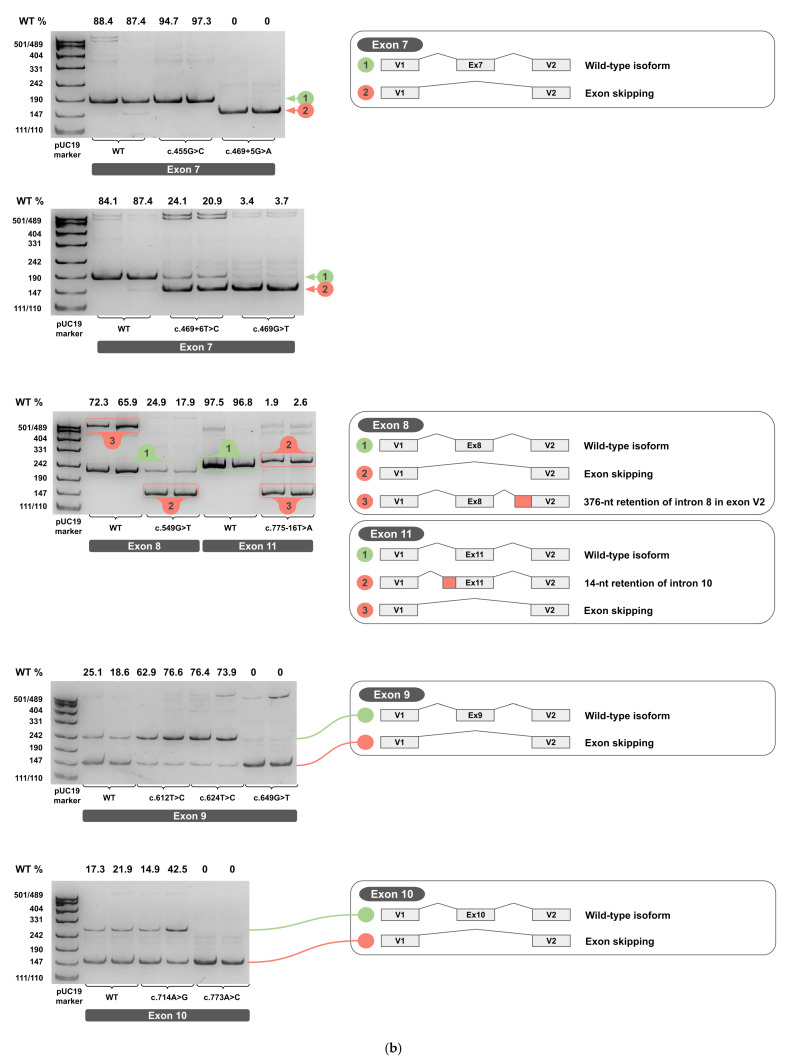

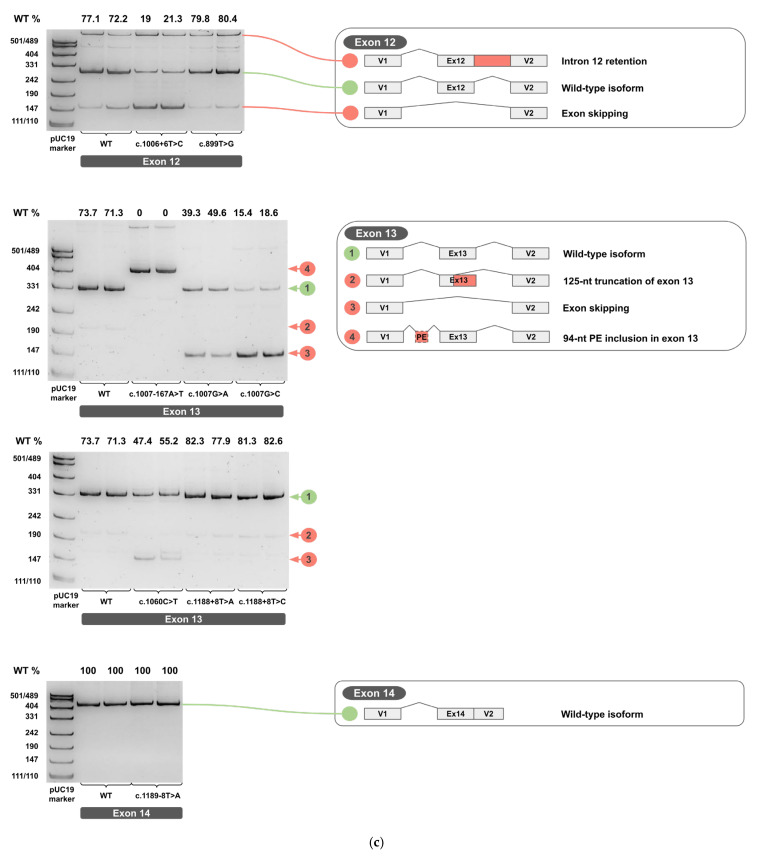
cDNA PCR products from two biological replicates visualized on 4.5% PAGE, with schematic diagrams of transcript isoforms. Green indicates wild-type isoforms, red indicates aberrant isoforms. The percentage of the wild-type isoform is shown above each band. (**a**) Exons 1-6. (**b**) Exons 7-11. (**c**) Exons 12-14.

Each minigene construct was tested in two independent biological replicates. The total RNA was extracted, reverse-transcribed into cDNA, and each cDNA replicate was PCR-amplified. Amplicons from cDNA of wild-type and variant minigenes were resolved on 4.5% PAGE and are shown in Figure 4a–c for two biological replicates (original, uncropped, and unadjusted electrophoretic gel images are provided in Supplementary S3).

The PCR products of the predominant cDNA isoforms, clearly visible on PAGE, were sequenced by Sanger sequencing to determine their exact splicing junctions. (Sanger sequencing chromatograms are provided in Supplementary S4).

**Figure 5:**
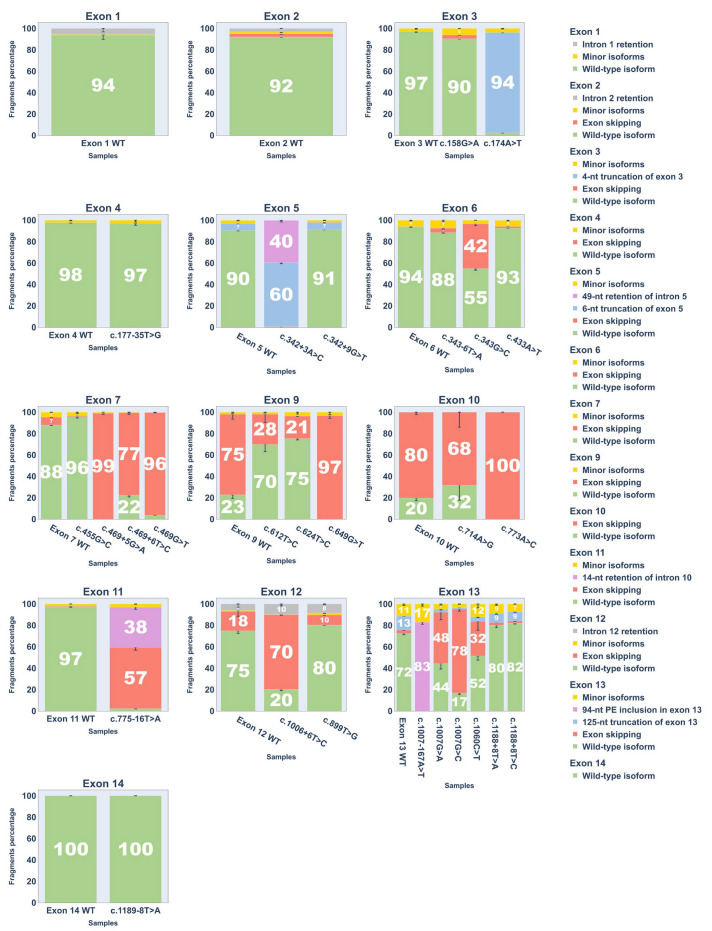
Bar plots showing Percent Spliced In (PSI) values of transcript isoforms from fragment analysis of cDNA PCR products. Error bars represent standard deviation.

To quantify splicing outcomes, each cDNA replicate was PCR-amplified with fluorescent primers for fragment analysis. Results are shown in Figure 5 and Supplementary S7 (fragment analysis chromatograms are provided in Supplementary S2).

### 2.4. A Novel Deep Intronic Variant

A deep-intronic variant, c.1007-167A>T, was identified at the Research Center for Medical Genetics (Moscow, Russia) by whole-genome sequencing in a patient under one year of age presenting with classical AKU features. The first symptom, dark-stained urine, was observed on the eighth day after birth. A markedly elevated urinary homogentisic acid level (2929.39 mM/mol creatinine; reference 0–10 mM/mol creatinine) was first detected at eight months of age.

This variant was found in compound heterozygosity with a previously reported Sanger-validated pathogenic variant, c.752G>A (p.Gly251Asp) (ACMG/AMP classification: Likely Pathogenic—PM1, PP2, PP3, PP4, PP5, BS2). Segregation analysis confirmed that the c.752G>A variant was inherited from the father, while the c.1007-167A>T variant was inherited from the mother. The c.1007-167A>T variant has not been reported in the literature or public databases and is absent from gnomAD.

**Figure 6:**
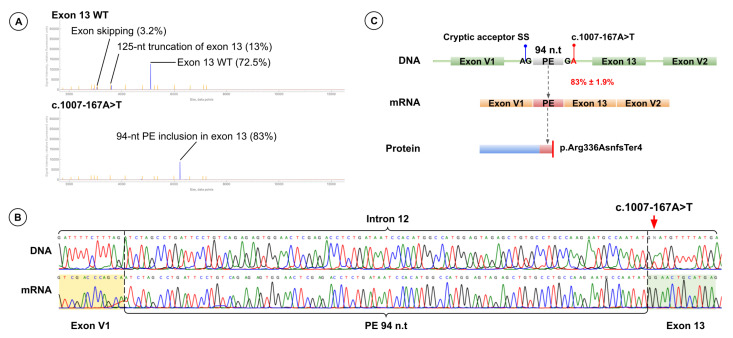
Functional Analysis Results for Variant c.1007-167A>T. (**A**) Fragment analysis of cDNA from minigenes harboring the wild-type exon 13 sequence (top) and the exon 13 sequence with c.1007-167A>T (bottom). (**B**) Sanger sequencing results of patient genomic DNA (top) harboring c.1007-167A>T (red arrow) and of cDNA from the minigene harboring the exon 13 sequence with c.1007-167A>T (bottom). (**C**) Schematic representation of the splicing effect induced by c.1007-167A>T.

Minigene splicing assay revealed that c.1007-167A>T creates a novel donor site at c.1007-168 and activates a cryptic acceptor site at c.1007-263, resulting in inclusion of a 94-nt pseudoexon. Pseudoexon insertion shifts the reading frame and introduces a premature stop codon (p.Arg336AsnfsTer4). Fragment analysis showed that 83.0% ± 1.9% of transcripts include the pseudoexon, and the full-length wild-type isoform was not detected (Figure 6).

This is the second *HGD* variant reported to induce pseudoexon inclusion. The first, c.16-2063A>C in intron 1, activates a 126-nt pseudoexon between exons 1 and 2 and was described by Lai et al. in 2021 [11].

### 2.5. ACMG/AMP Reclassification Results

**Figure 7:**
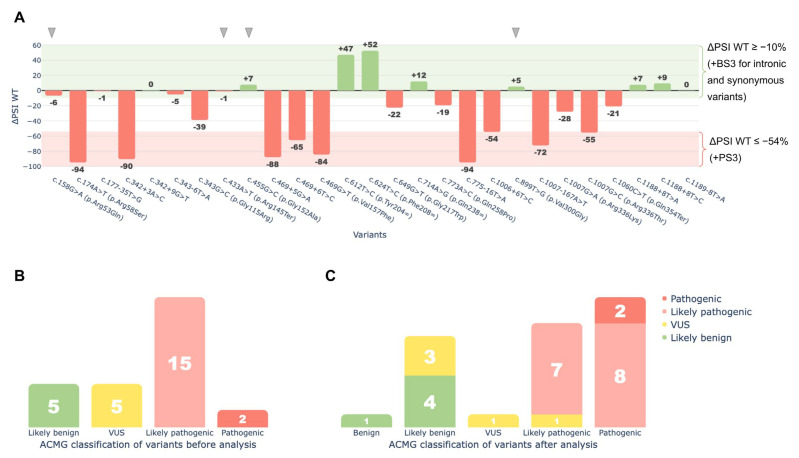
Functional analysis of 27 *HGD* variants using the minigene splicing assay. (**A**)—ΔPSI WT values showing changes in the wild-type transcript isoform for each variant. The red area (ΔPSI WT ≤ 54%) marks the cutoff where all variants were assigned the PS3 criterion. The green area (ΔPSI WT ≥ −10%) marks the cutoff where intronic and synonymous variants were assigned the BS3 criterion. For missense and nonsense variants, BS3 was not applied, even though they showed no significant effect on splicing (gray arrows). These variants may cause pathogenic effects unrelated to splicing. (**B**)—ACMG classification of variants before the minigene splicing assay. (**C**)—ACMG classification of variants after the minigene splicing assay. The X-axis shows the classification after analysis. Bar values and colors indicate the classification before the assay, illustrating reclassification.

Functional data obtained from the minigene splicing assays were integrated into the ACMG/AMP framework through the application of PS3 and BS3 criteria to reassess the clinical significance of the tested variants. As a result, 18 of the 27 variants were reclassified under ACMG/AMP guidelines based on the assay findings (Figure 7):

Eight variants were upgraded from Likely Pathogenic to Pathogenic: c.174A>T (p.Arg58Ser), c.342+3A>C, c.469G>T (p.Val157Phe), c.469+5G>A, c.469+6T>C, c.775-16T>A, c.1007-167A>T, and c.1007G>C (p.Arg336Thr).

One variant moved from VUS to Likely Pathogenic: c.1006+6T>C.

Three variants moved from VUS to Likely Benign: c.177-35T>G, c.343-6T>A, and c.1189-8T>A.

One variant was upgraded from Likely Benign to Benign: c.624T>C (p.Phe208=).

Five variants retained their original classification (four Likely Benign, and one VUS).

Nine missense or nonsense variants were not reclassified due to insufficient evidence of splice disruption under the criteria outlined in the Materials and Methods section, as their ΔPSI WT values were greater than −54%, which was the threshold established for variant reclassification.

Detailed classification results, including applied ACMG/AMP criteria, prior database classifications (ClinVar, HGDdatabase), and gnomAD allele frequencies, are provided in Table 2.

## 3. Discussion

Next-generation sequencing reveals a growing number of genetic variants. Bioinformatic predictions and functional studies are essential for their correct classification. In this study, we analyzed potentially splice-disruptive *HGD* variants reported in ClinVar, HGDdatabase, and in patients with confirmed alkaptonuria at the Research Center for Medical Genetics, Moscow, Russia. We tested 27 variants without prior experimental data using the pSPL3 minigene system in HEK293T cells. Functional studies provide important information for their clinical interpretation.

Our results confirmed or rejected the predicted splicing effects of 27 *HGD* variants. We showed that, besides intronic variants, exonic variants (synonymous, missense, and nonsense) can also disrupt splicing. Without functional testing, these variants may be misclassified or their pathogenic mechanisms misunderstood. Our findings improve the accuracy of clinical interpretation under ACMG/AMP guidelines, which is critical for diagnosing hereditary diseases.

This study also highlights the broader value of minigene splicing assays for analyzing splice-disruptive variants, particularly when patient samples are not available. Along with variants described in public databases, we identified and functionally characterized a novel deep intronic variant, c.1007-167A>T, which creates a pseudoexon in the *HGD* gene.

Finally, we developed a system of expression vector constructs covering all *HGD* exons (except exon 8), enabling the analysis of a wide range of variants. These results may support the development of personalized therapies aimed at correcting splicing defects.

### 3.1. Dual Effects of Exonic Variants

Variants in coding regions can generate two main transcript isoforms: an aberrant isoform from exon skipping or truncation, and a full-length isoform that carries the variant and may act as missense or nonsense [19].

Our assays identified three such exon-disrupting variants: two missense—c.343G>C (p.Gly115Arg, exon 6) and c.1007G>A (p.Arg336Lys, exon 13)—and one nonsense—c.1060C>T (p.Gln354Ter, exon 13). All produced both the aberrant skipped isoform and the correctly spliced full-length isoform, each >32% of total transcripts. Thus, these variants show dual pathogenicity: protein-coding effects (missense/nonsense) and exon skipping with premature termination.

For the missense variants, it is unclear which mechanism dominates. Exon skipping alone may generate enough aberrant transcripts to cause AKU, but an independent pathogenic role of the amino acid substitution cannot be excluded. AlphaMissense scores for c.343G>C and c.1007G>A are 0.893 and 0.7858, respectively (likely pathogenic).

For the nonsense variant c.1060C>T, both isoforms introduce premature stops, leaving no doubt about pathogenicity. Its effect on splicing in exon 13 may involve creation of an exonic splicing silencer, as predicted by HExoSplice (Supplementary S5).

Other missense/nonsense variants affecting exon splicing may also act by dual mechanisms, but often the full-length isoform is much less abundant than the aberrant one, suggesting a dominant splice-disruption effect.

### 3.2. Regulation of Exon 9 Splicing

Fragment analysis of wild-type minigene carrying exon 9 of the *HGD* gene showed low baseline inclusion of the full-length isoform (22.7% ± 3.3%), with most transcripts lacking exon 9 (75.3% ± 4.4%) in HEK293T cells. Introducing the synonymous variants c.612T>C (p.Tyr204=) and c.624T>C (p.Phe208=), both located in the middle of exon 9, markedly increased full-length inclusion to 69.9% ± 6.9% and 75.3% ± 1.3%, respectively, and reduced exon 9 skipping to 28.2% ± 7.6% and 21.0% ± 0.5%.

In silico analysis with HExoSplice predicted that these variants disrupt exonic splicing silencers (ESSs) and instead promote the formation of exonic splicing enhancers (ESEs), improving spliceosome recognition of exon 9.

Additionally, SpliceVault data from over 300,000 human transcriptomes show that exon 9 skipping occurs in 36.3% of samples (‘Max Reads (GTEx)’ metric: annotated splicing = 4595 reads, exon skipping = 3215 reads, skipping/annotated splicing ratio = 69%), while joint skipping of exons 9 and 10 occurs in 92% of samples (exon skipping = 340 reads, skipping/annotated splicing ratio = 7.3%), indicating that these exons are naturally prone to mis-splicing in vivo (tissue—Liver) [20]. Thus, we can observe a concordance between our minigene splicing assay results and the SpliceVault data, at least for approximately 36.6% of samples. This suggests that the low recognition of exon 9 (and exon 10, which shows a similar pattern) observed in our analysis likely reflects the natural splicing processes occurring in vivo. Additionally, we cannot currently explain why exon 9 skipping events are observed in only 36.6% of SpliceVault samples, and elucidating this phenomenon will require further investigation in future studies.

We interpret the synonymous variants c.624T>C and c.612T>C in exon 9 as benign; however, it should be noted that they may in fact be beneficial.

### 3.3. Aberrant 3′-End Deletion of Exon 13

An unexpected 125-nt 3′-end deletion isoform of exon 13 was found in both wild-type and variant minigene samples. This isoform is also seen at 13.5% frequency in SpliceVault in vivo samples (‘Max Reads (GTEx)’ metric: annotated splicing = 421 reads, 125 nt truncation = 249 reads, truncated/annotated splicing ratio = 59%) (tissue—Liver). Thus, we can see that this event also occurs in vivo, and our exon 13 expression results reasonably reflect the processes observed under physiological conditions.

It should be noted that the ‘Max Reads (GTEx)’ metric may reflect the number of reads corresponding to different events across various samples; therefore, a direct comparison of isoforms based on this metric may not fully represent their relative proportions within each individual sample.

### 3.4. Cloning Artifact Revealed by Exon 8 Analysis

During fragment analysis of wild-type exon 8 minigenes, an unexpected 607-nt band was observed. Sanger sequencing showed this isoform represents full-length exon 8 plus a 376-nt retention of the plasmid intron upstream of TagGFP2 (V2). This was caused by the creation of a strong cryptic acceptor site at the EcoRV restriction junction: the reverse primer (5′→3′ CCACTTGTCATTGCACTTTCCTA) fused with the EcoRV site (5′→3′ GATATC) forms an AG dinucleotide that is recognized by the spliceosome. This artifact, although unlikely, may affect the functional analysis results of the c.549G>T (p.Gln183His) variant in exon 8. For this reason, the results for this variant were excluded from the main analysis and are available in Supplementary S7.

Similar primer/EcoRV combinations flank exons 6, 9, 10, and 11, but only exon 8 showed this insertion, suggesting a stronger cryptic site in this context. All fragments observed on the PAGE gel were confirmed by Sanger sequencing, and no other cloning artifacts were detected.

### 3.5. Aberrant Splicing Isoforms of Exon 5

Minigene assays of c.342+3A>C revealed three major aberrant isoforms—6-nt exon 5 truncation, 49-nt exon 5 retention, and full intron 5 retention—plus minor exon skipping. The intron-retained isoform was too large for fragment analysis and could not be quantified. However, this did not affect pathogenicity assessment because the complete loss of the full-length exon 5 isoform was clear.

### 3.6. Limitations of the Minigene Splicing Assay

Although the minigene splicing assay is a well-established method for analyzing variants that may disrupt splicing, it still has certain limitations that should be acknowledged. The first limitation is related to the tissue-specific nature of gene expression. Genome-wide analyses have shown that 10–30% of alternatively spliced human genes exhibit tissue-specific splicing patterns, determined by the presence or absence of different splicing regulatory proteins (splicing factors), such as ESE (exonic splicing enhancers), ESS (exonic splicing silencers), ISE (intronic splicing enhancers), and ISS (intronic splicing silencers) in distinct tissues [21]. Thus, although HEK293T cells are frequently used for splicing analysis, they may not accurately reproduce splicing events occurring in the liver, the main site of *HGD* expression. According to the GTEx database, the liver is the primary site of HGD expression, showing the highest transcript levels (median TPM: 216.5). The kidneys also exhibit notable expression, though to a considerably lower extent (median TPM: kidney cortex—52.95, kidney medulla—29.47). For comparison, skeletal muscle demonstrates the lowest *HGD* expression among the analyzed tissues (median TPM: 0.06). These data suggest that *HGD* expression in the kidneys may have tissue-specific characteristics compared to the liver.

Given that HEK293T cells are derived from human embryonic kidney and are widely used in splicing research, they represent a reasonable compromise model for assessing HGD splicing. One of the main advantages of HEK293T cells is their high transfection efficiency and robust transcriptional and translational machinery, which allows consistent expression of exogenous constructs. Moreover, HEK293T cells maintain expression of a broad spectrum of RNA-binding proteins and splicing regulators—including many tissue-specific factors—making them a flexible and permissive system for studying diverse splicing events.

However, differences in the expression profiles of splicing regulatory factors between hepatic and kidney-derived cells may affect exon recognition efficiency and the relative abundance of alternative isoforms. Therefore, while HEK293T cells provide a practical and reproducible platform for functional splicing assays, potential tissue-specific variations in splicing regulation should be taken into account when interpreting the results.

The second limitation is the restricted genomic context of the minigene construct, which allows only relatively small gene segments to be included in the analysis. As a result, some regulatory elements essential for proper splicing may be absent. It should also be noted that minigene assays can sometimes produce a number of artifact isoforms. Nevertheless, the minigene splicing assay shows almost 100% concordance with RNA analysis from patients when assessing aberrant splicing, making it an extremely valuable tool for the study of variants with potential splicing effects, especially in cases where patient-derived material is not available [22].

### 3.7. Classification of Variants Affecting Splicing

The results of minigene splicing analysis can be used to refine the pathogenicity classification of the variants studied here by adding ACMG PS3/BS3 criteria. However, multiple splicing events and variable residual expression of the wild-type transcript may in some cases complicate pathogenicity assessment. To determine the level of reduction in wild-type transcript expression sufficient to cause disease, we analyzed the intronic variant c.1006+6T>C. This variant was previously described in a patient with a clear clinical presentation of alkaptonuria confirmed by biochemical and gene testing [23]. Our functional analysis revealed a ~54% reduction in the wild-type transcript isoform, suggesting that this degree of reduction is sufficient to cause disease. The c.1006+6T>C variant, being intronic, cannot have other pathogenic mechanisms apart from splicing. Based on these results, we applied the PS3 criterion to all variants with comparable or greater reductions in wild-type isoform expression. Similar or related approaches for using splicing analysis to assign PS3/BS3 criteria have been described in previously published studies on the PAX6 [24], SCN5A, KCNQ1, KCNH2 [25], and BRCA2 [26,27] genes. Approaches for clinical assessment of variant effects on splicing may rely on measuring the residual amount of the wild-type isoform or on evaluating PSI differences between mutant and wild-type minigenes. In both cases, the threshold for assigning PS3 is approximately 50%. The BS3 criterion is applied if the PSI difference does not exceed 10% [24,25,26,27].

The minigene splicing assay represents a valuable approach for the functional characterization of sequence variants that may disrupt pre-mRNA splicing. Unlike purely computational predictions, this method provides direct experimental evidence of aberrant splicing events and quantifies their effect on transcript isoform balance. Within the ACMG/AMP framework, such results can be directly integrated into variant classification, particularly through application of the PS3 and BS3 criteria, thereby strengthening the pathogenicity assessment. In the case of alkaptonuria, accurate classification of *HGD* variants is of particular importance, as many remain uncharacterized or are misclassified due to the absence of functional data. By refining the interpretation of these variants, minigene splicing assays contribute to more precise genetic counseling and clinical diagnosis, allowing for improved risk stratification and better-informed management strategies for affected individuals and their families. Moreover, this approach is especially valuable in situations where patient-derived biological material is unavailable, underscoring its utility as a broadly applicable diagnostic tool in rare metabolic diseases. Finally, the insights gained from functional studies of splicing-disruptive variants may also provide a foundation for the development of personalized therapeutic strategies aimed at correcting splicing defects.

## 4. Materials and Methods

This experimental study was conducted at the Research Center for Medical Genetics, Moscow, Russia, and focused on the functional analysis of *HGD* variants using a minigene splicing assay.

### 4.1. Assessment of the Spliceogenic Potential of HGD Gene Variants

To assess the spliceogenic potential of SNVs in the *HGD* gene, SpliceAI scores were retrieved from the official dataset provided by Illumina (v1.3). For each variant, the maximum delta score (DS MAX) was used as an indicator of predicted splicing impact.

Variant consequences were determined based on Ensembl consequence annotations, obtained using the Ensembl Variant Effect Predictor (VEP) tool (web interface) https://www.ensembl.org/info/docs/tools/vep/index.html (accessed on 10 May 2025).

### 4.2. Selection of Variants for Functional Validation Using a Minigene Assay

A total of 394 candidate variants were retrieved from the ClinVar, HGDdatabase repositories, as well as from an alkaptonuria patient cohort at the Federal State Budgetary Institution “Research Center for Medical Genetics” Moscow, Russia. All candidates were evaluated with splicing-prediction algorithms—SpliceAI v1.3.1 (Max Distance = 1000) [28], MMSplice v2.2 [29], and SPiP v2.1 [30]—to identify potential splice-disrupting variants according to the following criteria: alteration of donor, acceptor, branchpoint sites, or polypyrimidine tract; creation or activation of cryptic splice sites; SpliceAI ΔDS/ΔDG/ΔAL/ΔAG ≥ 0.2; SPiP Interpretation = “Alter by SPiCE”/”Alter BP”/”Alter by MES (Poly TC)”; MMSplice Δ logit ψ < −1 or > +1; and gnomAD allele frequency < 0.01. Following this selection, only single-nucleotide variants (SNVs) were included in the subsequent minigene assay. Missense variants predicted to affect splicing were further annotated with AlphaMissense (via Ensembl Variant Effect Predictor) to assess their amino acid–level pathogenicity in cases of dual (protein + splicing) effects.

### 4.3. Construction of Minigenes

**Figure 8:**
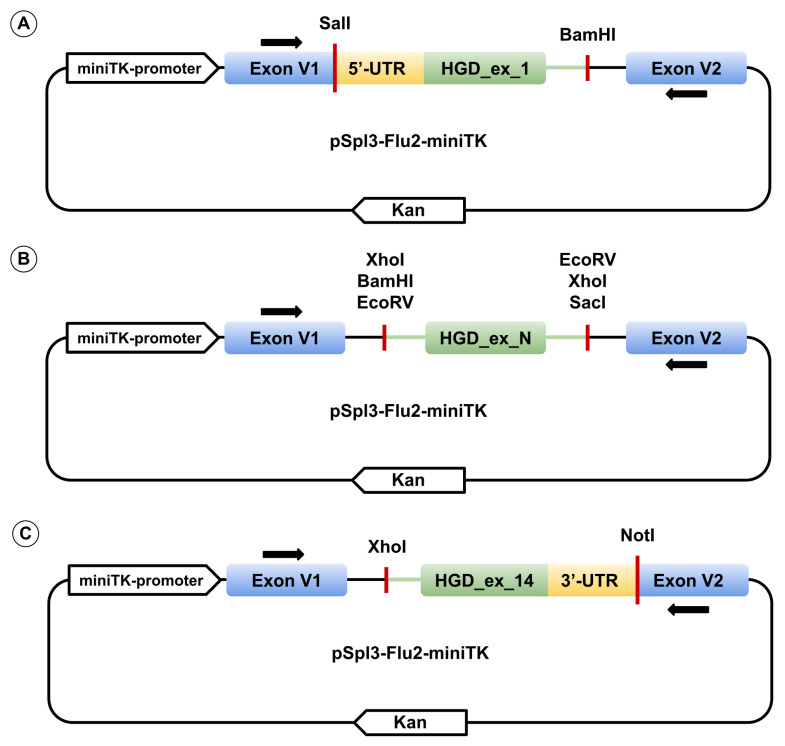
Schematic representation of the expression vector pSpl3_Flu2-miniTK containing exon 1 (**A**), exons 2–13 with HGD_ex_N representing one of the exons (**B**), and exon 14 (**C**). Black arrows above and below the exons indicate primer binding sites for cDNA amplification.

Each of the 14 wild-type *HGD* exons, together with ≥ 90 bp of flanking intronic sequence (with 5′ and 3′ UTR replacing intronic regions for the first and last exons, respectively), was PCR-amplified from human genomic DNA using HS-Fuzz DNA polymerase (Dialat, Moscow, Russia). Primers incorporated restriction sites for SalI, BamHI, XhoI, EcoRV, NotI, or SacI restriction enzymes. Amplified fragments were cloned into the multiple cloning site between two constitutively spliced exons of the pSpl3_Flu2-miniTK vector (Figure 8). pSpl3-Flu2-miniTK is a modification of pSpl3-Flu in which the CMV promoter was replaced with the HSV-TK mini promoter (–33 to +32 region of the herpes simplex virus thymidine kinase promoter) and the strong cryptic splice site downstream of the MCS was deleted [31,32]. Single-nucleotide variants were introduced via site-directed mutagenesis. All constructs were verified by Sanger sequencing using primers pSpl3_F and pSpl3-Flu2R for exons 2–13, TurboFP_F and pSpl3-Flu2R for exon 1, and pSpl3_F and EGFP_R3I for exon 14 (primer sequences for genomic amplification, mutagenesis, and plasmid sequencing are listed in Supplementary Table S1).

### 4.4. Minigene Splicing Assay

#### 4.4.1. Transfection

HEK293T cells (ATCC CRL-3216™) were used for all transfections. For constructs bearing exons 1, 2, 4, 6–12, and 14 (wild-type and variant-bearing), cells at ~80% confluence in 24-well plates were transfected with 0.5 µg plasmid DNA by the calcium-phosphate method in two biological replicates [31]. Constructs bearing exons 3, 5, and 13 (wild-type and variant) were transfected under identical conditions using 0.5 µg plasmid DNA and 1.5 µL TurboFect Transfection Reagent (Thermo Fisher Scientific, Waltham, MA, USA) in two biological replicates. After 48 h of culture, cells were harvested for RNA extraction.

#### 4.4.2. RNA Extraction, cDNA Synthesis, and Analysis

Total RNA was isolated with the ExtractRNA (Evrogen, Moscow, Russia) and treated with DNase I (Evrogen, Moscow, Russia). First-strand cDNA synthesis used 5X RT MasMIX (Dialat, Moscow, Russia) and oligo(dT) primers (Evrogen, Moscow, Russia). cDNA was PCR-amplified with HS-Fuzz DNA polymerase (Dialat, Moscow, Russia) using TurboFP_F and eGFP_R primers for exons 1–13, and TurboFP_F and EGFP_R3I primers for exon 14. Amplification products were resolved on 4.5% polyacrylamide gels (PAGE), extracted, and Sanger-sequenced on an ABI PRISM 3500xL Genetic Analyzer (Thermo Fisher Scientific, Waltham, MA, USA). Primer sequences are provided in Supplementary Table S1. Individual bands were excised from 1% agarose gels for cleanup when necessary. pUC19 served as the size marker for PAGE-resolved amplicons.

#### 4.4.3. Fragment Analysis

To quantify average inclusion levels of both wild-type and aberrant transcript isoforms across two biological replicates, cDNA from each sample was PCR-amplified using HS-Fuzz DNA polymerase (Dialat, Moscow, Russia). Plasmid-specific, fluorescence-labeled primers were used: TurboFP_F and GFP-R-FAM for exons 1–13, and TurboFP_F_FAM and EGFP_R3I for exon 14. Amplicons were analyzed by capillary electrophoresis on an ABI PRISM 3500xL Genetic Analyzer (Thermo Fisher Scientific, Waltham, MA, USA). Primer sequences are listed in Supplementary Table S1, and representative electropherograms are shown in Supplementary S2. The Applied Biosystems GeneScan 500 size standard (Thermo Fisher Scientific, Waltham, MA, USA) https://documents.thermofisher.com/TFS-Assets/LSG/manuals/cms_042491.pdf (accessed on 20 December 2023) was used [33]. Fragment data were quantified using Coffalyser.Net (v240129.1959) https://www.mrcholland.com/technology/software/coffalyser-net (accessed on 12 February 2024) and visualized with FragalyseQt (v0.3.1) https://github.com/Dorif/fragalyseqt (accessed on 12 February 2024).

The percent-spliced in (PSI) value for each isoform was calculated as the sum of peak heights for that isoform across both biological replicates divided by the sum of peak heights of all detected isoforms across both replicates, multiplied by 100:

$$PSI\;(\%)=\frac{\sum \left(\mathrm{isoform}\;\mathrm{peak}\;\mathrm{heights}\;\mathrm{in}\;\mathrm{two}\;\mathrm{replicates}\right)}{\sum \left(\mathrm{all}\;\mathrm{isoform}\;\mathrm{peak}\;\mathrm{heights}\;\mathrm{in}\;\mathrm{two}\;\mathrm{replicates}\right)}\times 100$$

Standard deviation across replicates was computed using NumPy’s std function.

Absolute and relative deltas in full-length isoform abundance were calculated for each variant minigene relative to the wild-type minigene. The absolute delta (ΔPSI) was defined as

$$\Delta \mathrm{PSI}\;\left(\%\right)=\mathrm{PS}I_{\mathrm{variant}}-\mathrm{PS}I_{\mathrm{WT}}$$

where PSI (%) is the percentage of full-length exon inclusion. The relative delta (δPSI) was calculated as

$$\delta \mathrm{PSI}\;\left(\%\right)\;=\;\frac{\mathrm{PS}I_{\mathrm{variant}}}{\mathrm{PS}I_{\mathrm{WT}}}\;\times 100$$

### 4.5. Variant Nomenclature and Accession

Variants are described according to Human Genome Variation Society (HGVS) guidelines http://www.hgvs.org/mutnomen (accessed on 20 January 2024) using the reference sequence NCBI RefSeq NM_000187.4 (Ensembl transcript ID ENST00000283871.10) and human genome version GRCh38 (HG38). Predicted effects of variants at the protein level were obtained using the Ensembl Variant Effect Predictor (VEP) web interface [34,35].

### 4.6. Clinical Classification of Variants

Clinical classification was performed in accordance with the ACMG/AMP guidelines before and after functional assays, applying PS3/BS3 criteria only following the minigene results.

PS3 was only assigned to variants with ΔPSI ≤ −54% (i.e., a ≥ 54% reduction in wild-type isoform inclusion).

BS3 was assigned to intronic or synonymous variants with ΔPSI ≥ −10% (i.e., ≤10% reduction).

BS3 was not assigned to missense or nonsense variants, even if ΔPSI ≥ −10%, because the absence of a substantial splicing defect should not downgrade a variant that may still be pathogenic at the protein level.

Reclassification of missense/nonsense variants was based solely on their splicing effect. Missense-specific ACMG criteria (PS1, PM1, PM5, PP2/BP1, and PP3/BP4 when predicting amino acid impact) were excluded during reclassification, as they assess protein-level consequences rather than splicing.

Variants were not reclassified for splice disruption if the wild-type minigene showed <70% full-length isoform inclusion and the variant caused a further decrease in inclusion. However, if the variant led to increased inclusion of the full-length isoform, this was still considered even when baseline inclusion was below 70%.

The 70% threshold was chosen based on the quantitative assessment of exon 13 isoform levels in the wild-type minigene, which demonstrates that an exon analyzed using the minigene splicing assay should not necessarily account for 100% inclusion in the wild-type isoform. This follows from the fact that even under in vivo conditions (according to SpliceVault data), different splicing products can be present in considerable amounts.

## 5. Conclusions

Our study improves the accuracy of variant classification in alkaptonuria by functionally validating 27 *HGD* gene variants through a minigene splicing assay. These results provide evidence for the pathogenic mechanisms of splicing-disrupting variants in the *HGD* gene, as well as for the absence of pathogenic effects, and highlight the value of functional assays in genetic diagnostics. By refining variant interpretation, our findings support more precise diagnosis of alkaptonuria and lay the groundwork for future therapeutic strategies targeting splicing defects in this disease.

## Figures and Tables

**Table 1:** Results of in silico analysis of 27 potentially splice-disruptive variants in the *HGD* gene. SpliceAI: DS_DL—delta score donor loss (disruption of donor site), DS_DG—delta score donor gain (creation of donor site), DS_AL—delta score acceptor loss (disruption of acceptor site), DS_AG—delta score acceptor gain (creation of acceptor site), (≥0.2—high recall, ≥0.5—recommended threshold, ≥0.8—high precision cutoff). **SPiP:** InterConfident—probability that the variant affects splicing, based on a collection of in vitro RNA-assayed and frequent variants. NTR—nothing to report. BP—branch point. ESR—exonic splicing regulator. **MMSplice:** pathogenicity—predicted pathogenic potential of the variant, efficiency—predicted impact on exon splicing efficiency. **AlphaMissense score:** 0–0.34—likely benign, 0.34–0.564—uncertain, 0.564–0.78—likely pathogenic (moderate), 0.78–1.0—likely pathogenic (strong). **gnomAD v4.1.0 total** includes combined allele frequency data from both exome and genome sequencing datasets. Cell coloring reflects prediction scores, with shades of green indicating lower impact on splicing and shades of red indicating higher predicted pathogenicity.

Variant	Exon	gnomAD v4.1.0 Total	SpliceAI				SPiP			MMSplice	AlphaMissense
			DS_AG	DS_AL	DS_DG	DS_DL	Interpretation	BP	InterConfident	Delta Logit Psi	Score
c.158G>A (p.Arg53Gln)	3	0.00003107	0.01	0.18	0	0.27	Alter by create New splice site + Alter ESR	No	85.91	−0.41	0.77
c.174A>T (p.Arg58Ser)	3	0	0	0.02	0.91	0.71	Alter by SPiCE + Alter ESR	No	98.11	−2.62	0.97
c.177-35T>G	4	0	0	0.02	0	0.02	Alter BP	Yes	10.53	−0.40	-
c.342+3A>C	5	0.000003728	0	0.01	0.32	0.84	Alter by SPiCE	No	98.41	−3.85	-
c.342+9G>T	5	6.227 × 10^7^	0.01	0.01	0.08	0.23	NTR	No	0.72	0.33	-
c.343-6T>A	6	0.00002737	0.01	0.1	0	0.05	Alter by SPiCE	No	54	−1.48	-
c.343G>C (p.Gly115Arg)	6	0.00004528	0.02	0.08	0.01	0.02	Alter by SPiCE	No	69.33	−0.79	0.89
c.433A>T (p.Arg145Ter)	6	0	0.01	0.03	0.01	0.01	Alter by SPiCE	No	54	−1.10	-
c.455G>C (p.Gly152Ala)	7	0.000003101	0.23	0	0.18	0	NTR	No	8.04	1.10	0.51
c.469+5G>A	7	0	0	0.6	0	0.75	Alter by SPiCE	No	98.41	−3.82	-
c.469+6T>C	7	0	0	0.49	0	0.61	Alter by SPiCE	No	85.91	−0.97	-
c.469G>T (p.Val157Phe)	7	0.000001862	0	0.58	0	0.7	Alter by SPiCE	No	98.41	−2.48	0.87
c.549G>T (p.Gln183His)	8	0.000003100	0.01	0.42	0.11	0.75	Alter by SPiCE	No	98.41	−4.09	0.88
c.612T>C (p.Tyr204=)	9	0.000003099	0.2	0	0.21	0	Alter by complex event	No	26.62	0.34	-
c.624T>C (p.Phe208=)	9	0.000002480	0.21	0	0.25	0	NTR	No	7.62	0.51	-
c.649G>T (p.Gly217Trp)	9	0	0	0.66	0	0.67	Alter by SPiCE	No	98.41	−3.87	1.00
c.714A>G (p.Gln238=)	10	0	0	0	0.02	0.01	Alter by creating a new splice site + Alter ESR	No	47.89	−0.19	-
c.773A>C (p.Gln258Pro)	10	0.000002478	0.01	0.04	0.09	0.05	Alter by SPiCE	No	98.41	−0.45	0.88
c.775-16T>A	11	6.433 × 10^7^	0.81	0.35	0	0.05	Alter by MES (Poly TC)	No	98.41	−2.56	-
c.1006+6T>C	12	0	0	0.45	0	0.57	Alter by SPiCE	No	96.71	−1.04	-
c.899T>G (p.Val300Gly)	12	0.00004151	0.5	0.01	0.07	0	NTR	No	9.76	0.37	0.95
c.1007-167A>T	13	0	0.47	0	0.66	0.01	Alter by creating de Novo Exon	No	2.66	−0.17	-
c.1007G>A (p.Arg336Lys)	13	0	0.09	0.15	0	0.03	Alter by SPiCE	No	43.04	−0.95	0.79
c.1007G>C (p.Arg336Thr)	13	0	0.11	0.24	0	0.04	Alter by SPiCE	No	30.67	−0.79	0.98
c.1060C>T (p.Gln354Ter)	13	0	0.02	0.06	0	0.02	Alter by creating a new splice site + Alter ESR	No	26.62	−0.19	-
c.1188+8T>A	13	0.000001239	0	0.01	0.15	0	Alter by creating a new splice site	No	30.67	−0.57	-
c.1188+8T>C	13	0	0	0	0.02	0	Alter by creating a new splice site	No	13.87	−0.06	-
c.1189-8T>A	14	0	0	0.21	0	0.06	Alter by SPiCE	No	43.04	−0.70	-

**Table 2:** Variant classification results. Cell coloring reflects ΔPSI levels, with different shades of green showing weaker effects on splicing and shades of red showing stronger splice-disruptive effects. Absolute WT ΔPSI (%) represents the absolute difference in wild-type isoform levels between wild-type and mutant constructs. Calculation methods are described in the Materials and Methods Section.

Variant	Exon	Clinvar	HGDdatabase	Absolute WT ΔPSI (%)	Pathogenicity Criteria Before Analysis	Pathogenicity Criteria After Analysis	Pathogenicity Class Before Analysis	Pathogenicity Class After Analysis
c.158G>A (p.Arg53Gln)	3	Pathogenic/Likely pathogenic	Pathogenic	−6.8	PM3, PP3, PM2, PM5, PP2, PP5	-	Likely pathogenic	-
c.174A>T (p.Arg58Ser)	3	Pathogenic	Pathogenic	−94.9	PM3, PP3, PM2, PM5, PP2, PP5	PM3, PP3, PM2, PP5, PS3	Likely pathogenic	Pathogenic
c.177-35T>G	4	-	Probably no pathogenicity	−1.0	PM2, BP7	PM2, BP7, BS3	VUS	Likely benign
c.342+3A>C	5	Conflicting classifications of pathogenicity Pathogenic(1); Uncertain significance(1)	Pathogenic	−90.5	PM3, PP3, PM2, PP5	PM3, PP3, PM2, PP5, PS3	Likely pathogenic	Pathogenic
c.342+9G>T	5	Likely benign	-	+0.6	PM2, BP4, BP6	PM2, BP4, BP6, BS3	Likely benign	Likely benign
c.343-6T>A	6	Likely benign	-	−5.3	PM2, BP6	PM2, BP6, BS3	VUS	Likely benign
c.343G>C (p.Gly115Arg)	6	Likely pathogenic	Pathogenic	−39.0	PM3, PP3, PM2, PM1, PP2, PP5	-	Likely pathogenic	-
c.433A>T (p.Arg145Ter)	6	Pathogenic	Pathogenic	−1.2	PVS1, PM2, PM3, PP5	-	Pathogenic	-
c.455G>C (p.Gly152Ala)	7	Uncertain significance	Pathogenic	+7.8	PP3, PM2, PM1, PM5, PP2	-	Likely pathogenic	-
c.469+5G>A	7	Pathogenic	Pathogenic	−88.0	PP3, PM2, PM3, PP5	PP3, PM2, PM3, PP5, PS3	Likely pathogenic	Pathogenic
c.469+6T>C	7	Pathogenic	Pathogenic	−65.6	PP3, PM2, PM3, PP5	PP3, PM2, PM3, PP5, PS3	Likely pathogenic	Pathogenic
c.469G>T (p.Val157Phe)	7	Pathogenic	Pathogenic	−84.5	PP3, PM2, PP2, PM3, PP5	PP3, PM2, PM3, PP5, PS3	Likely pathogenic	Pathogenic
c.549G>T (p.Gln183His)	8	Uncertain significance	Pathogenic	−48.1	PP3, PM2, PM5, PP2	-	Likely pathogenic	-
c.612T>C (p.Tyr204=)	9	Likely benign	-	+47.2	PM2, BP7, BP6	PM2, BP7, BP6, BS3	Likely benign	Likely benign
c.624T>C (p.Phe208=)	9	Likely benign	-	+52.6	PM2, BS2, BP7, BP6	PM2, BS2, BP7, BP6, BS3	Likely benign	Benign
c.649G>T (p.Gly217Trp)	9	Pathogenic	Pathogenic	−22.7	PP3, PM2, PP2, PM3, PP5	-	Likely pathogenic	-
c.714A>G (p.Gln238=)	10	Likely benign	-	+12.1	PM2, BP7, BP6	PM2, BP7, BP6, BS3	Likely benign	Likely benign
c.773A>C (p.Gln258Pro)	10	Pathogenic	Pathogenic	−19.6	PM2, PM1, PP3, PP2, PM3, PP5	-	Likely pathogenic	-
c.775-16T>A	11	Pathogenic	Pathogenic	−94.9	PM2, PM3, PP5, PP3	PM2, PM3, PP5, PP3, PS3	Likely pathogenic	Pathogenic
c.1006+6T>C	12	Pathogenic	Pathogenic	−54.7	PP3, PM2, PP5	PP3, PM2, PP5, PS3	VUS	Likely pathogenic
c.899T>G (p.Val300Gly)	12	Pathogenic/Likely pathogenic	Pathogenic	+5.3	PM3, PP3, PM2, PM5, PP2, PP5	-	Likely pathogenic	-
c.1007-167A>T	13	-	-	−72.5	PM2, PM3, PP3, PP4	PM2, PM3, PP3, PP4, PS3	Likely pathogenic	Pathogenic
c.1007G>A (p.Arg336Lys)	13	Pathogenic	Pathogenic	−28.1	PM2, PM1, PP3, PM5, PP2, PM3, PP5	-	Likely pathogenic	-
c.1007G>C (p.Arg336Thr)	13	Pathogenic	Pathogenic	−55.6	PP3, PM2, PM1, PM5, PP2, PM3, PP5	PP3, PM2, PM3, PP5, PS3	Likely pathogenic	Pathogenic
c.1060C>T (p.Gln354Ter)	13	Pathogenic	Pathogenic	−21.0	PVS1, PM2, PM3, PP5	-	Pathogenic	-
c.1188+8T>A	13	Pathogenic	Pathogenic	+7.7	PM2, PM3, PP5	PM2, PM3, PP5, BS3	VUS	VUS
c.1188+8T>C	13	Likely benign	-	+9.5	PM2, BP4, BP6	PM2, BP4, BP6, BS3	Likely benign	Likely benign
c.1189-8T>A	14	Likely benign	-	0	PM2, BP6	PM2, BP6, BS3	VUS	Likely benign

## Data Availability

The original contributions presented in this study are included in the article/Supplementary Material. Further inquiries can be directed to the corresponding author.

## Supplementary Materials

The following supporting information can be downloaded at: https://www.mdpi.com/article/10.3390/ijms262110639/s1.

## Abbreviations

The following abbreviations are used in this manuscript:

AKU	Alkaptonuria
HGD	Homogentisate 1, 2-dioxygenase
BQA	Benzoquinone acetic acid
RNA	Ribonucleic acid
cDNA	Complementary DNA
PAGE	Polyacrylamide Gel Electrophoresis
PCR	Polymerase Chain Reaction
VUS	Variant of Uncertain Significance
ACMG/AMP	American College of Medical Genetics and Genomics/Association for Molecular Pathology
DS MAX	Delta Score Maximum
SS	Splice site
BP	Branch point
ESR	Exonic splicing regulator
PSI	Percent-Spliced-In
ΔPSI	Absolute change in Percent-Spliced-In
δPSI	Relative change in Percent-Spliced-In
SNV	Single Nucleotide Variant
UTR	Untranslated Region
MCS	Multiple Cloning Site
WT	Wild Type
PE	Pseudoexon
HGMD	Human Gene Mutation Database
VEP	Variant Effect Predictor
HExoSplice	Human Exonic Splicing Regulatory Element Predictor
CMV	Cytomegalovirus
HSV-TK	Herpes Simplex Virus Thymidine Kinase
FAM	Fluorescein Amidite (fluorescent dye label)
NCBI	National Center for Biotechnology Information
RefSeq	Reference Sequence
GRCh38	Genome Reference Consortium Human Build 38
HEK293T	Human Embryonic Kidney 293T cells
AA	Amyloid A
NTR	Nothing to Report
TPM	Transcripts Per Million
SD	Standard Deviation

## References

[1] Garrod A.E. (1996). The incidence of alkaptonuria: A study in chemical individuality. Mol. Med..

[2] Zannoni V.G., Lomtevas N., Goldfinger S. (1969). Oxidation of homogentisic acid to ochronotic pigment in connective tissue. Biochim. Biophys. Acta.

[3] Mistry J.B., Bukhari M., Taylor A.M. (2013). Alkaptonuria. Rare Dis..

[4] Millucci L., Spreafico A., Tinti L., Braconi D., Ghezzi L., Paccagnini E., Bernardini G., Amato L., Laschi M., Selvi E. (2012). Alkaptonuria is a novel human secondary amyloidogenic disease. Biochim. Biophys. Acta.

[5] Phornphutkul C., Introne W.J., Perry M.B., Bernardini I., Murphey M.D., Fitzpatrick D.L., Anderson P.D., Huizing M., Anikster Y., Gerber L.H. (2002). Natural history of alkaptonuria. N. Engl. J. Med..

[6] Bernardini G., Braconi D., Zatkova A., Sireau N., Kujawa M.J., Introne W.J., Spiga O., Geminiani M., Gallagher J.A., Ranganath L.R. (2024). Alkaptonuria. Nat. Rev. Dis. Primers.

[7] Yépez V.A., Gusic M., Kopajtich R., Mertes C., Smith N.H., Alston C.L., Ban R., Beblo S., Berutti R., Blessing H. (2022). Clinical implementation of RNA sequencing for Mendelian disease diagnostics. Genome Med..

[8] Ha C., Kim J.W., Jang J.H. (2021). Performance Evaluation of SpliceAI for the Prediction of Splicing of NF1 Variants. Genes.

[9] Love S.L., Emerson J.D., Koide K., Hoskins A.A. (2023). Pre-mRNA splicing-associated diseases and therapies. RNA Biol..

[10] Ward A.J., Cooper T.A. (2010). The pathobiology of splicing. J. Pathol..

[11] Bueno-Martínez E., Sanoguera-Miralles L., Valenzuela-Palomo A., Esteban-Sánchez A., Lorca V., Llinares-Burguet I., Allen J., García-Álvarez A., Pérez-Segura P., Durán M. (2022). Minigene-based splicing analysis and ACMG/AMP-based tentative classification of 56 ATM variants. J. Pathol..

[12] Cooper T.A. (2005). Use of minigene systems to dissect alternative splicing elements. Methods.

[13] Bychkov I., Kamenets E., Kurkina M., Rychkov G., Ilyushkina A., Filatova A., Guseva D., Baydakova G., Nekrasov A., Cheblokov A. (2021). Alkaptonuria in Russia: Mutational spectrum and novel variants. Eur. J. Med. Genet..

[14] Tao L., Deng C., Ma M., Zhang Y., Duan J., Li Y., Fang L., Zhou Y., He X., Wang Y. (2022). A novel mutation in the homogentisate 1,2 dioxygenase gene identified in Chinese Hani pediatric patients with Alkaptonuria. Clin. Chim. Acta.

[15] Lai C.Y., Tsai I.J., Chiu P.C., Ascher D.B., Chien Y.H., Huang Y.H., Lin Y.L., Hwu W.L., Lee N.C. (2021). A novel deep intronic variant strongly associates with Alkaptonuria. npj Genom. Med..

[16] Ascher D.B., Spiga O., Sekelska M., Pires D.E.V., Bernini A., Tiezzi M., Kralovicova J., Borovska I., Soltysova A., Olsson B. (2019). Homogentisate 1,2-dioxygenase (HGD) gene variants, their analysis and genotype-phenotype correlations in the largest cohort of patients with AKU. Eur. J. Hum. Genet. EJHG.

[17] Richards S., Aziz N., Bale S., Bick D., Das S., Gastier-Foster J., Grody W.W., Hegde M., Lyon E., Spector E. (2015). Standards and guidelines for the interpretation of sequence variants: A joint consensus recommendation of the American College of Medical Genetics and Genomics and the Association for Molecular Pathology. Genet. Med..

[18] Stenson P.D., Mort M., Ball E.V., Shaw K., Phillips A., Cooper D.N. (2014). The Human Gene Mutation Database: Building a comprehensive mutation repository for clinical and molecular genetics, diagnostic testing and personalized genomic medicine. Hum. Genet..

[19] Nissim-Rafinia M., Kerem B. (2002). Splicing regulation as a potential genetic modifier. Trends Genet. TIG.

[20] Dawes R., Bournazos A.M., Bryen S.J., Bommireddipalli S., Marchant R.G., Joshi H., Cooper S.T. (2023). SpliceVault predicts the precise nature of variant-associated mis-splicing. Nat. Genet..

[21] Xu Q., Modrek B., Lee C. (2002). Genome-wide detection of tissue-specific alternative splicing in the human transcriptome. Nucleic Acids Res..

[22] van der Klift H.M., Jansen A.M.L., van der Steenstraten N., Bik E.C., Tops C.M.J., Devilee P., Wijnen J.T. (2015). Splicing analysis for exonic and intronic mismatch repair gene variants associated with Lynch syndrome confirms high concordance between minigene assays and patient RNA analyses. Mol. Genet. Genom. Med..

[23] Cho S.Y., Kim J.H. (2018). Identification of HGD mutations in an alkaptonuria patient: Using the Internet to seek rare diseases. J. Genet. Med..

[24] Davydenko K., Filatova A., Skoblov M. (2025). Assessing Splicing Variants in the PAX6 Gene: A Comprehensive Minigene Approach. J. Cell. Mol. Med..

[25] O’Neill M.J., Wada Y., Hall L.D., Mitchell D.W., Glazer A.M., Roden D.M. (2022). Functional Assays Reclassify Suspected Splice-Altering Variants of Uncertain Significance in Mendelian Channelopathies. Circ. Genom. Precis. Med..

[26] Fraile-Bethencourt E., Valenzuela-Palomo A., Díez-Gómez B., Acedo A., Velasco E.A. (2018). Identification of Eight Spliceogenic Variants in BRCA2 Exon 16 by Minigene Assays. Front. Genet..

[27] Fraile-Bethencourt E., Díez-Gómez B., Velásquez-Zapata V., Acedo A., Sanz D.J., Velasco E.A. (2017). Functional classification of DNA variants by hybrid minigenes: Identification of 30 spliceogenic variants of BRCA2 exons 17 and 18. PLoS Genet..

[28] Jaganathan K., Kyriazopoulou Panagiotopoulou S., McRae J.F., Darbandi S.F., Knowles D., Li Y.I., Kosmicki J.A., Arbelaez J., Cui W., Schwartz G.B. (2019). Predicting Splicing from Primary Sequence with Deep Learning. Cell.

[29] Cheng J., Nguyen T.Y.D., Cygan K.J., Çelik M.H., Fairbrother W.G., Avsec Ž., Gagneur J. (2019). MMSplice: Modular modeling improves the predictions of genetic variant effects on splicing. Genome Biol..

[30] Leman R., Parfait B., Vidaud D., Girodon E., Pacot L., Le Gac G., Ka C., Ferec C., Fichou Y., Quesnelle C. (2022). SPiP: Splicing Prediction Pipeline, a machine learning tool for massive detection of exonic and intronic variant effects on mRNA splicing. Hum. Mutat..

[31] Bychkov I., Kuznetsova A., Baydakova G., Gorobets L., Kenis V., Dimitrieva A., Filatova A., Tabakov V., Skoblov M., Zakharova E. (2022). Processed pseudogene insertion in GLB1 causes Morquio B disease by altering intronic splicing regulatory landscape. npj Genom. Med..

[32] Filatova A.Y., Vasilyeva T.A., Marakhonov A.V., Voskresenskaya A.A., Zinchenko R.A., Skoblov M.Y. (2019). Functional reassessment of PAX6 single nucleotide variants by in vitro splicing assay. Eur. J. Hum. Genet. EJHG.

[33] (2022). GeneScan™ 500 LIZ™ Size Standard for SeqStudio™ Flex, SeqStudio™, 3500, 3730, and 3130 Series Instruments.

[34] den Dunnen J.T., Dalgleish R., Maglott D.R., Hart R.K., Greenblatt M.S., McGowan-Jordan J., Roux A.-F., Smith T., Antonarakis S.E., Taschner P.E. (2016). HGVS Recommendations for the Description of Sequence Variants: 2016 Update. Hum. Mutat..

[35] McLaren W., Gil L., Hunt S.E., Riat H.S., Ritchie G.R.S., Thormann A., Flicek P., Cunningham F. (2016). The Ensembl Variant Effect Predictor. Genome Biol..

