# Supplementary material for: Functional Characterization of HGD Gene Variants by Minigene Splicing Assay
Source: Int J Mol Sci. 2025 Oct 31;26(21):10639. doi: 10.3390/ijms262110639 (PMC12608343; doi:10.3390/ijms262110639)
Supplement: Supplementary file 1 [file ijms-26-10639-s001.zip › Supplementary/Supplementary S2 (Fragment analysis).pptx]

## Slide 1
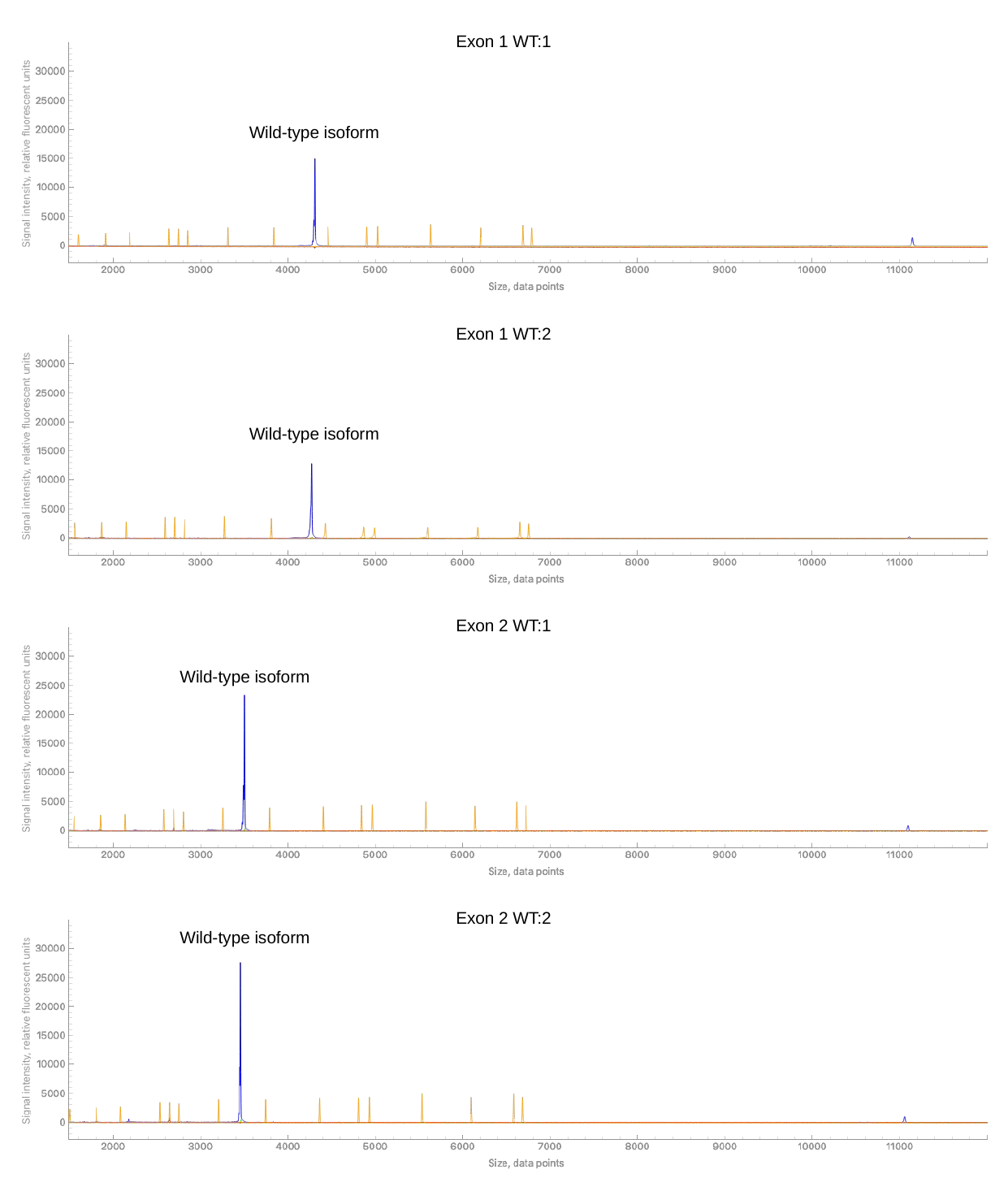

Exon 1 WT:1
Wild-type isoform
Exon 1 WT:2
Wild-type isoform
Exon 2 WT:1
Wild-type isoform
Exon 2 WT:2
Wild-type isoform

## Slide 2
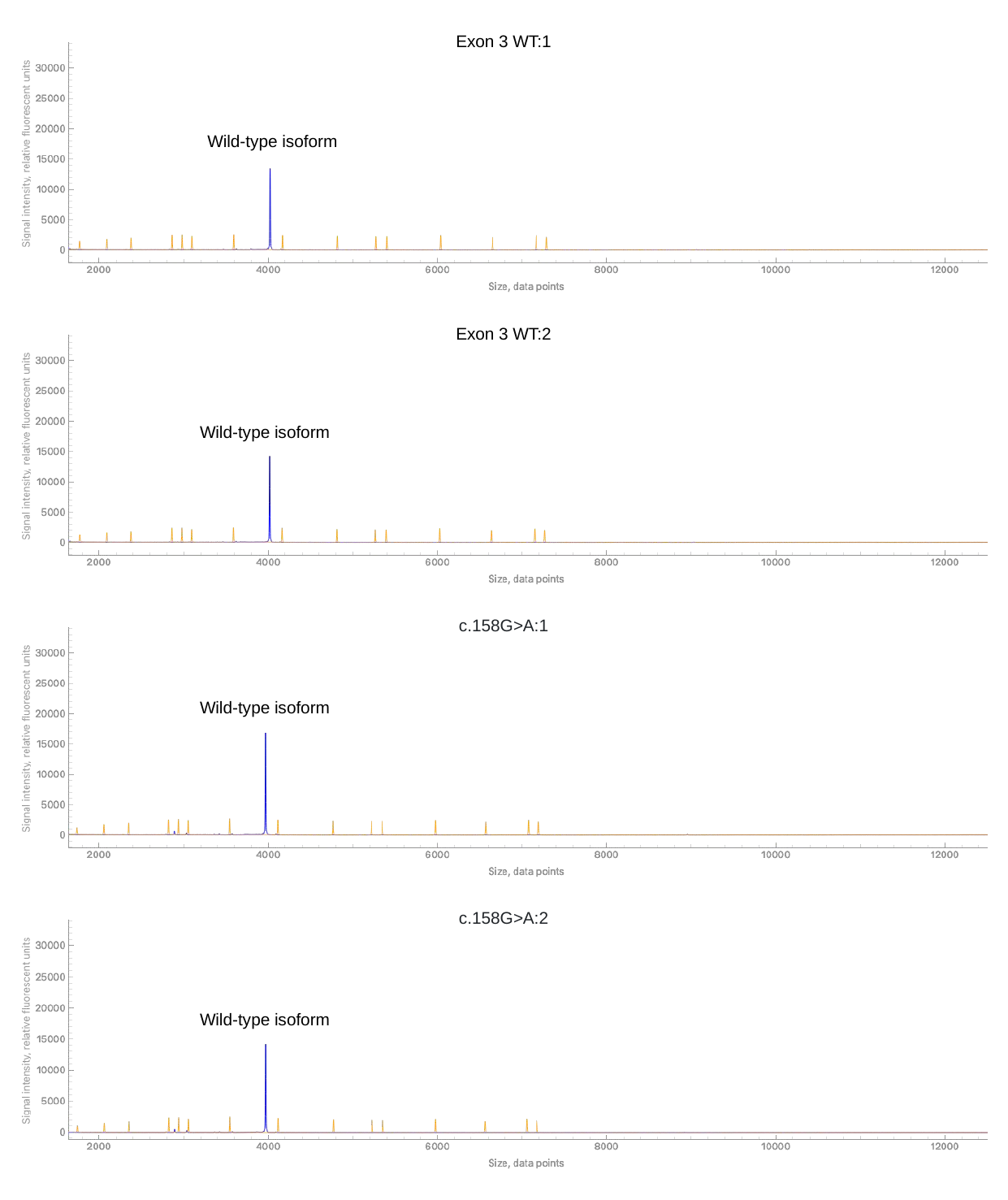

Exon 3 WT:1
Wild-type isoform
Exon 3 WT:2
Wild-type isoform
c.158G>A:1
Wild-type isoform
c.158G>A:2
Wild-type isoform

## Slide 3
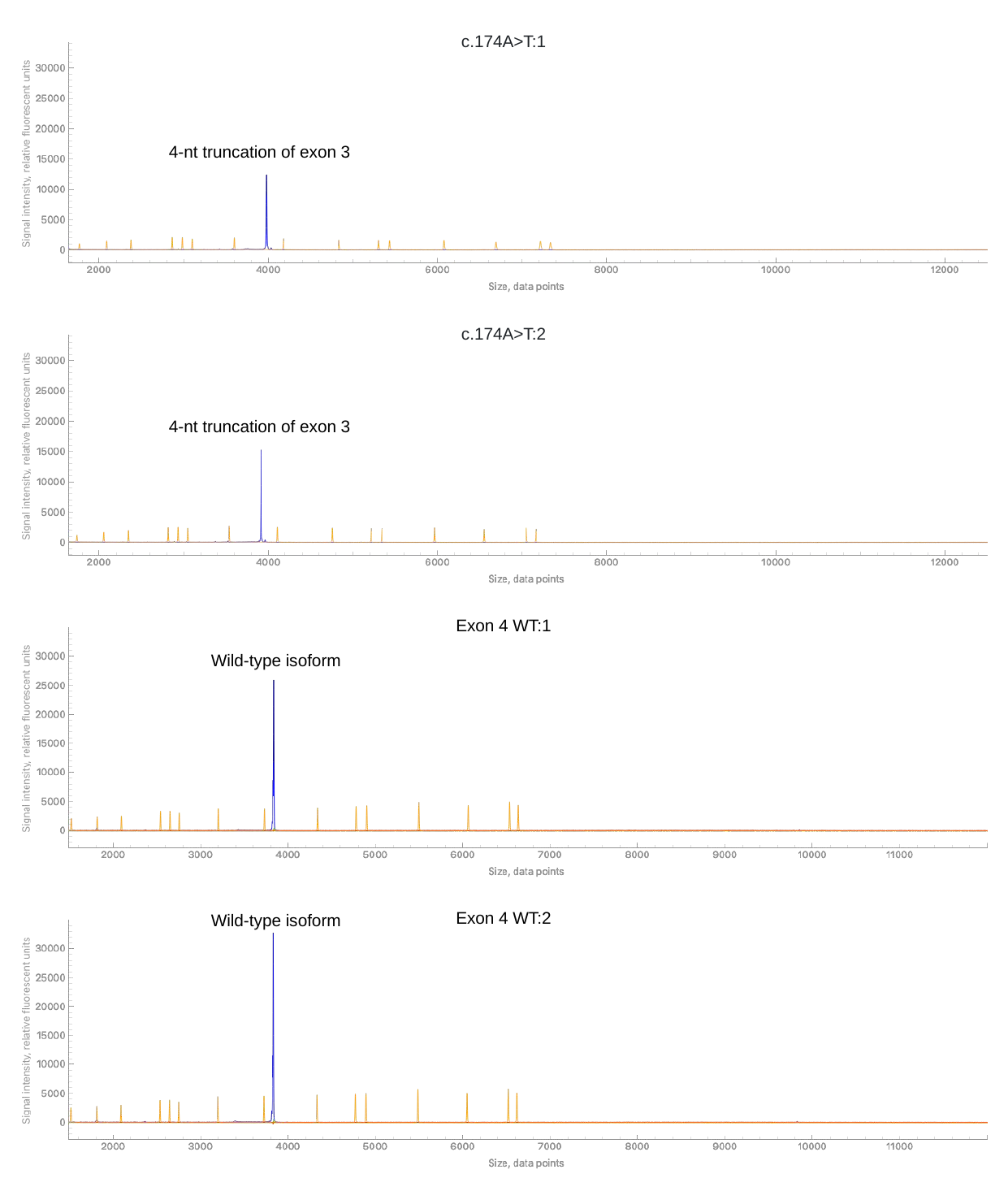

c.174A>T:1
4-nt truncation of exon 3
c.174A>T:2
4-nt truncation of exon 3
Exon 4 WT:1
Wild-type isoform
Wild-type isoform
Exon 4 WT:2

## Slide 4
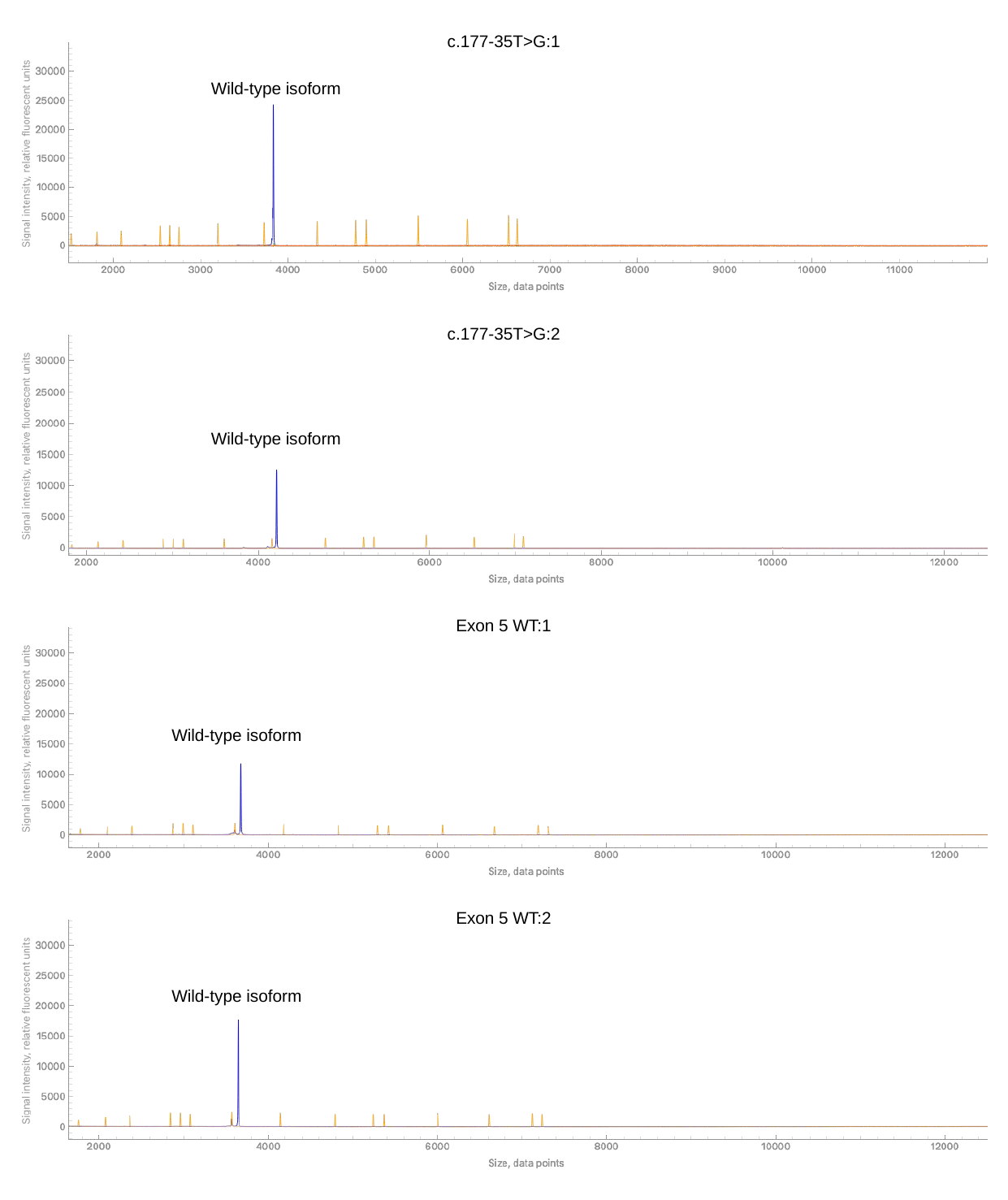

c.177-35T>G:1
Wild-type isoform
c.177-35T>G:2
Wild-type isoform
Exon 5 WT:1
Wild-type isoform
Exon 5 WT:2
Wild-type isoform

## Slide 5
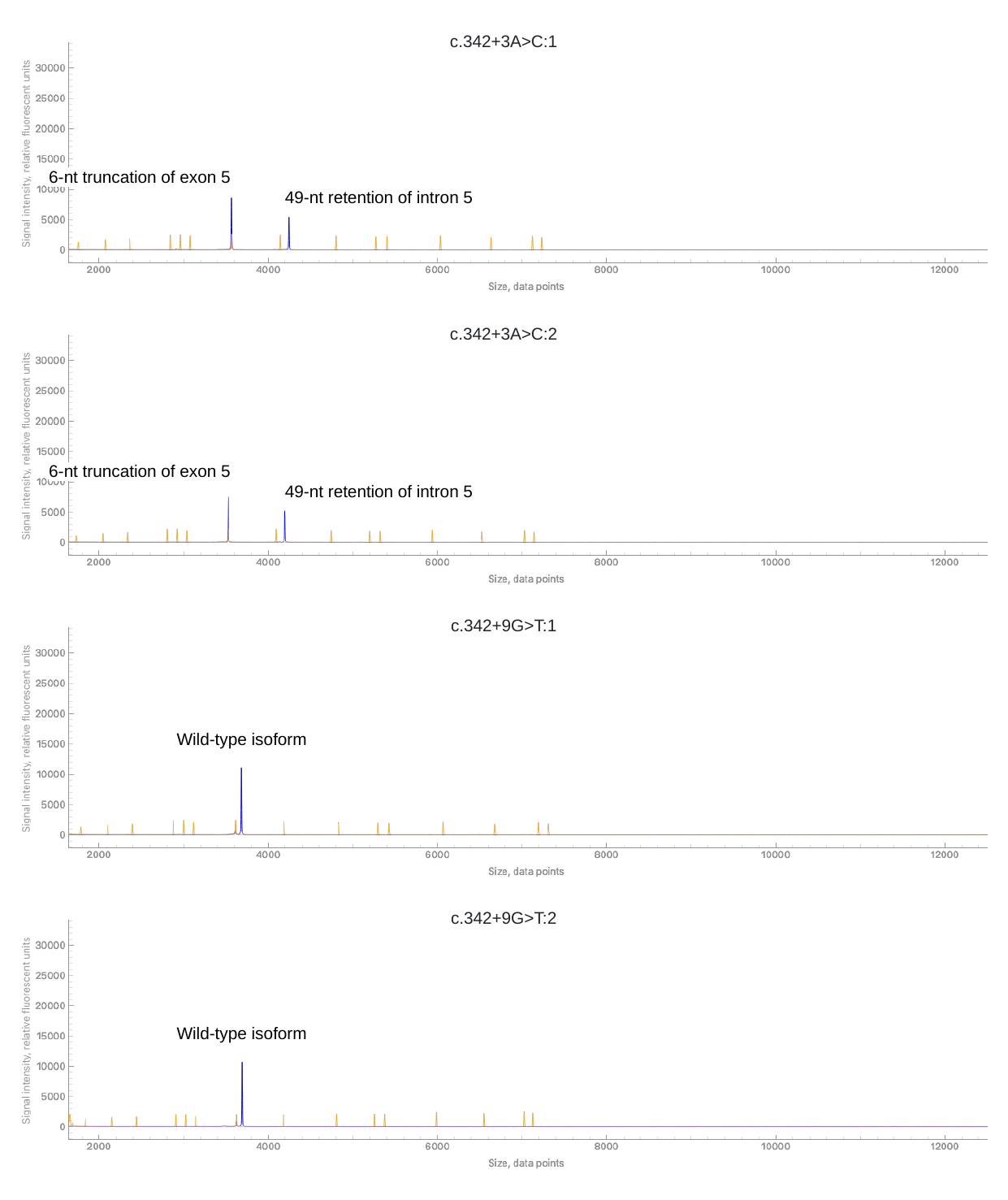

c.342+3A>C:1
6-nt truncation of exon 5
49-nt retention of intron 5
c.342+3A>C:2
6-nt truncation of exon 5
49-nt retention of intron 5
c.342+9G>T:1
Wild-type isoform
c.342+9G>T:2
Wild-type isoform

## Slide 6
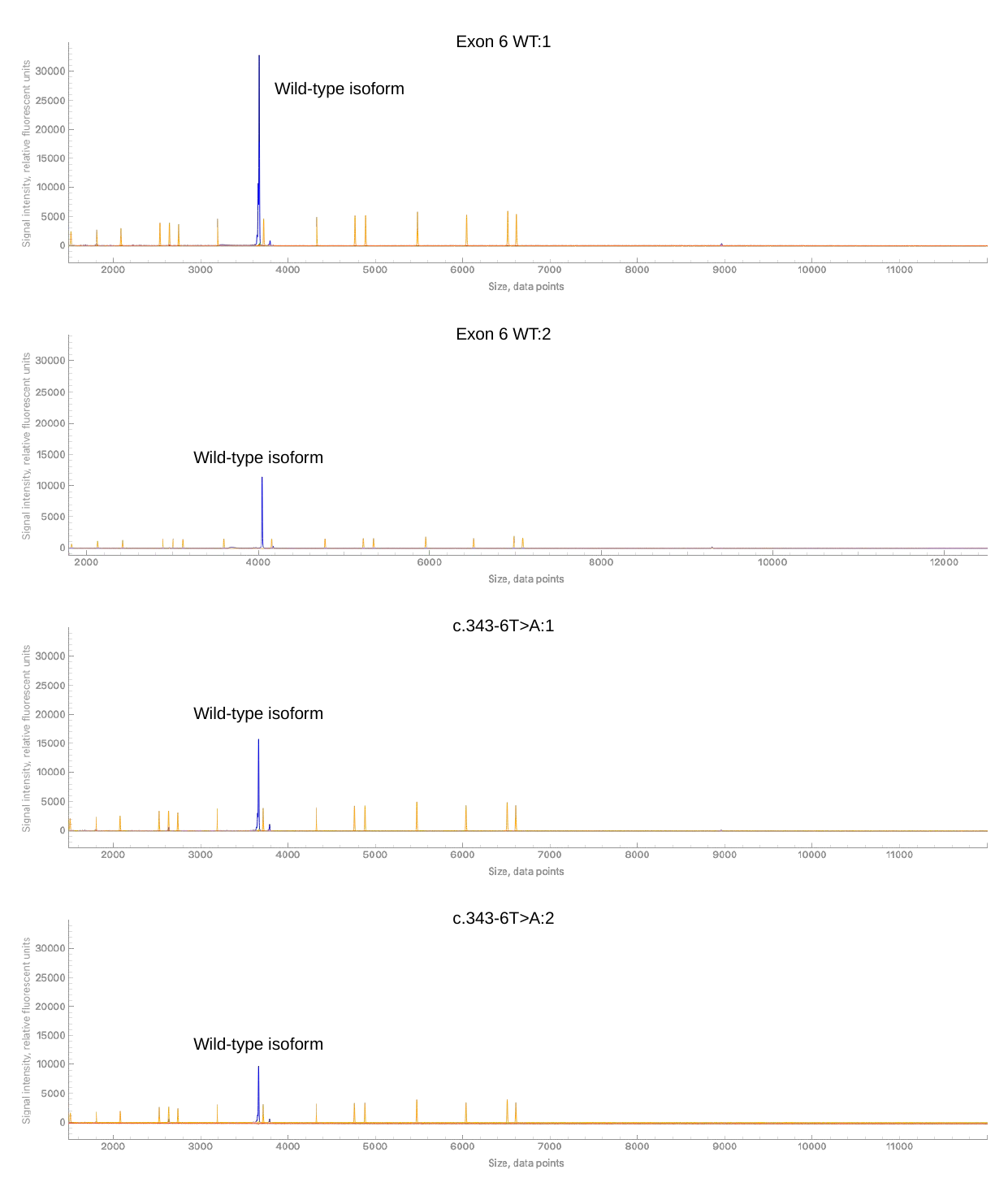

Exon 6 WT:1
Wild-type isoform
Exon 6 WT:2
Wild-type isoform
c.343-6T>A:1
Wild-type isoform
c.343-6T>A:2
Wild-type isoform

## Slide 7
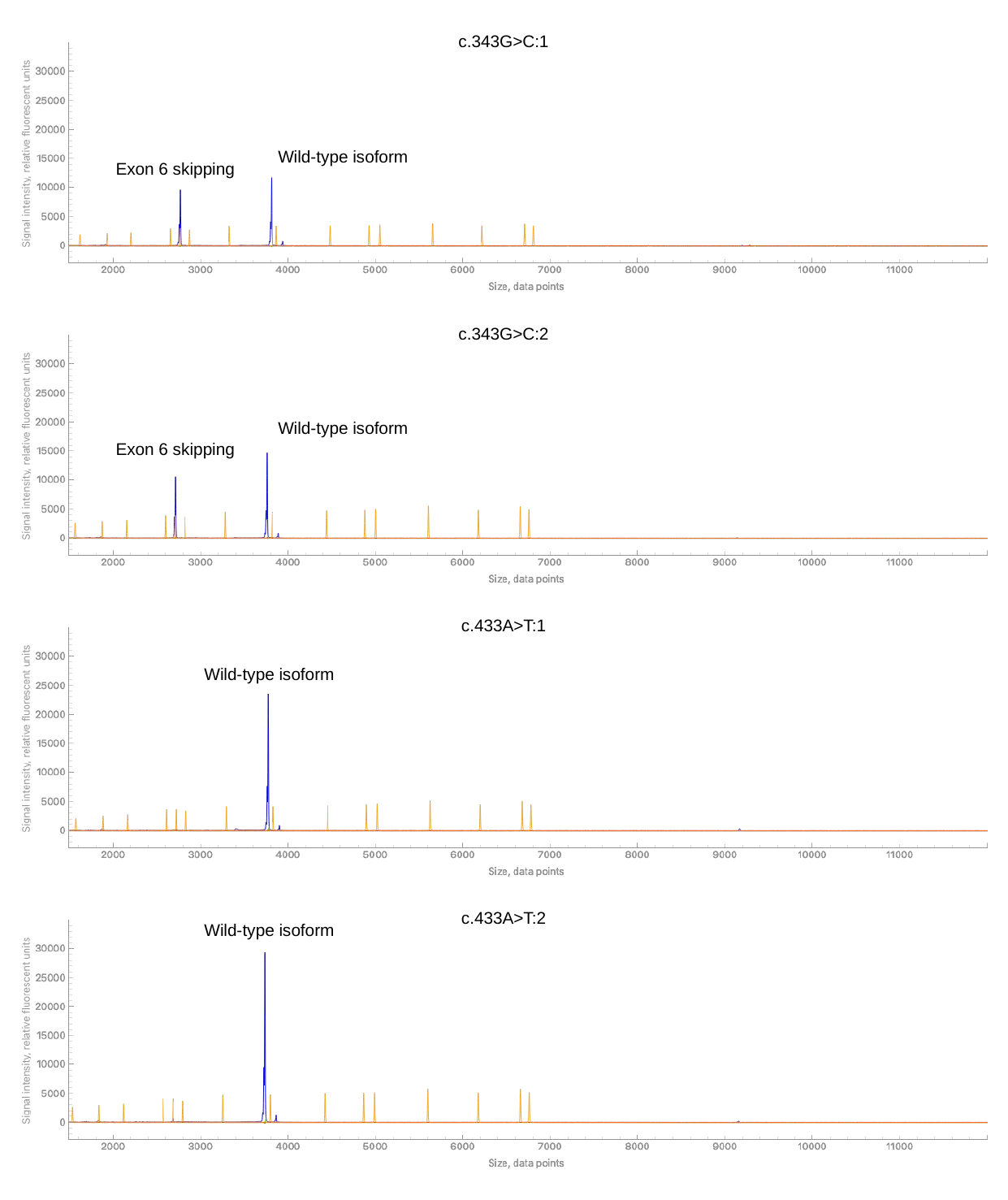

c.343G>C:1
Wild-type isoform
Exon 6 skipping
c.343G>C:2
Wild-type isoform
Exon 6 skipping
c.433A>T:1
Wild-type isoform
c.433A>T:2
Wild-type isoform

## Slide 8
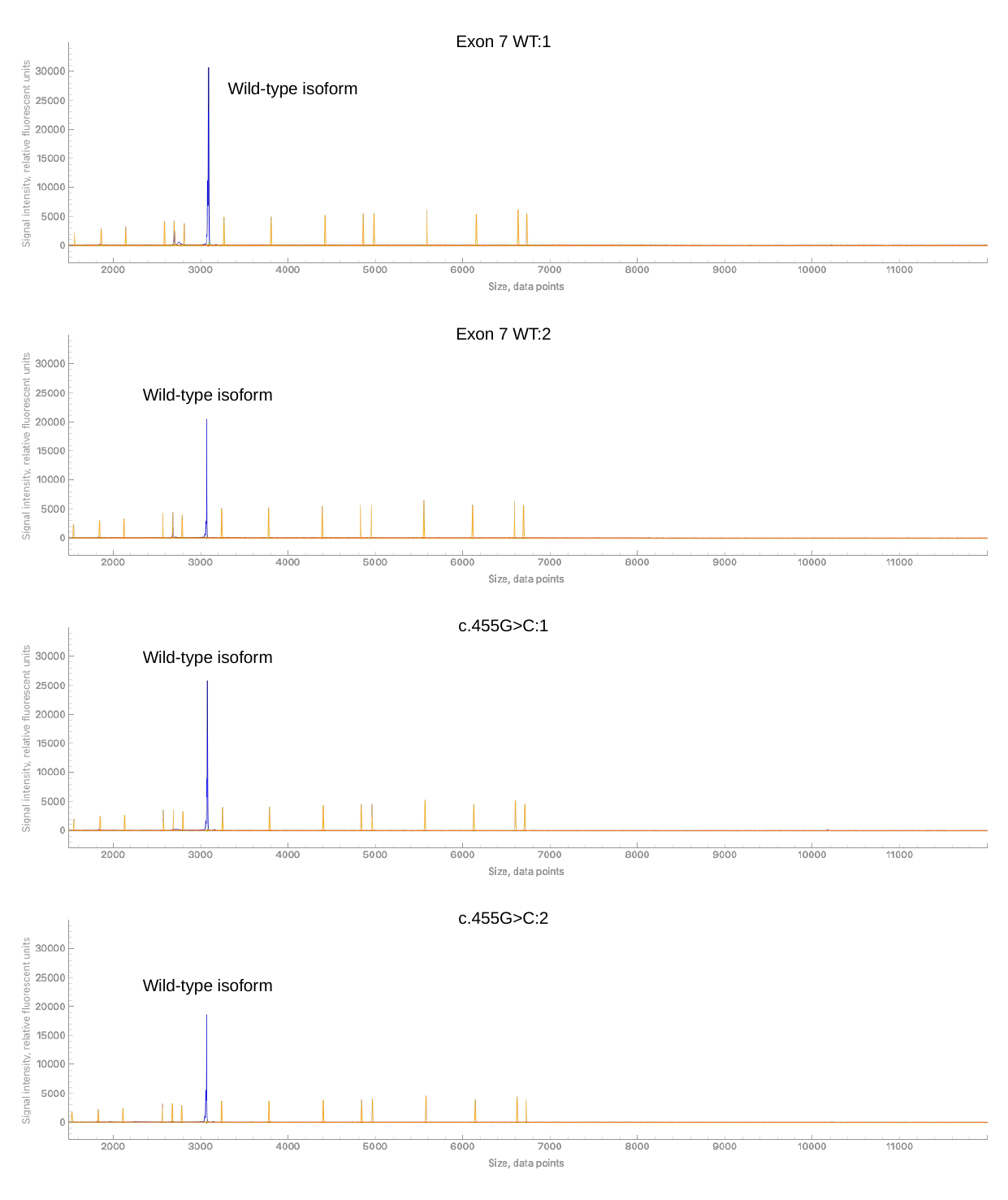

Exon 7 WT:1
Wild-type isoform
Exon 7 WT:2
Wild-type isoform
c.455G>C:1
Wild-type isoform
c.455G>C:2
Wild-type isoform

## Slide 9
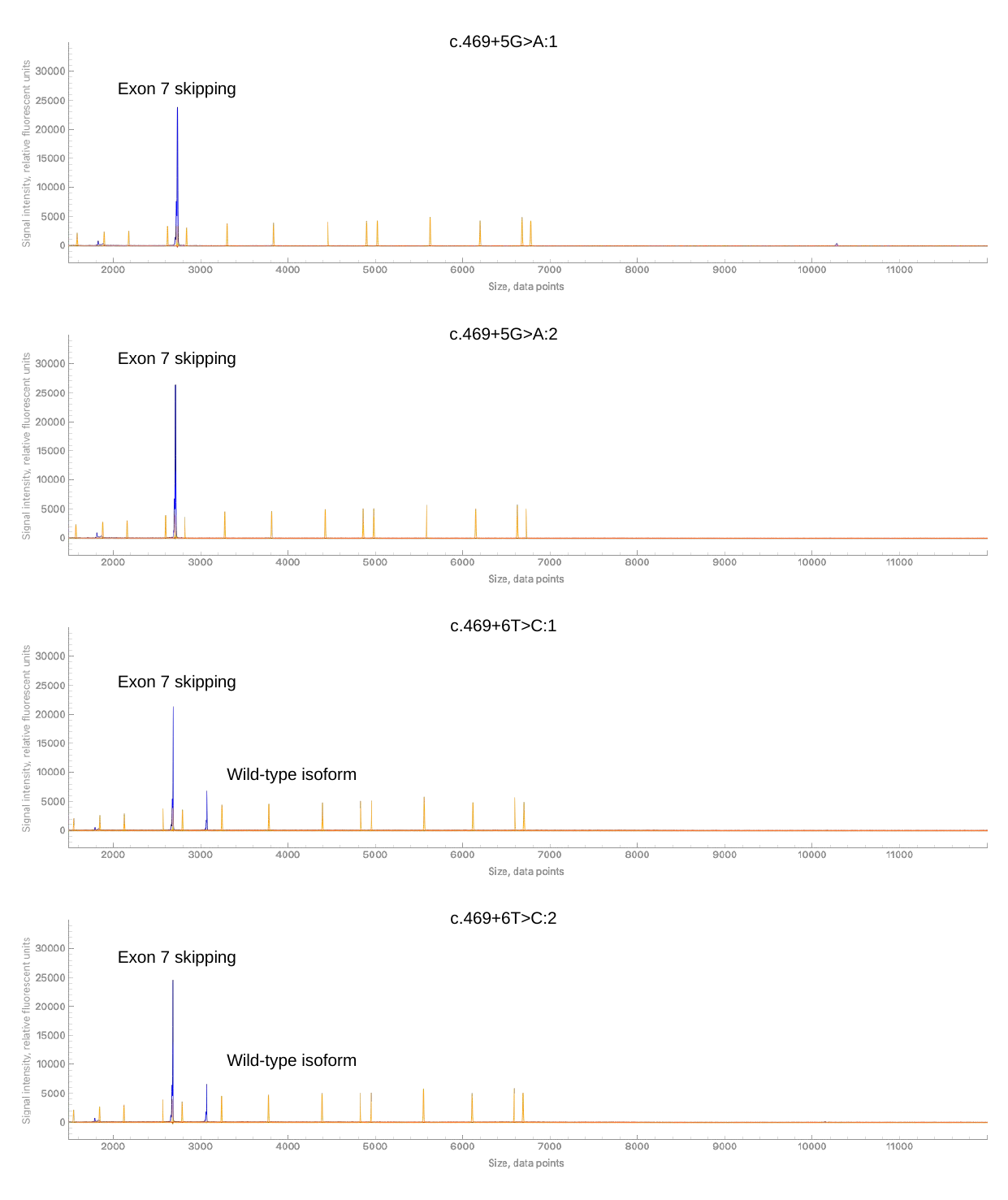

c.469+5G>A:1
Exon 7 skipping
c.469+5G>A:2
Exon 7 skipping
c.469+6T>C:1
Exon 7 skipping
Wild-type isoform
c.469+6T>C:2
Exon 7 skipping
Wild-type isoform

## Slide 10
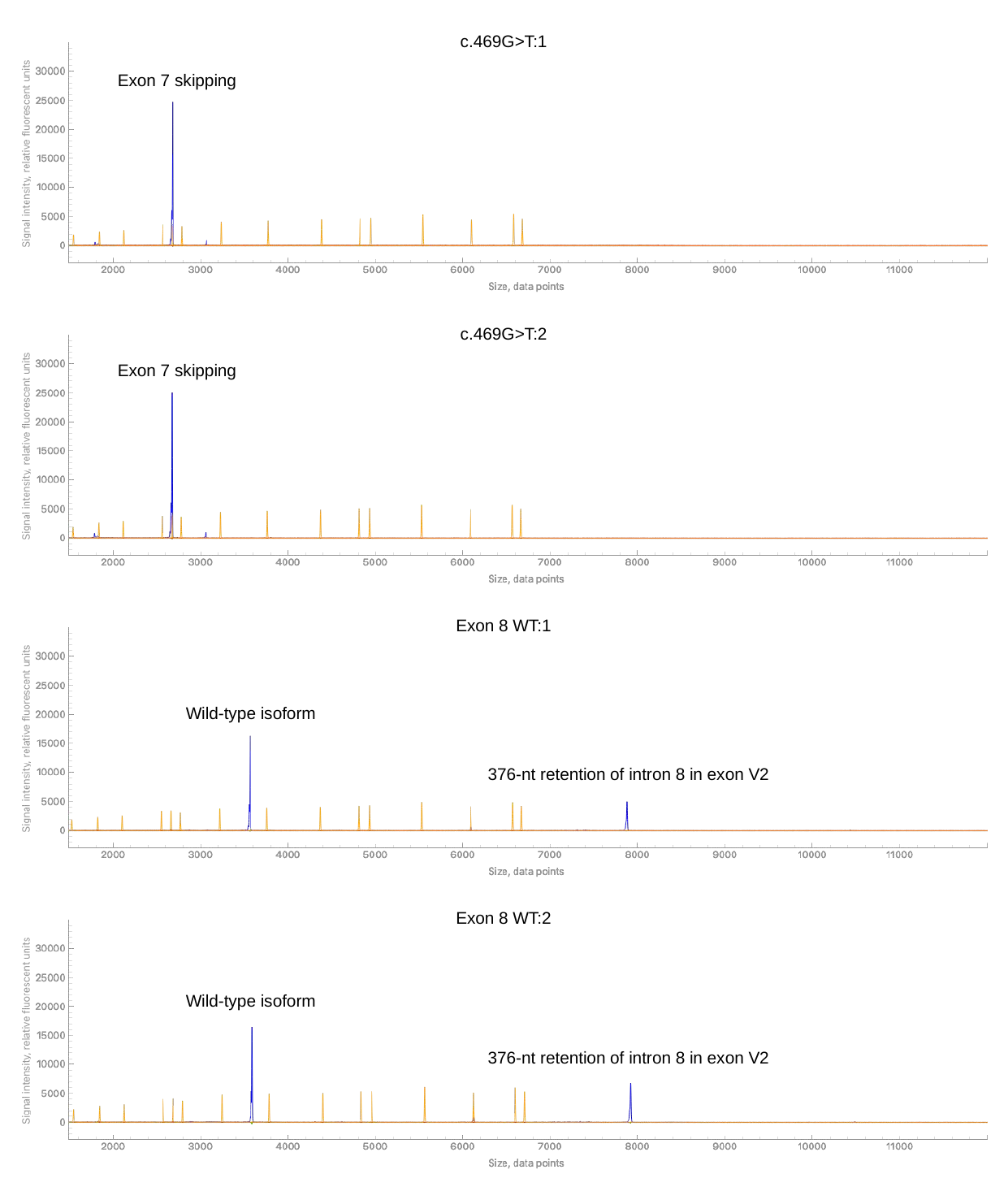

c.469G>T:1
Exon 7 skipping
c.469G>T:2
Exon 7 skipping
Exon 8 WT:1
Wild-type isoform
376-nt retention of intron 8 in exon V2
Exon 8 WT:2
Wild-type isoform
376-nt retention of intron 8 in exon V2

## Slide 11
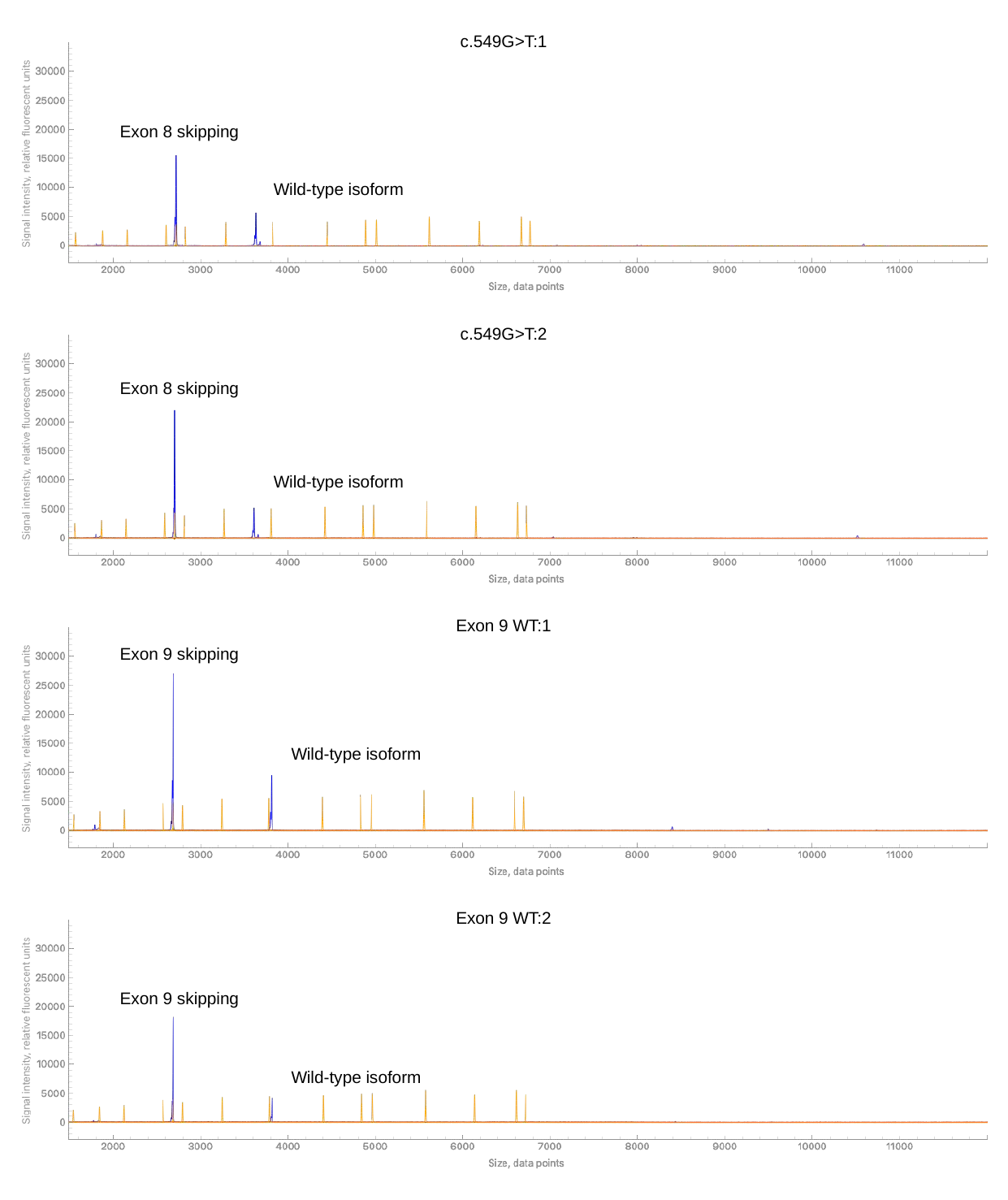

c.549G>T:1
Exon 8 skipping
Wild-type isoform
c.549G>T:2
Exon 8 skipping
Wild-type isoform
Exon 9 WT:1
Exon 9 skipping
Wild-type isoform
Exon 9 WT:2
Exon 9 skipping
Wild-type isoform

## Slide 12
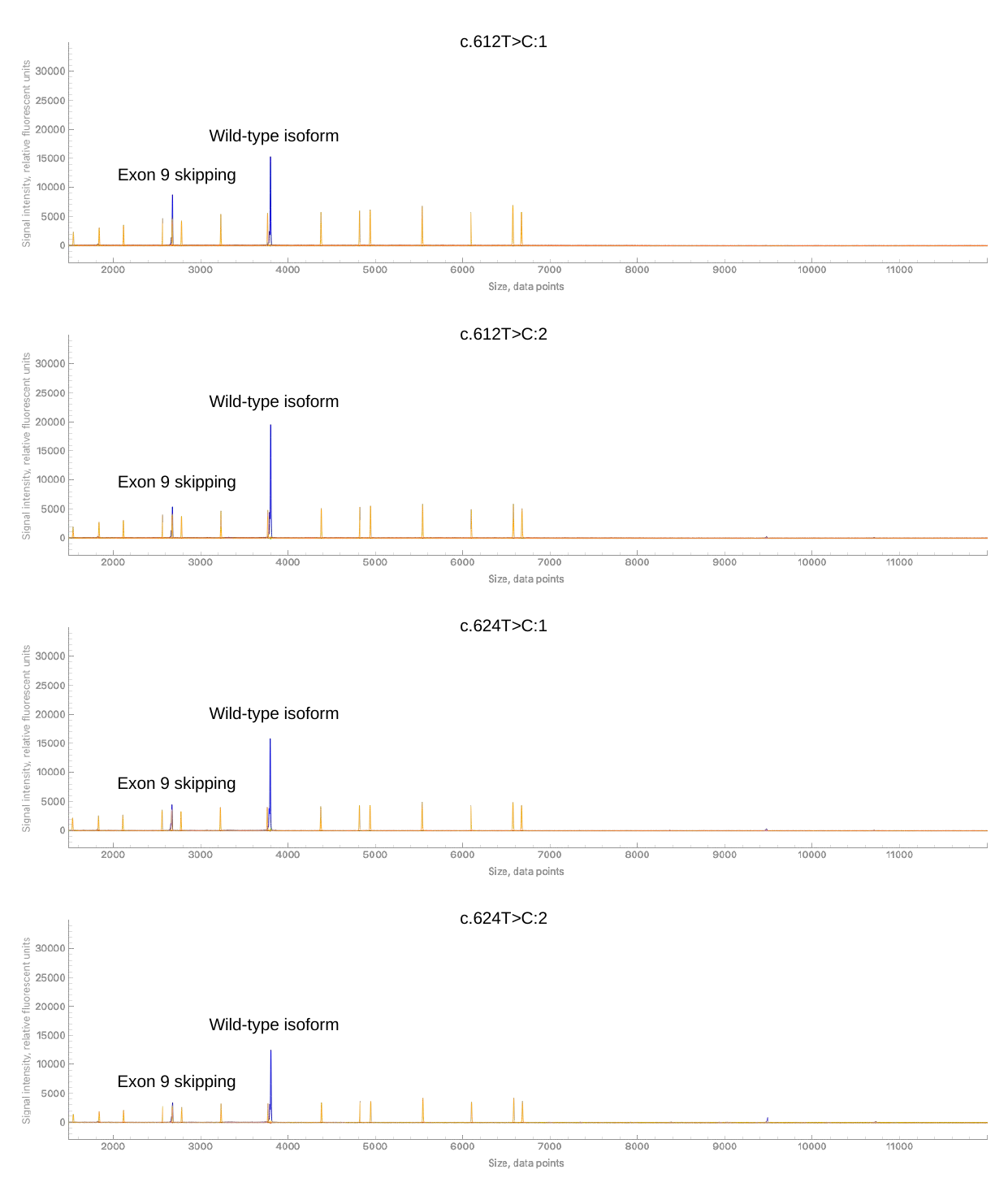

c.612T>C:1
Wild-type isoform
Exon 9 skipping
c.612T>C:2
Wild-type isoform
Exon 9 skipping
c.624T>C:1
Wild-type isoform
Exon 9 skipping
c.624T>C:2
Wild-type isoform
Exon 9 skipping

## Slide 13
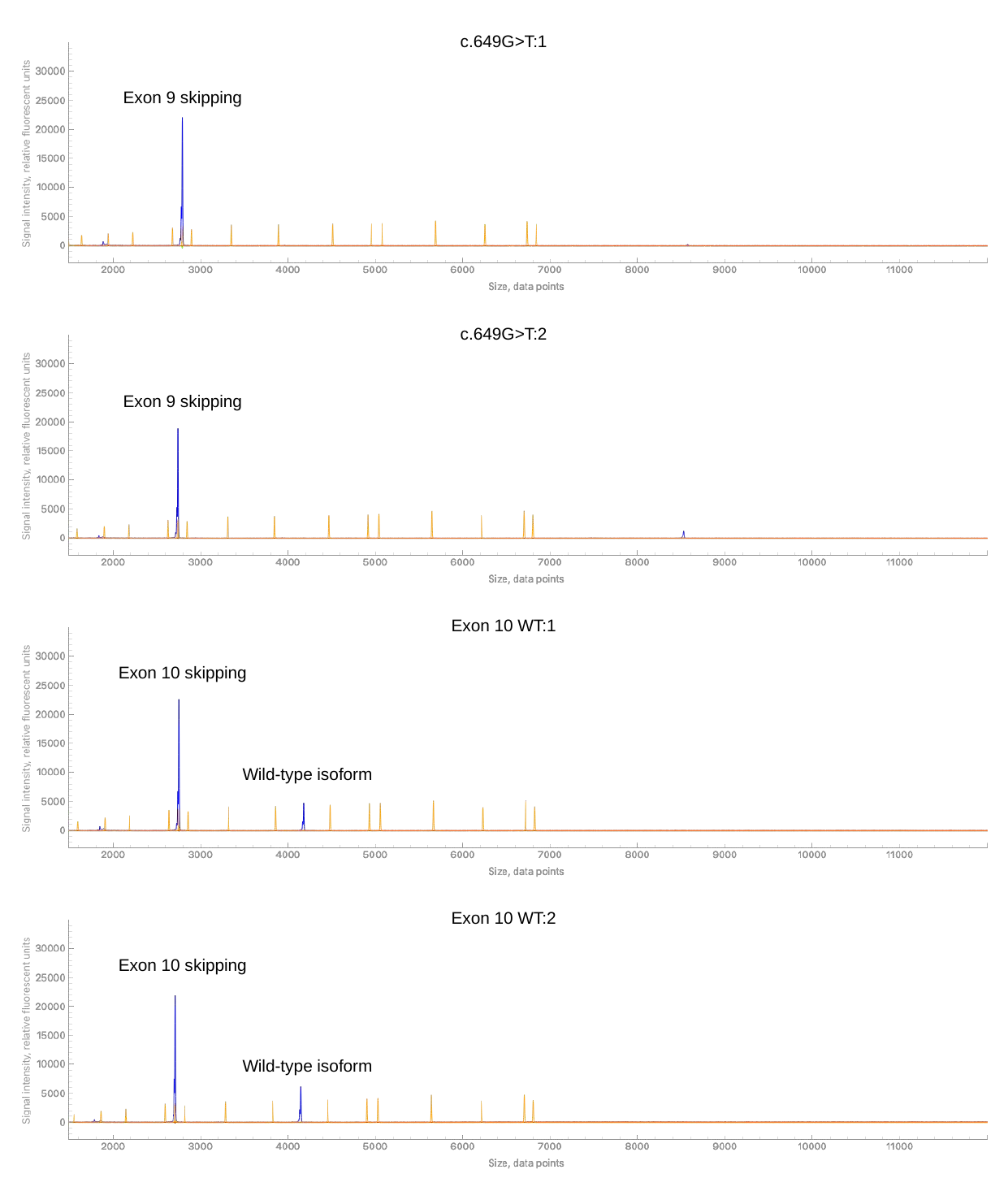

c.649G>T:1
Exon 9 skipping
c.649G>T:2
Exon 9 skipping
Exon 10 WT:1
Exon 10 skipping
Wild-type isoform
Exon 10 WT:2
Exon 10 skipping
Wild-type isoform

## Slide 14
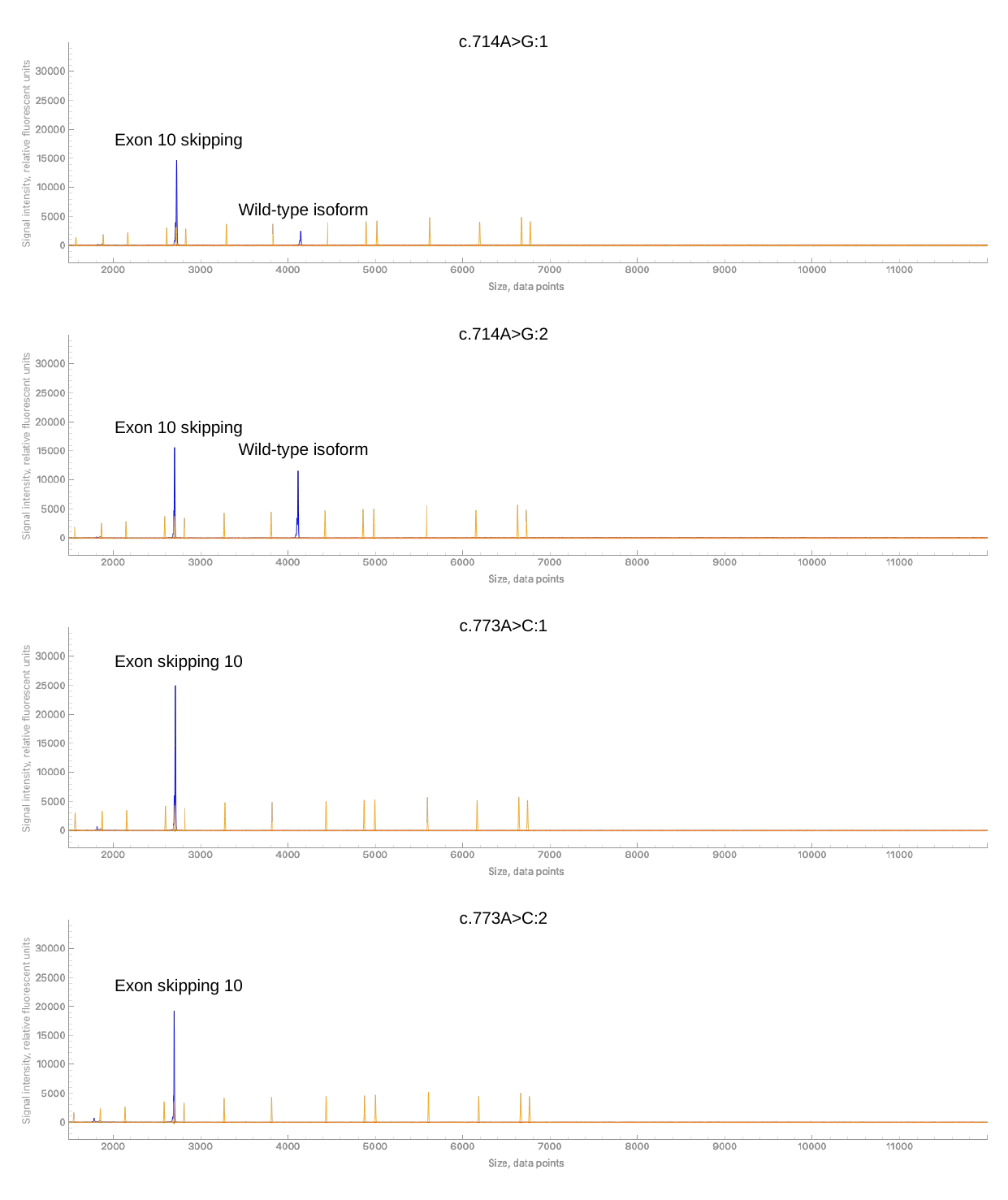

c.714A>G:1
Exon 10 skipping
Wild-type isoform
c.714A>G:2
Exon 10 skipping
Wild-type isoform
c.773A>C:1
Exon skipping 10
c.773A>C:2
Exon skipping 10

## Slide 15
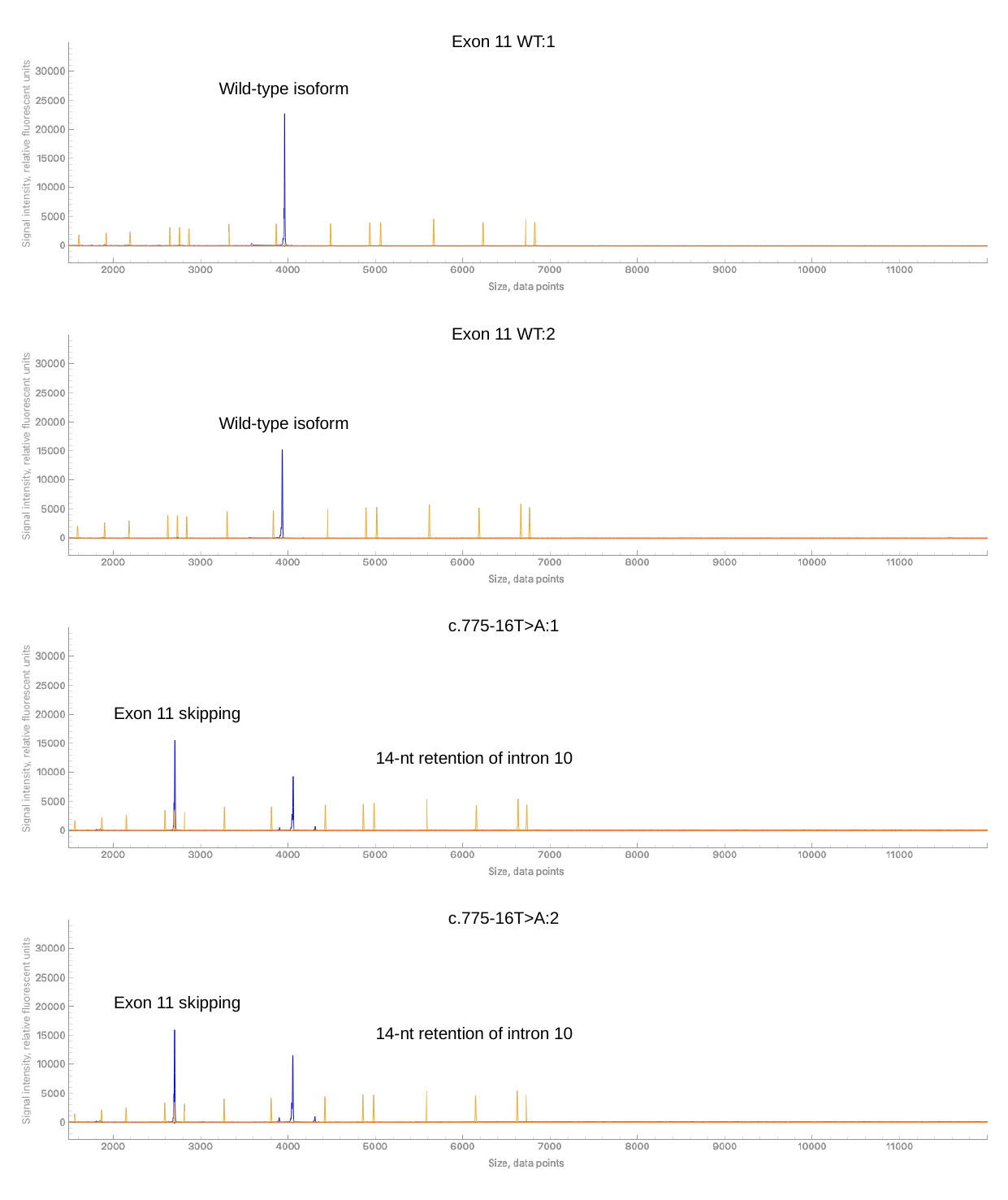

Exon 11 WT:1
Wild-type isoform
Exon 11 WT:2
Wild-type isoform
c.775-16T>A:1
Exon 11 skipping
14-nt retention of intron 10
c.775-16T>A:2
Exon 11 skipping
14-nt retention of intron 10

## Slide 16
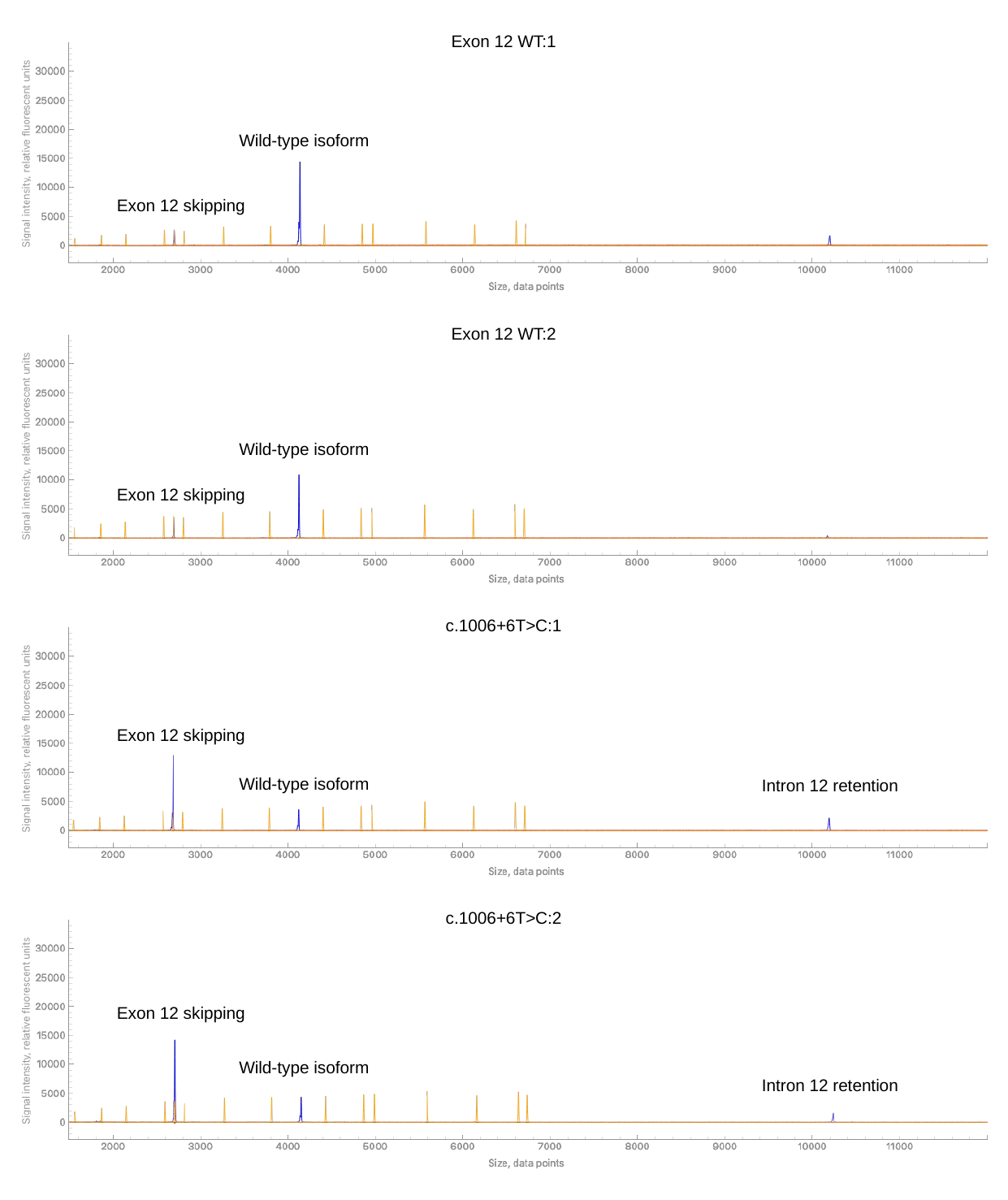

Exon 12 WT:1
Wild-type isoform
Exon 12 skipping
Exon 12 WT:2
Wild-type isoform
Exon 12 skipping
c.1006+6T>C:1
Exon 12 skipping
Wild-type isoform
Intron 12 retention
c.1006+6T>C:2
Exon 12 skipping
Wild-type isoform
Intron 12 retention

## Slide 17
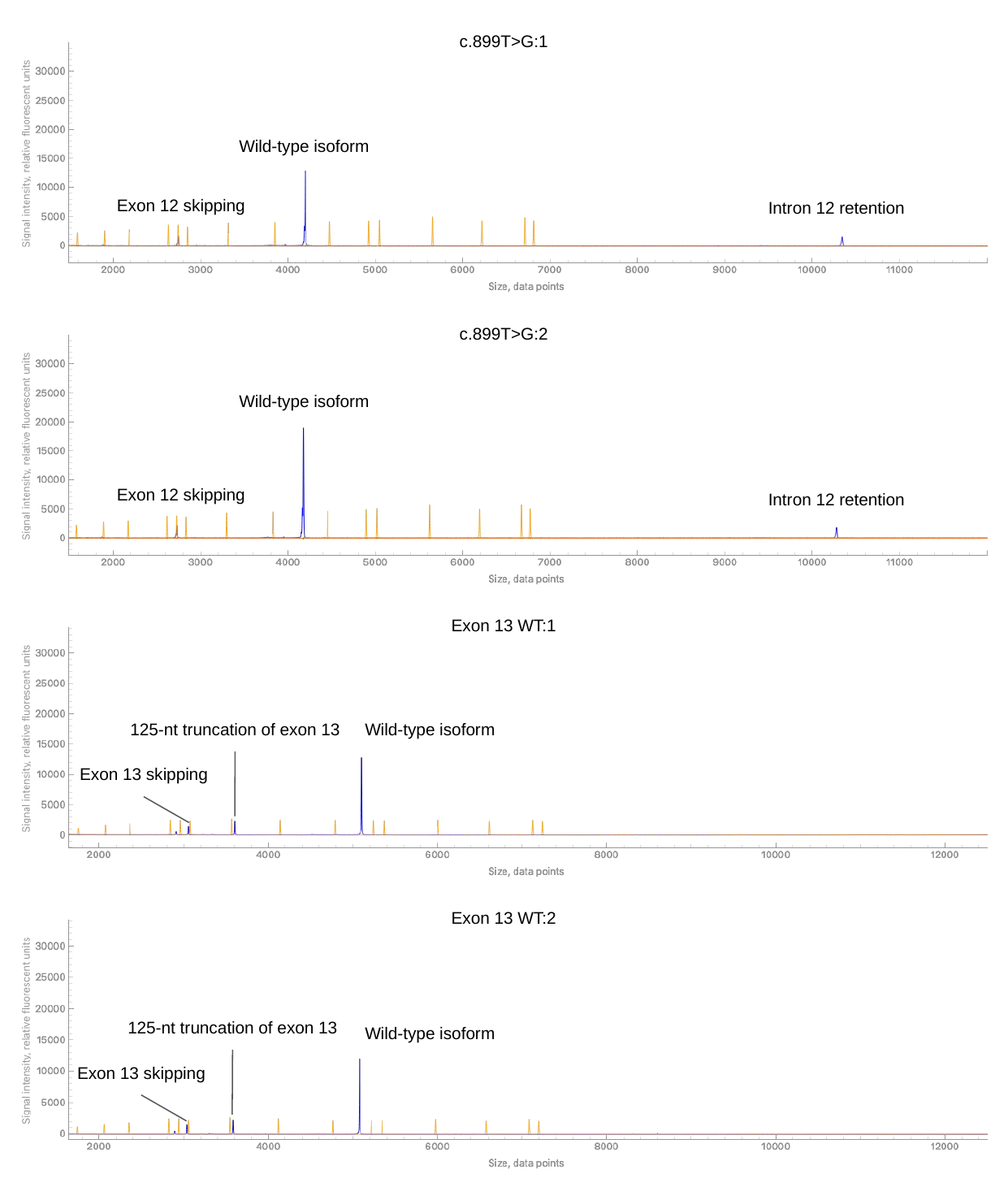

c.899T>G:1
Wild-type isoform
Exon 12 skipping
Intron 12 retention
c.899T>G:2
Wild-type isoform
Exon 12 skipping
Intron 12 retention
Exon 13 WT:1
125-nt truncation of exon 13
Wild-type isoform
Exon 13 skipping
Exon 13 WT:2
125-nt truncation of exon 13
Wild-type isoform
Exon 13 skipping

## Slide 18
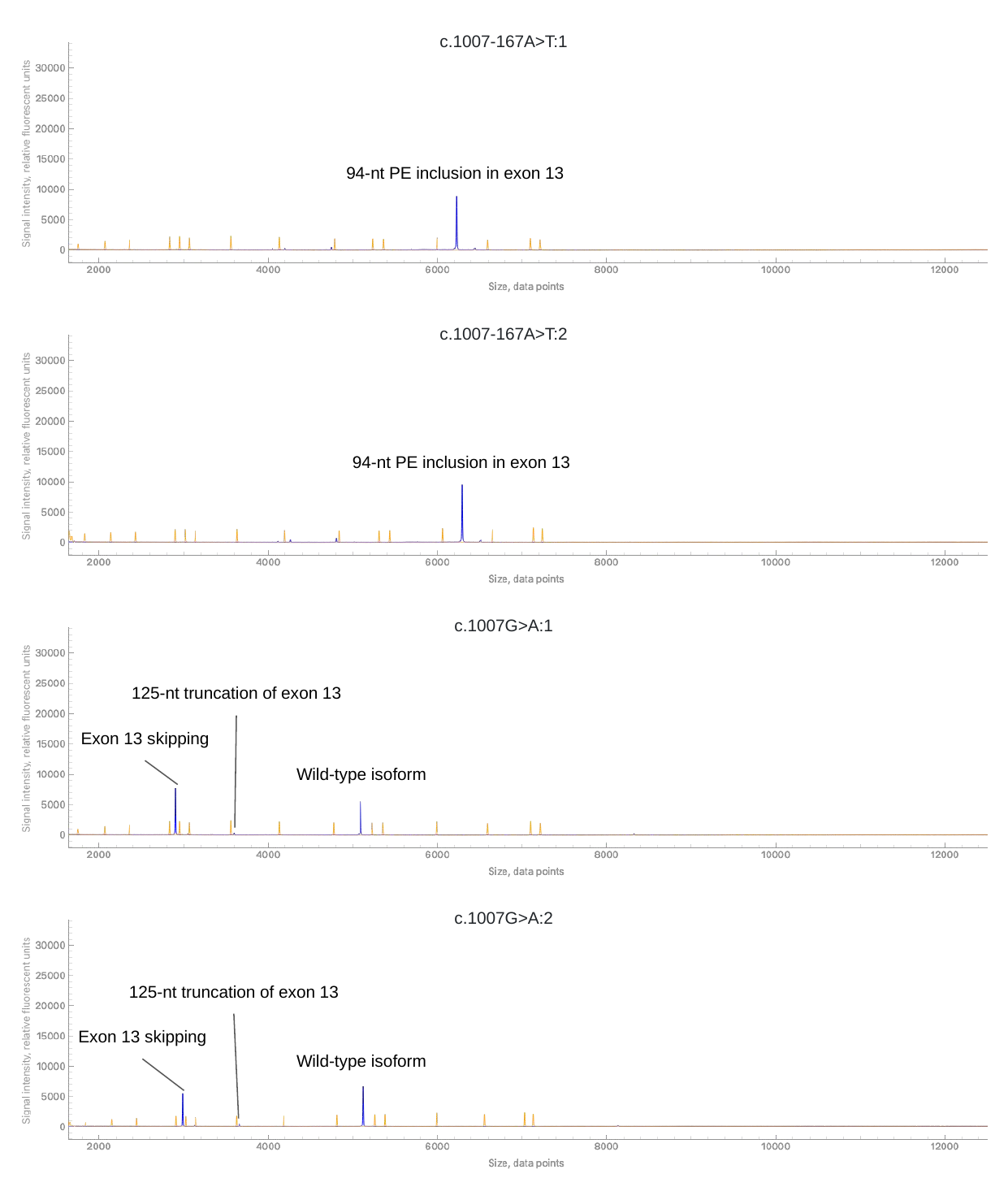

c.1007-167A>T:1
94-nt PE inclusion in exon 13
c.1007-167A>T:2
94-nt PE inclusion in exon 13
c.1007G>A:1
125-nt truncation of exon 13
Exon 13 skipping
Wild-type isoform
c.1007G>A:2
125-nt truncation of exon 13
Exon 13 skipping
Wild-type isoform

## Slide 19
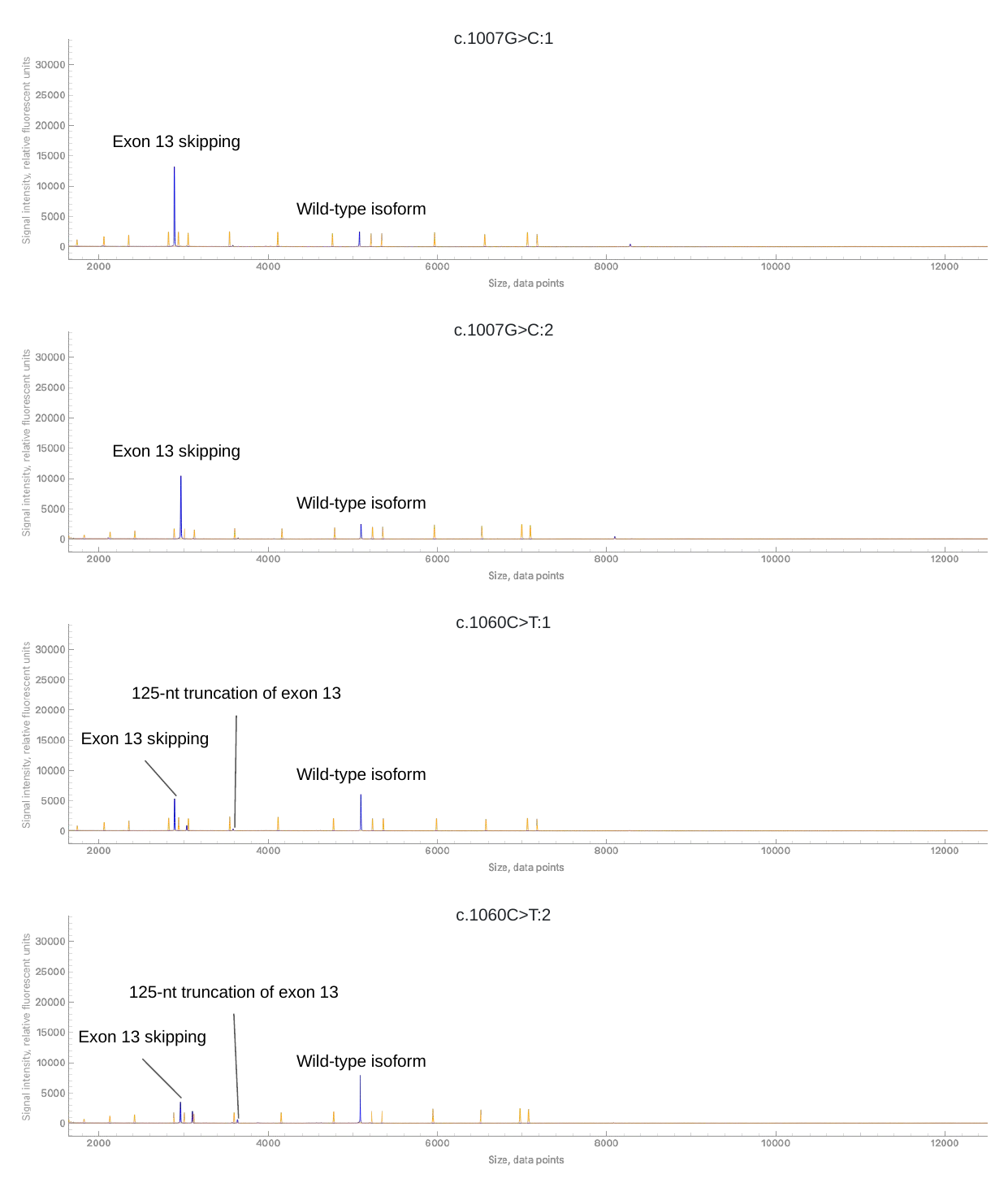

c.1007G>C:1
Exon 13 skipping
Wild-type isoform
c.1007G>C:2
Exon 13 skipping
Wild-type isoform
c.1060C>T:1
125-nt truncation of exon 13
Exon 13 skipping
Wild-type isoform
c.1060C>T:2
125-nt truncation of exon 13
Exon 13 skipping
Wild-type isoform

## Slide 20
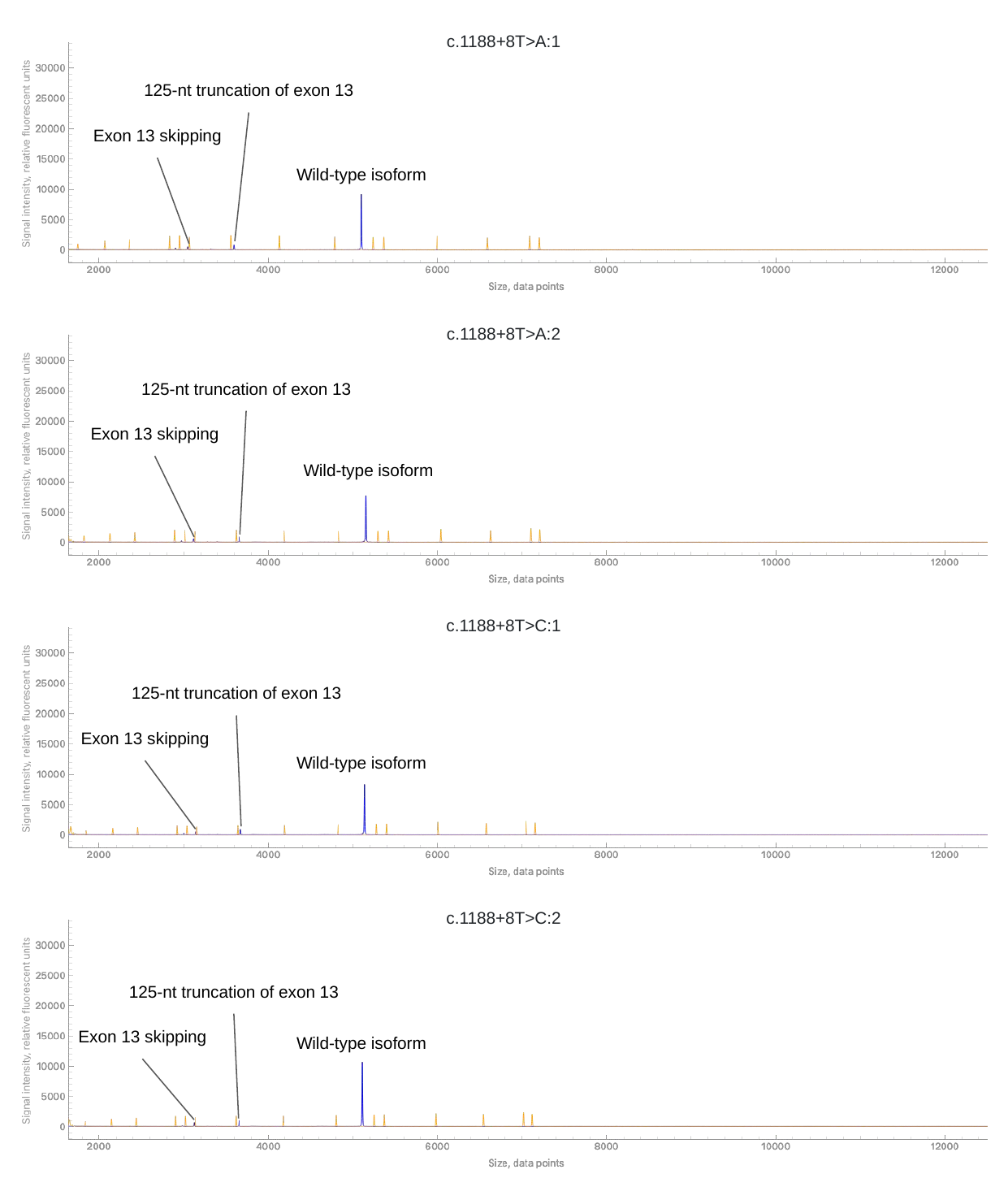

c.1188+8T>A:1
125-nt truncation of exon 13
Exon 13 skipping
Wild-type isoform
c.1188+8T>A:2
125-nt truncation of exon 13
Exon 13 skipping
Wild-type isoform
c.1188+8T>C:1
125-nt truncation of exon 13
Exon 13 skipping
Wild-type isoform
c.1188+8T>C:2
125-nt truncation of exon 13
Exon 13 skipping
Wild-type isoform

## Slide 21
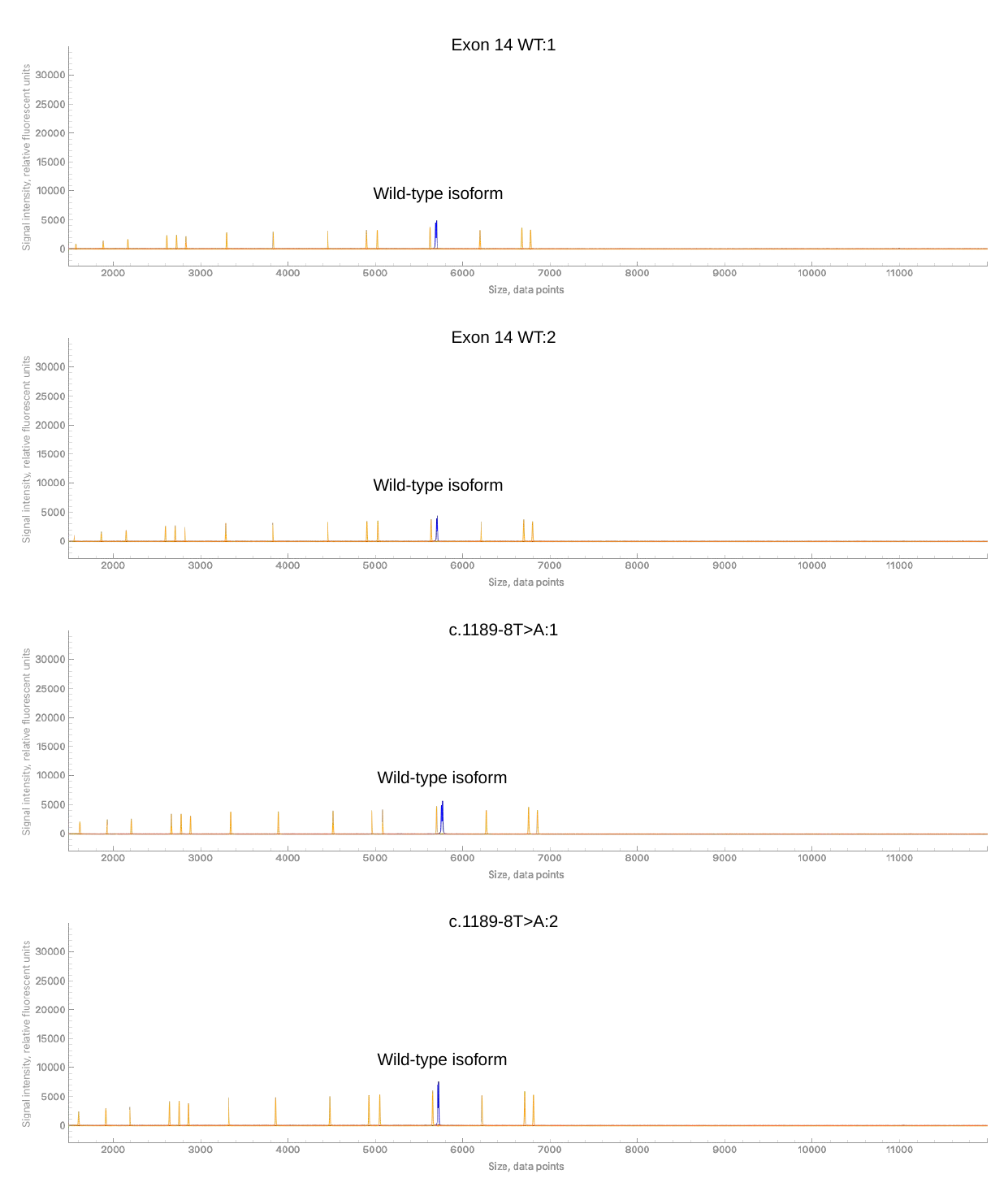

Exon 14 WT:1
Wild-type isoform
Exon 14 WT:2
Wild-type isoform
c.1189-8T>A:1
Wild-type isoform
c.1189-8T>A:2
Wild-type isoform
