# Supplementary material for: Functional Characterization of HGD Gene Variants by Minigene Splicing Assay
Source: Int J Mol Sci. 2025 Oct 31;26(21):10639. doi: 10.3390/ijms262110639 (PMC12608343; doi:10.3390/ijms262110639)
Supplement: Supplementary file 1 [file ijms-26-10639-s001.zip › Supplementary/Supplementary S3 (PAAG).pptx]

## Slide 1
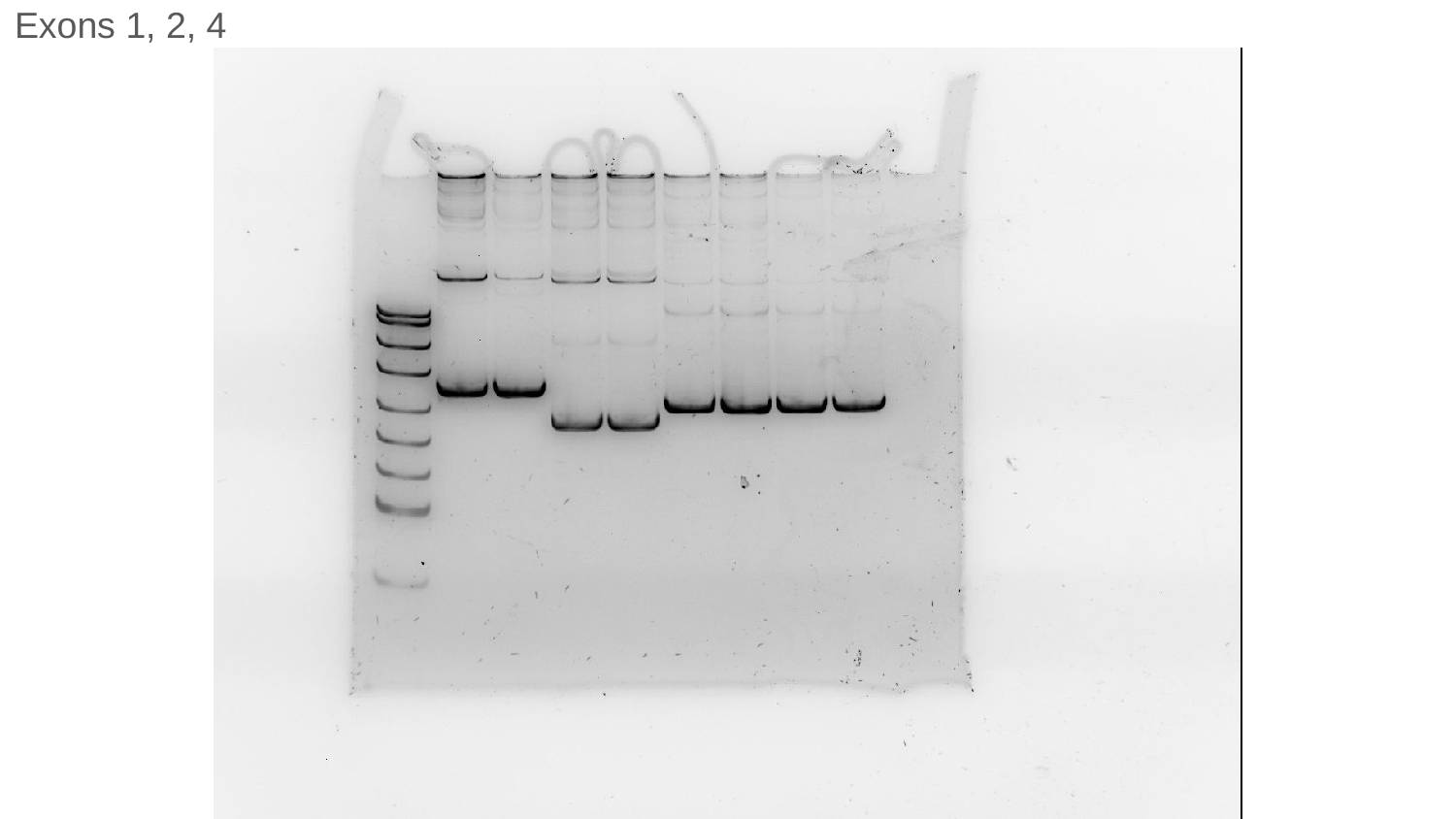

Exons 1, 2, 4

## Slide 2
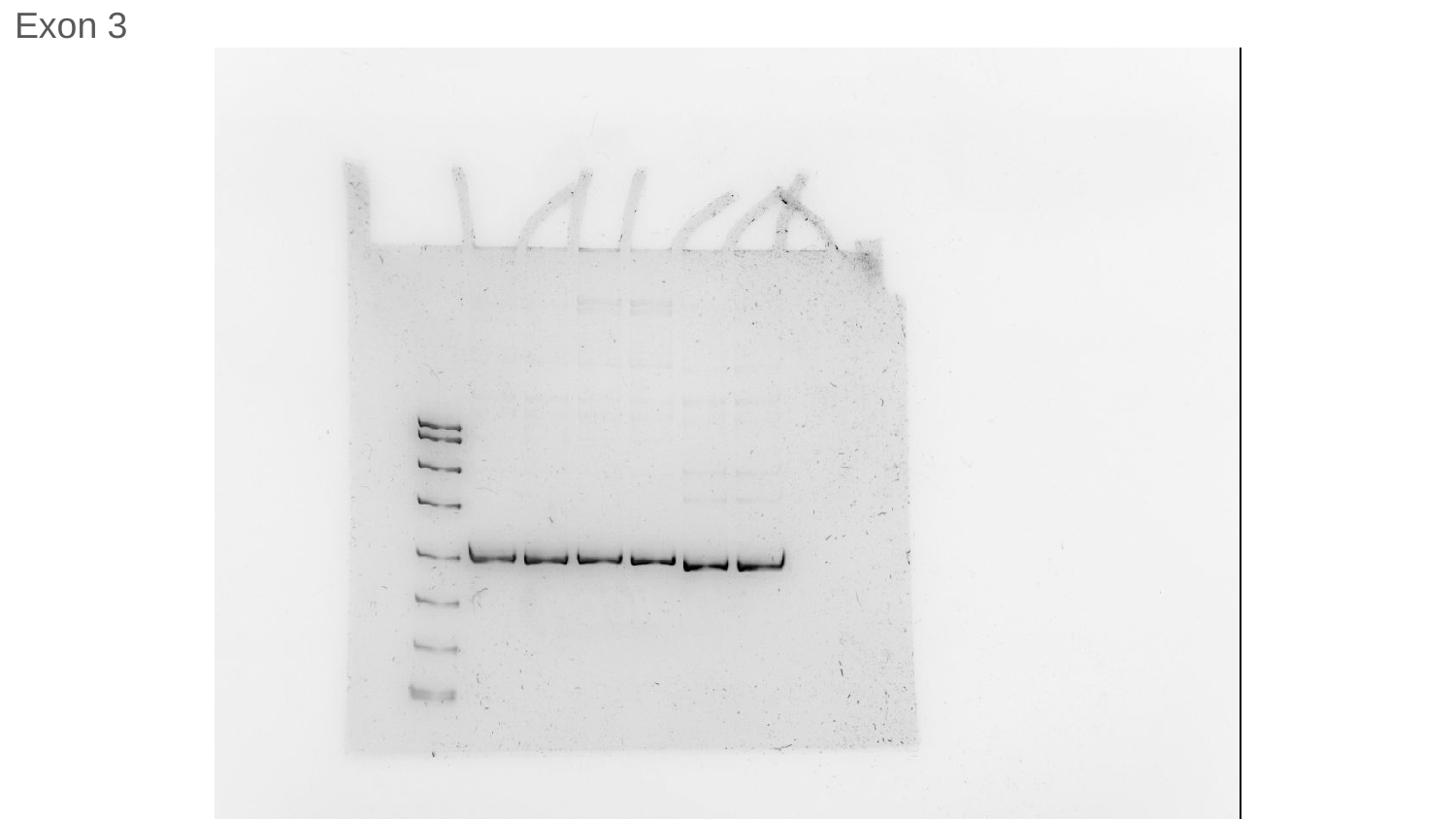

Exon 3

## Slide 3
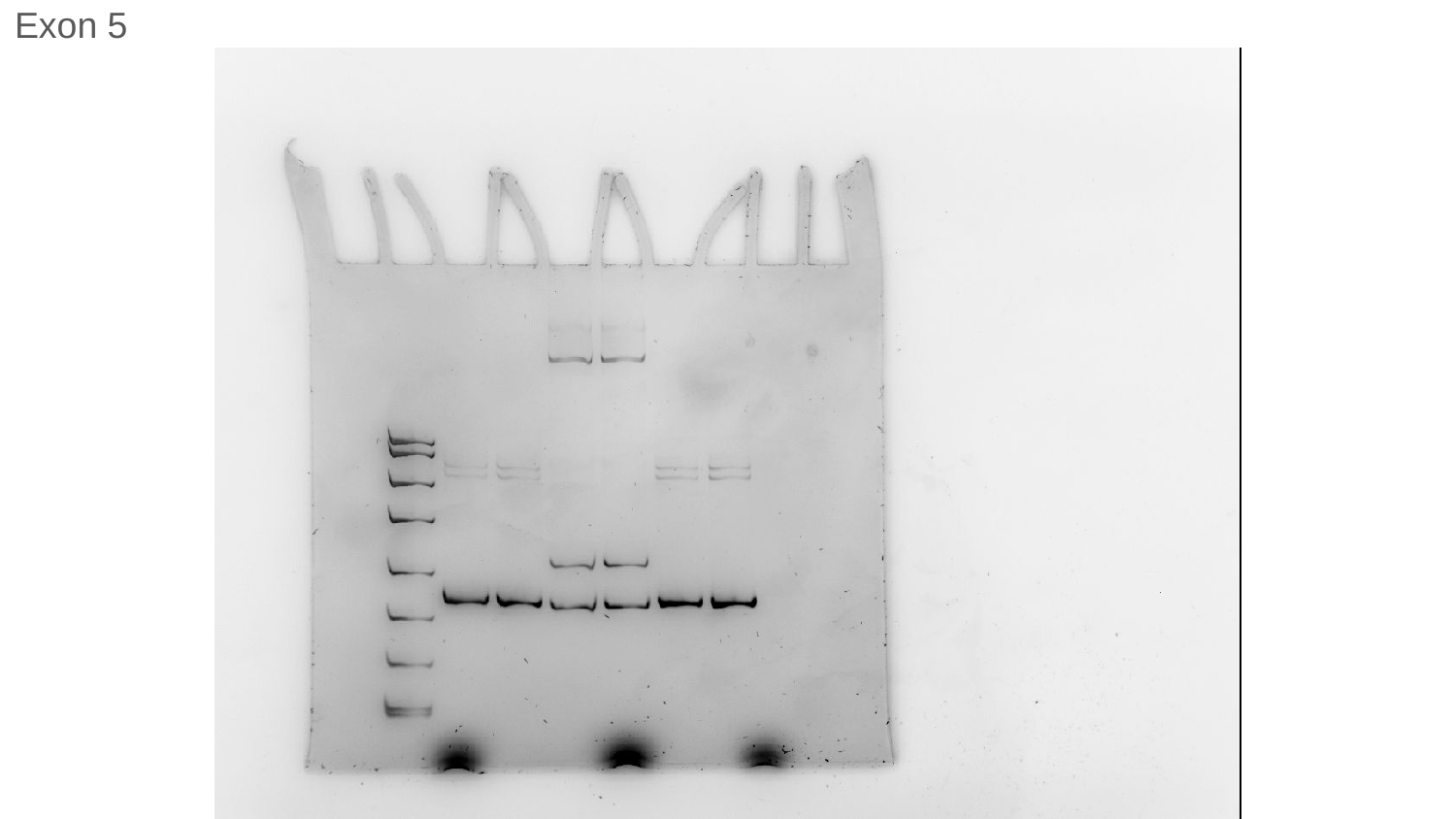

Exon 5

## Slide 4
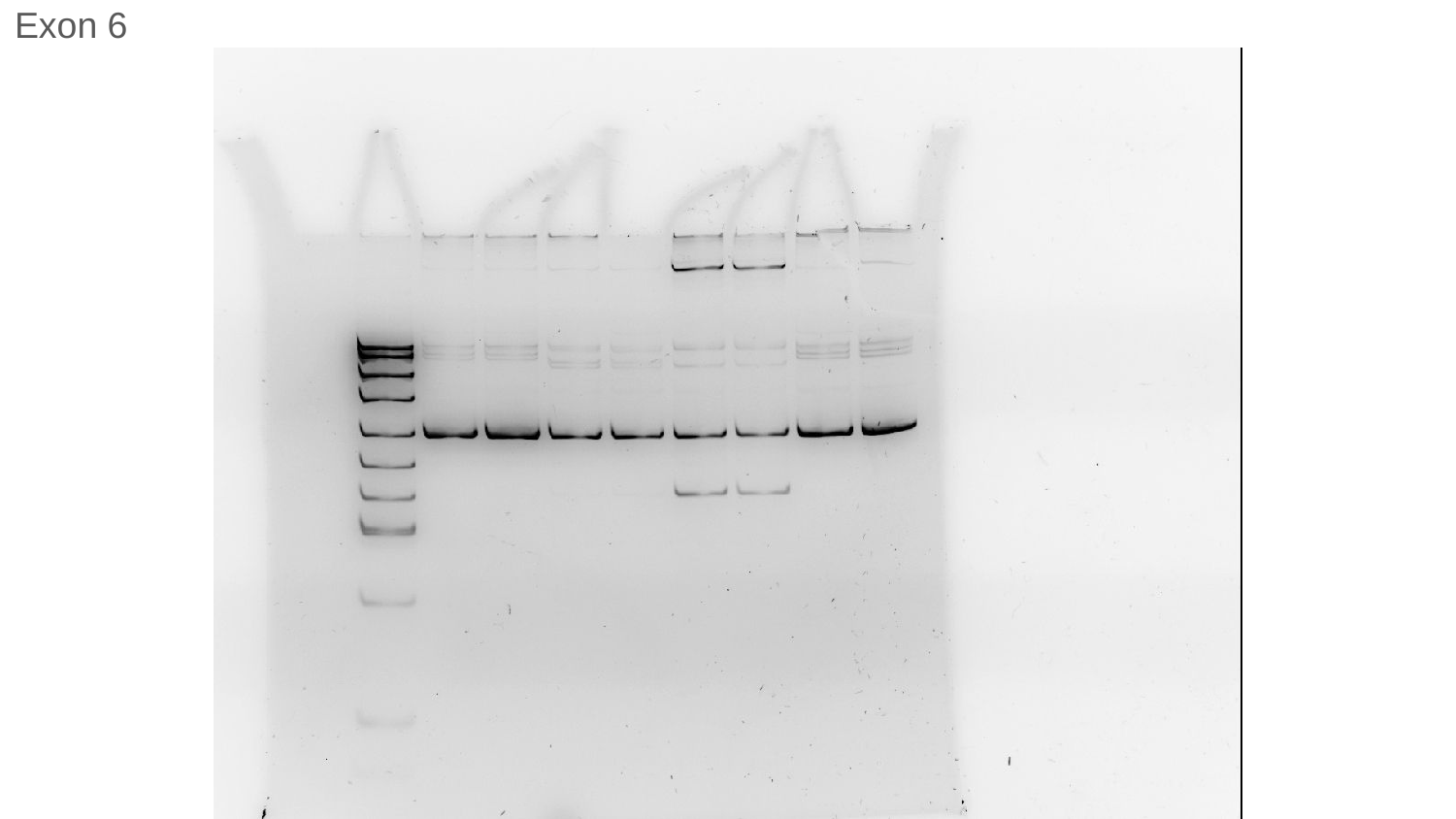

Exon 6

## Slide 5
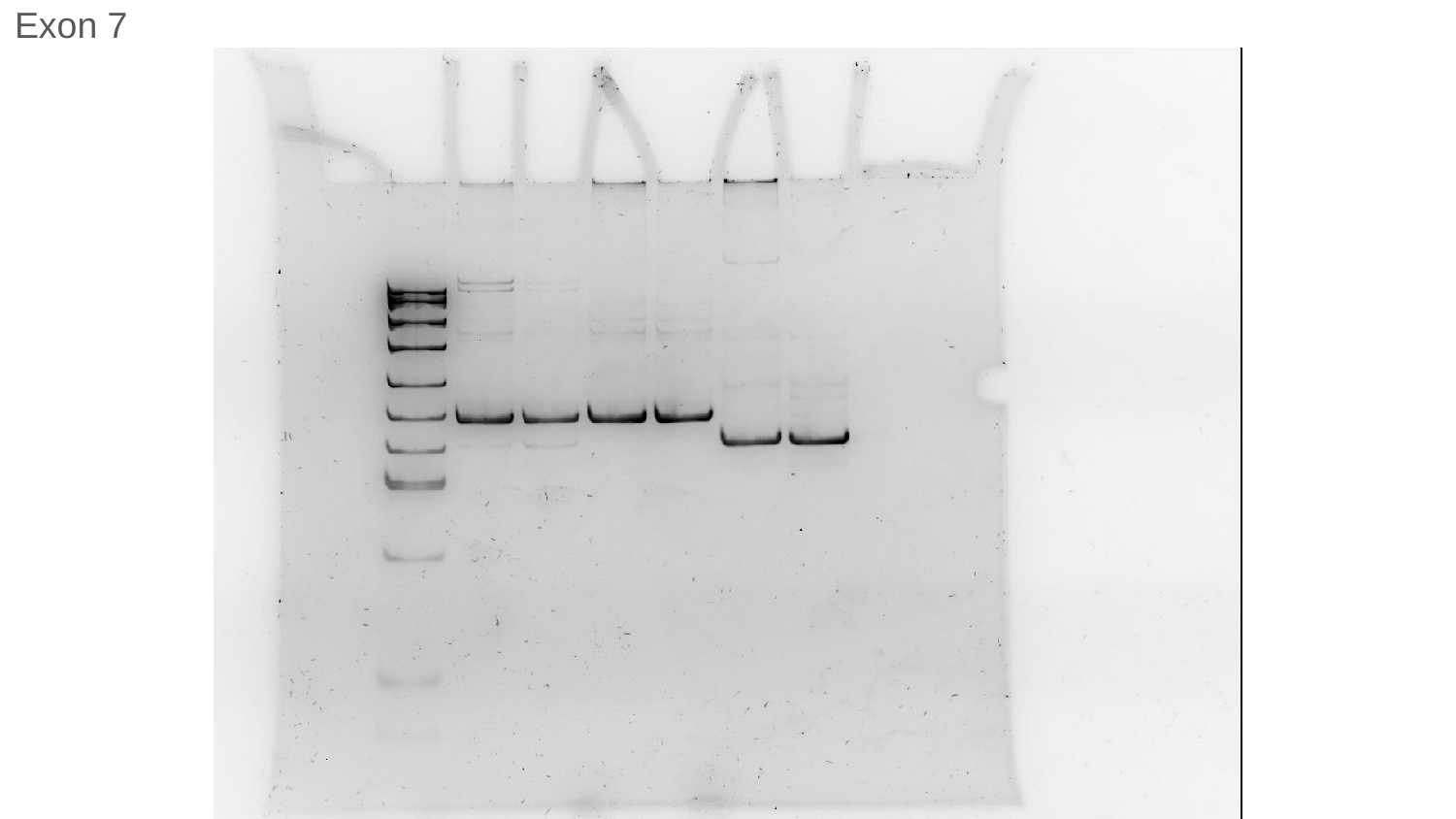

Exon 7

## Slide 6
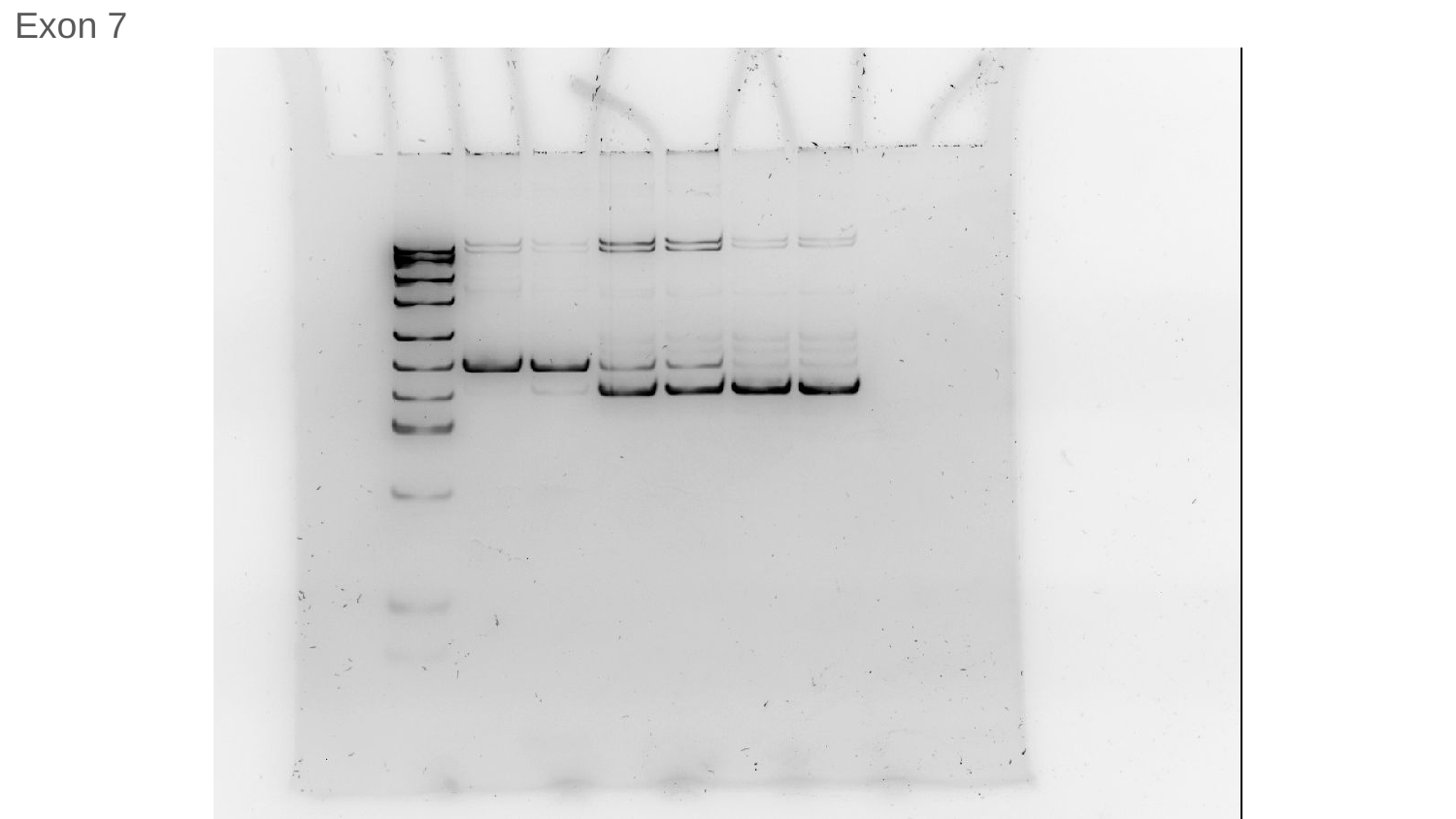

Exon 7

## Slide 7
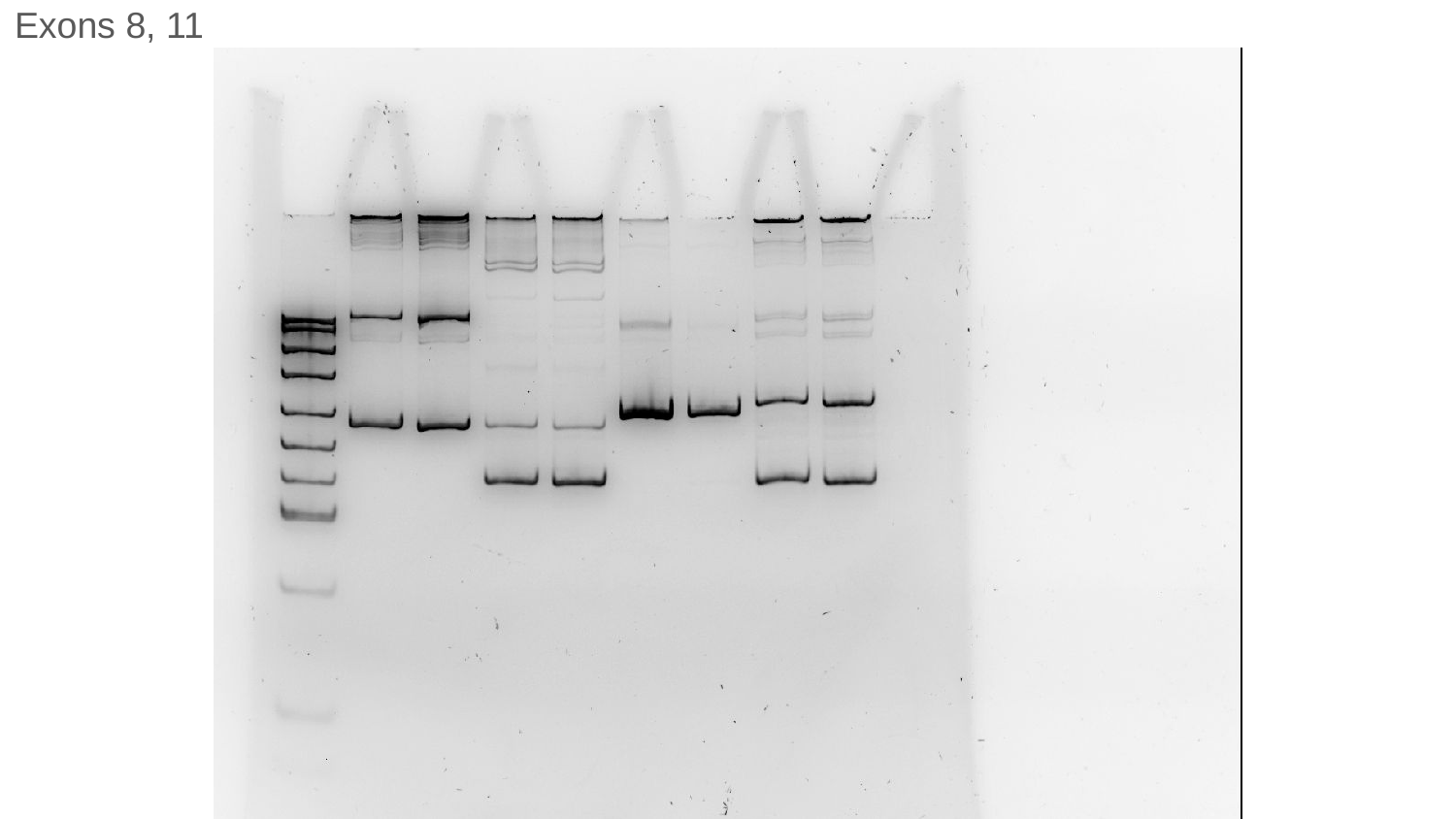

Exons 8, 11

## Slide 8
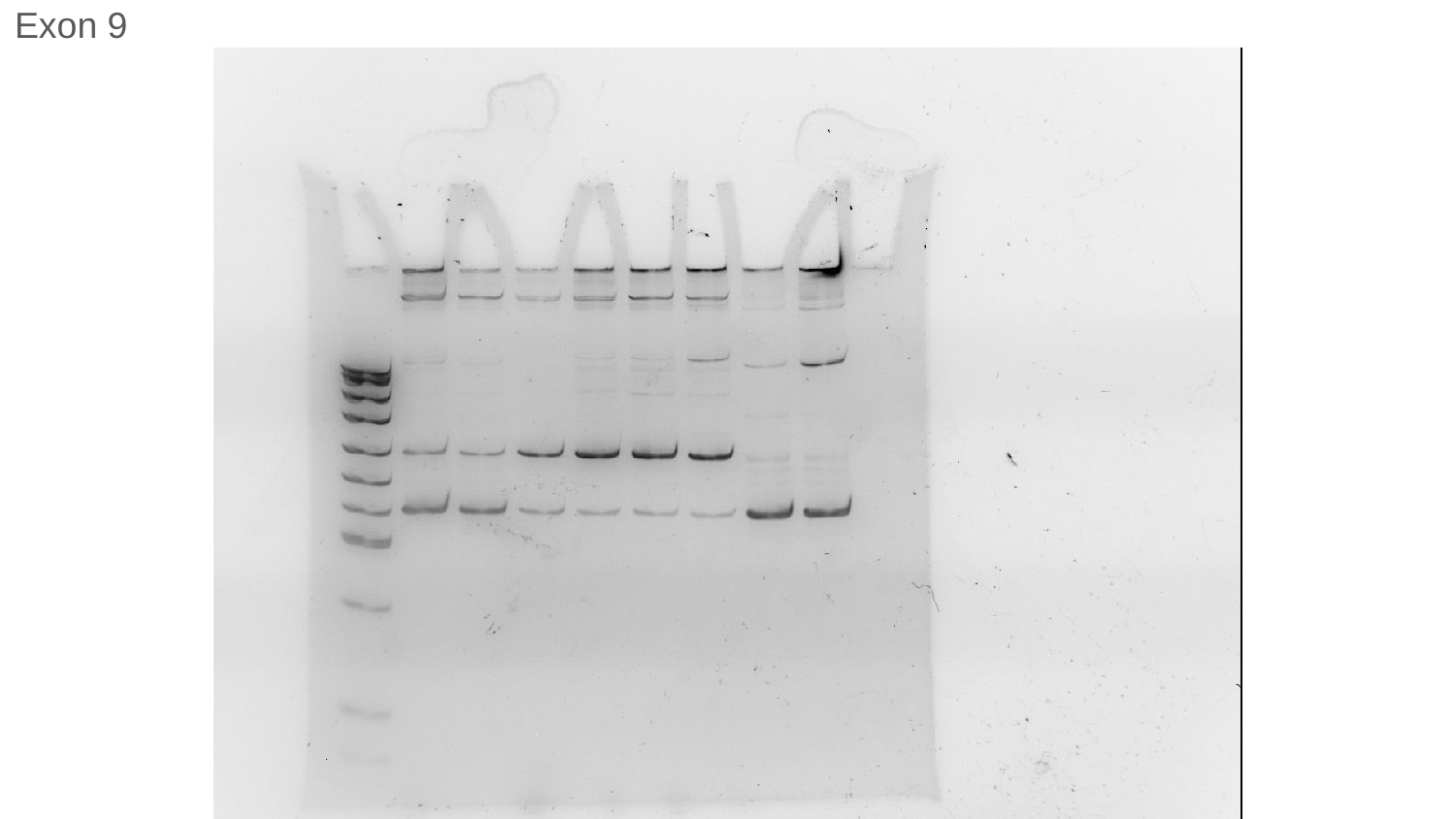

Exon 9

## Slide 9
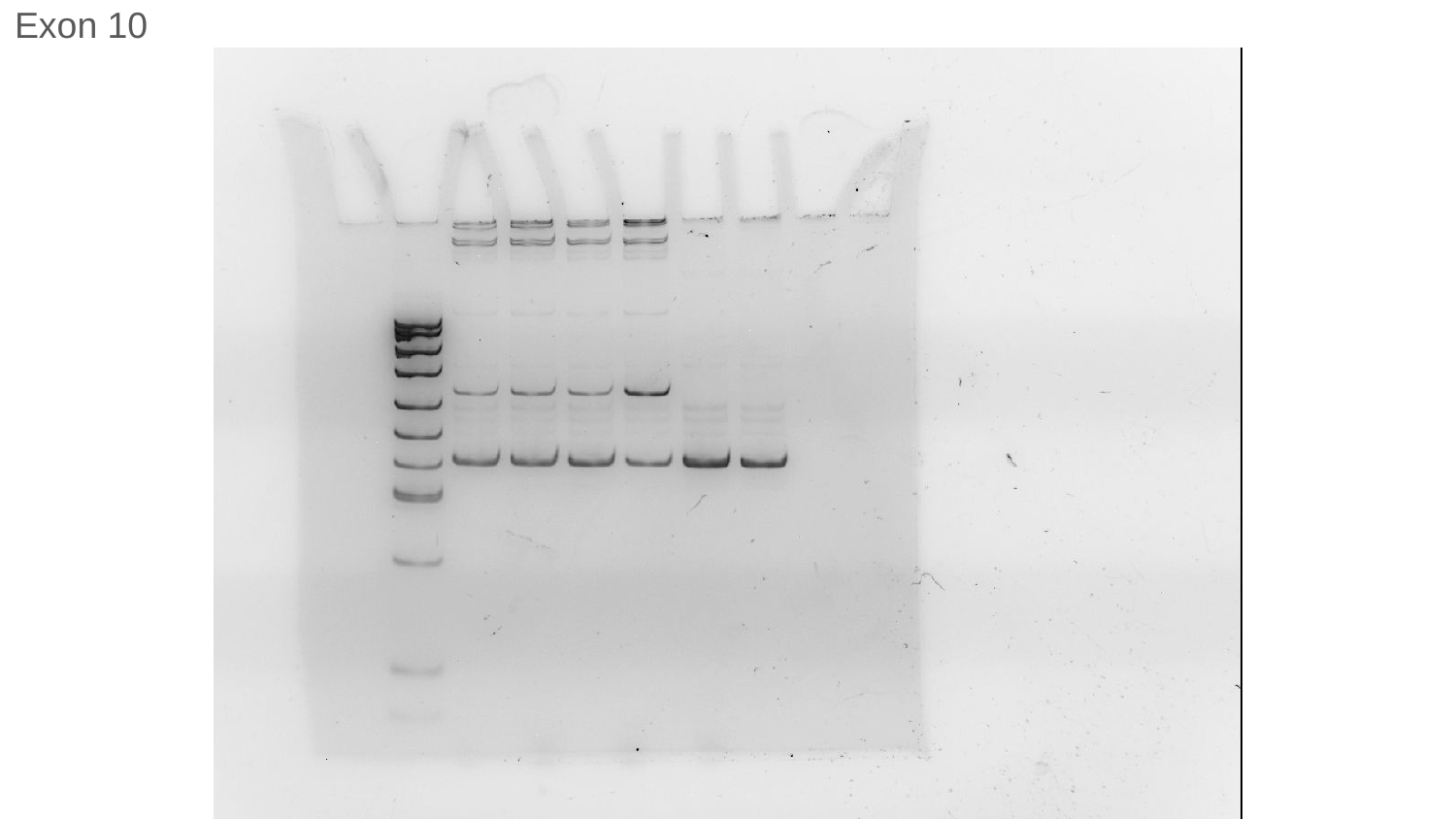

Exon 10

## Slide 10
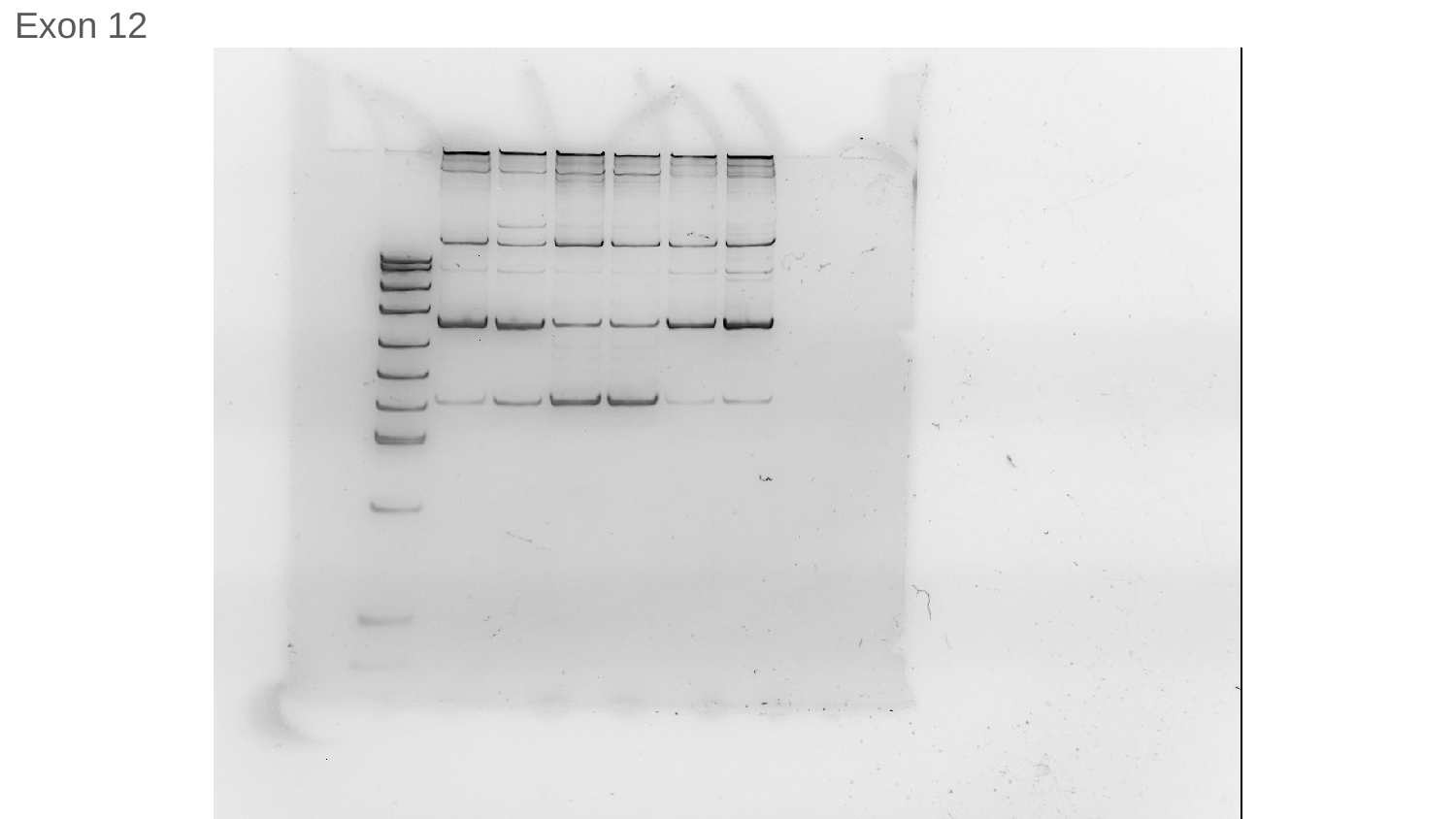

Exon 12

## Slide 11
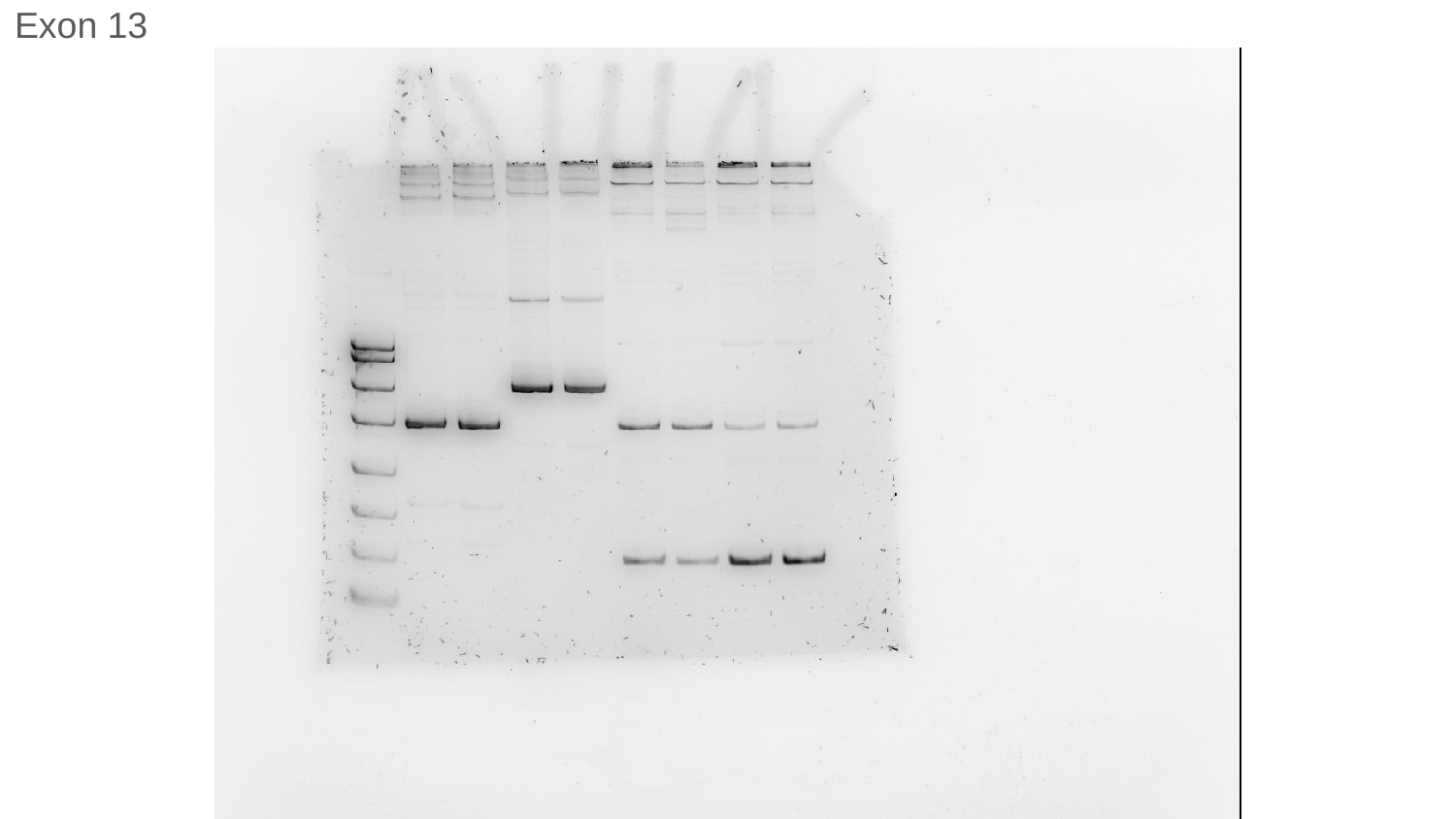

Exon 13

## Slide 12
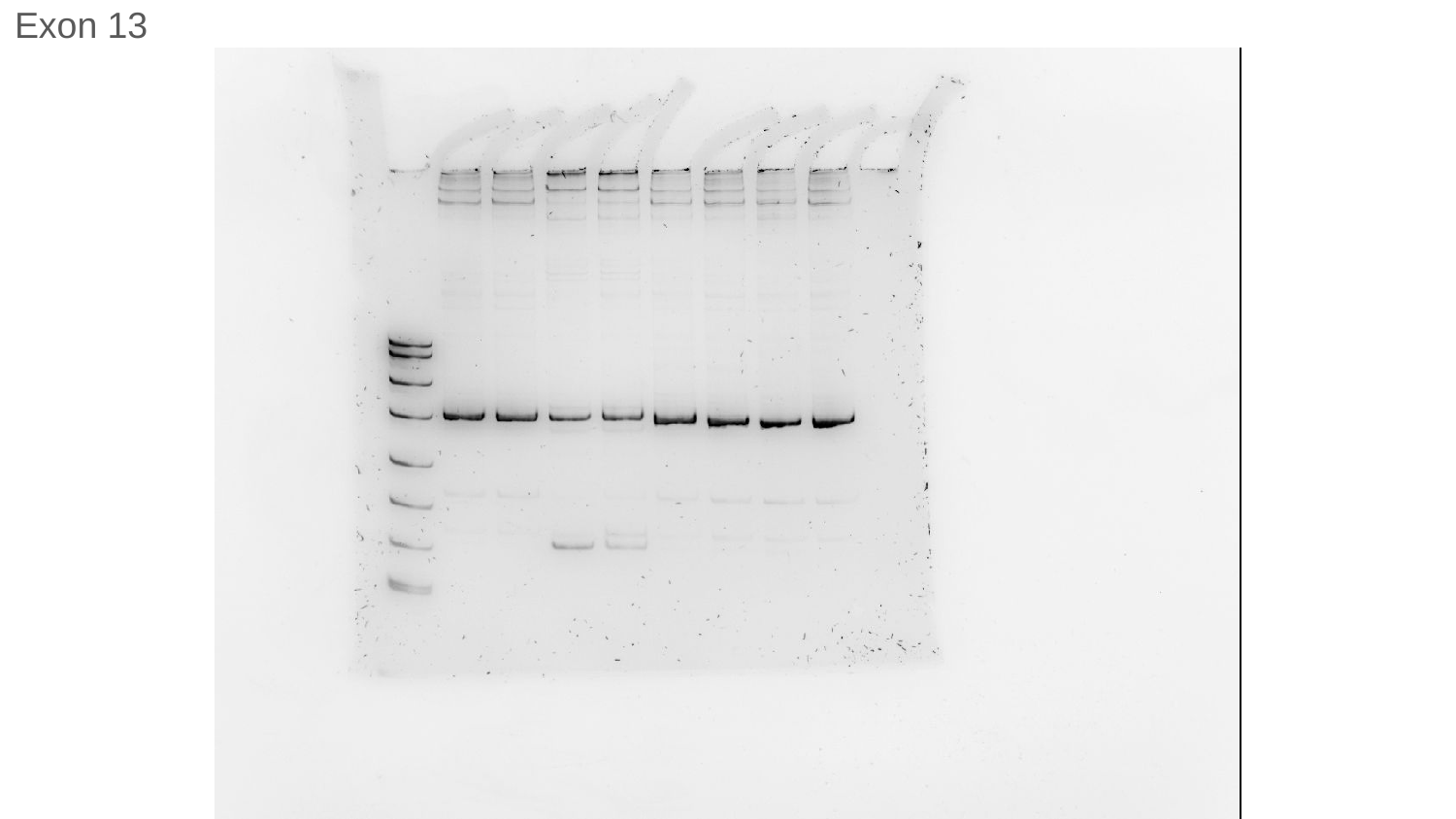

Exon 13

## Slide 13
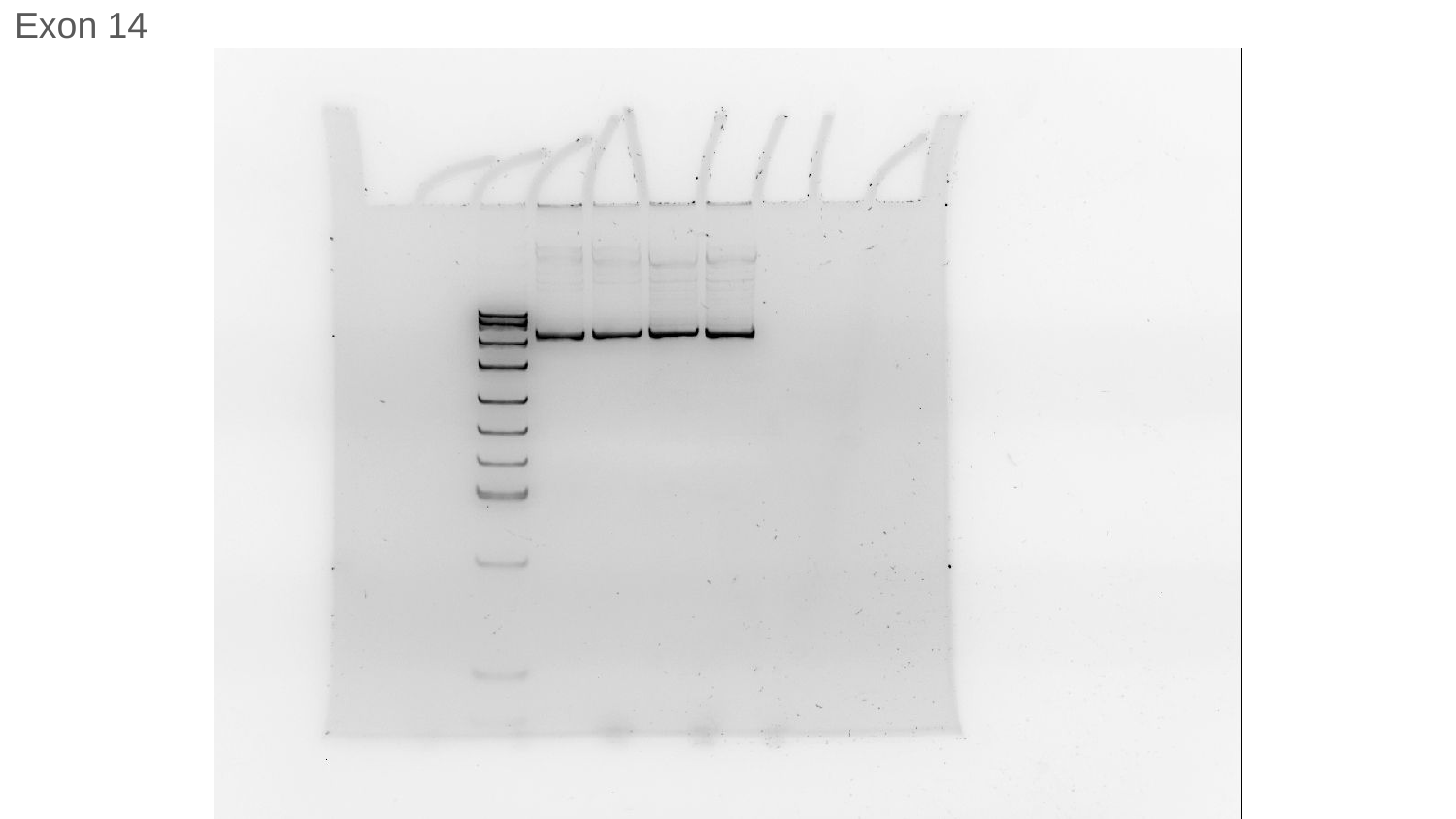

Exon 14
