# Supplementary material for: Functional Characterization of HGD Gene Variants by Minigene Splicing Assay
Source: Int J Mol Sci. 2025 Oct 31;26(21):10639. doi: 10.3390/ijms262110639 (PMC12608343; doi:10.3390/ijms262110639)
Supplement: Supplementary file 1 [file ijms-26-10639-s001.zip › Supplementary/Supplementary S4 (Sanger sequences).pptx]

## Slide 1
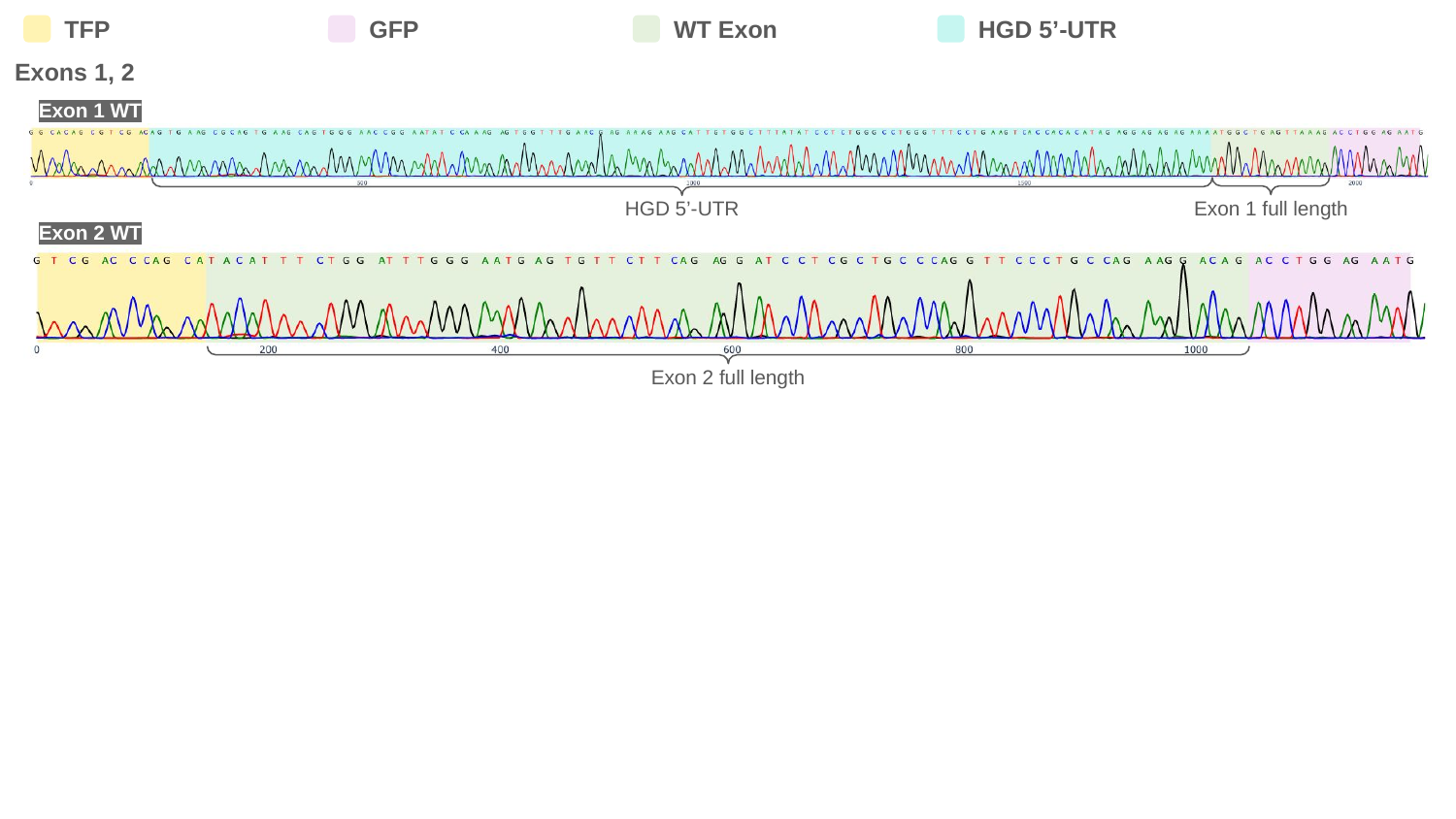

TFP
GFP
WT Exon
HGD 5’-UTR
Exons 1, 2
Exon 1 WT
HGD 5’-UTR
Exon 1 full length
Exon 2 WT
Exon 2 full length

## Slide 2
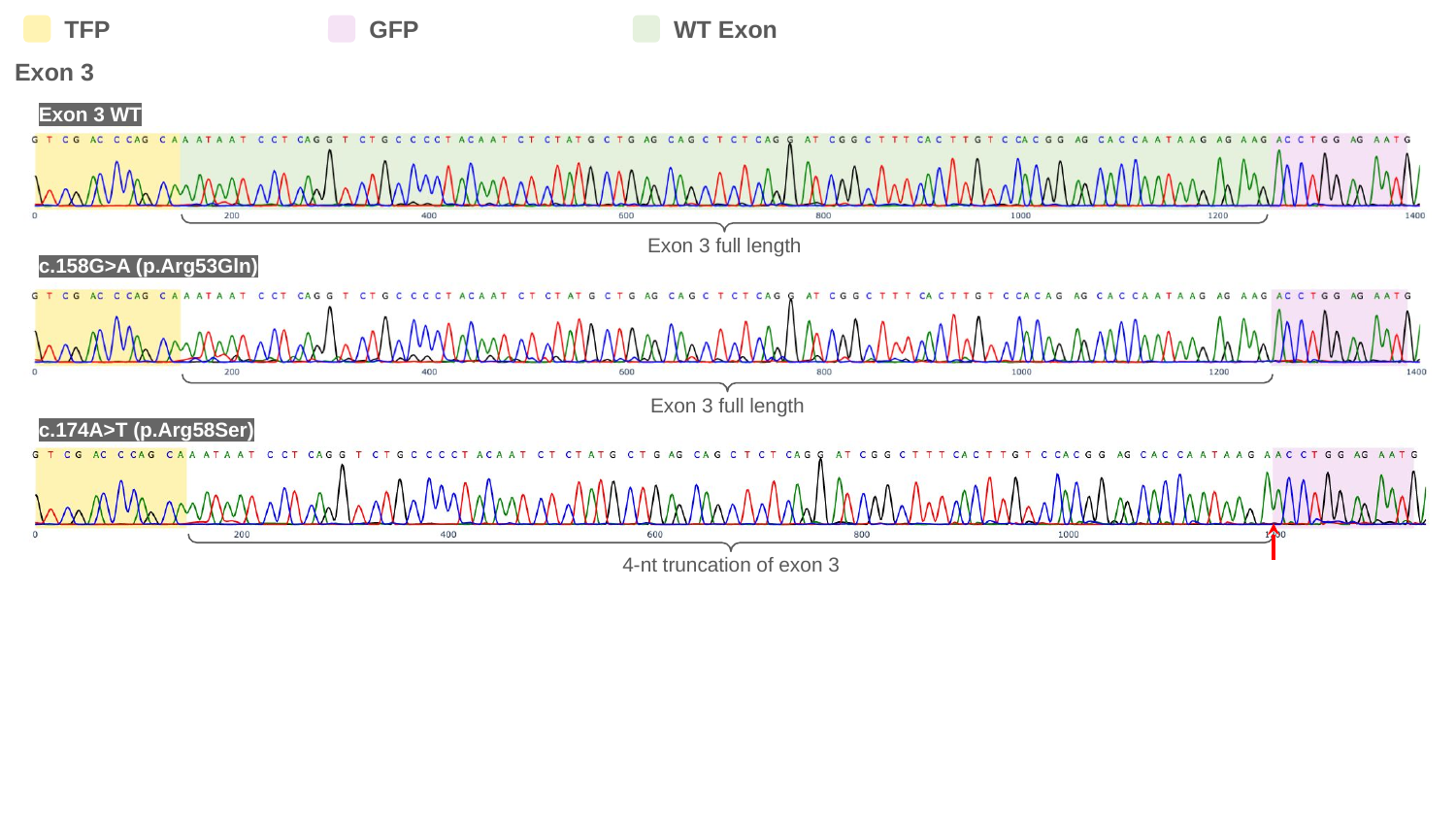

TFP
GFP
WT Exon
Exon 3
Exon 3 WT
Exon 3 full length
c.158G>A (p.Arg53Gln)
Exon 3 full length
c.174A>T (p.Arg58Ser)
4-nt truncation of exon 3

## Slide 3
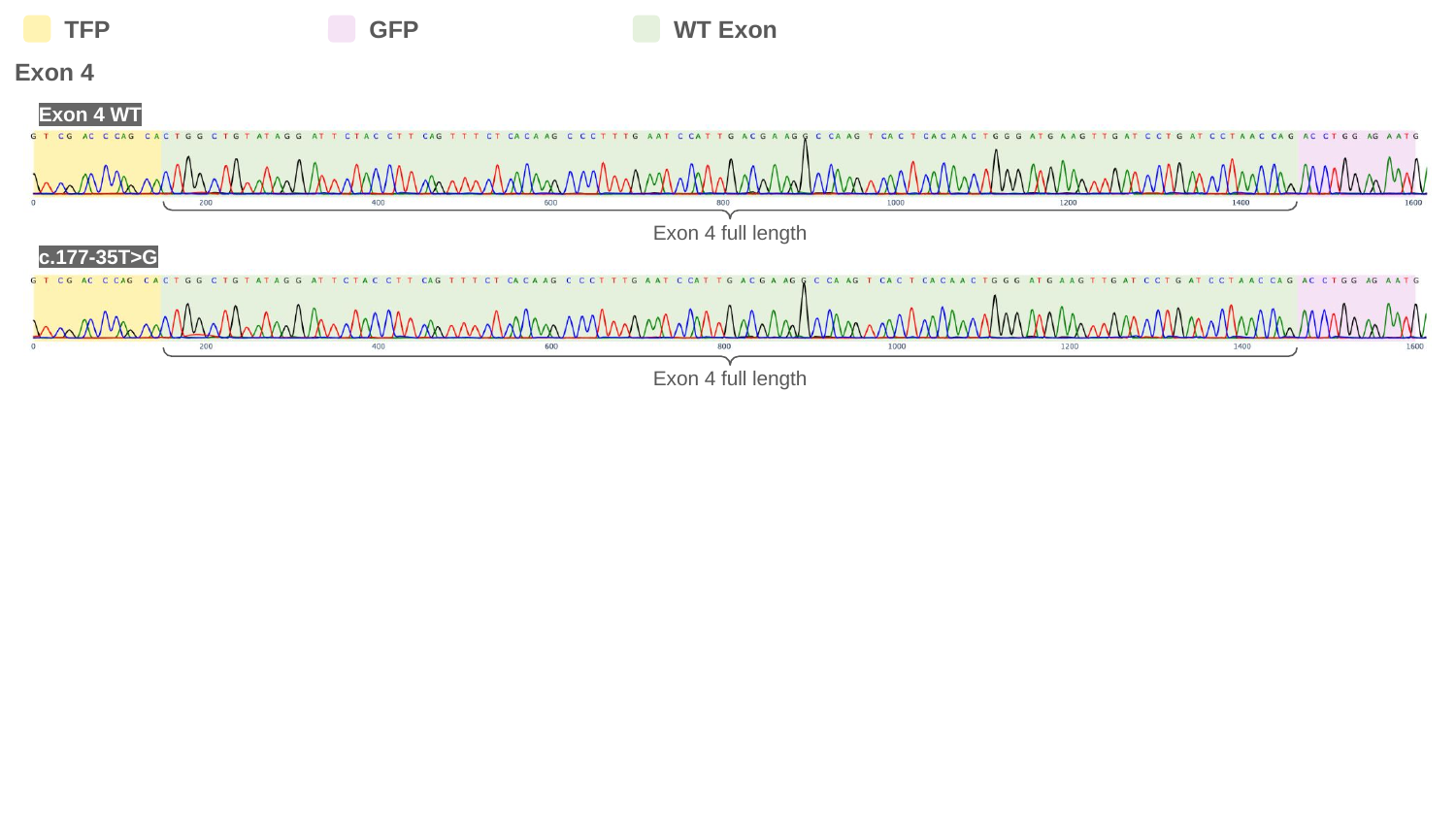

TFP
GFP
WT Exon
Exon 4
Exon 4 WT
Exon 4 full length
c.177-35T>G
Exon 4 full length

## Slide 4
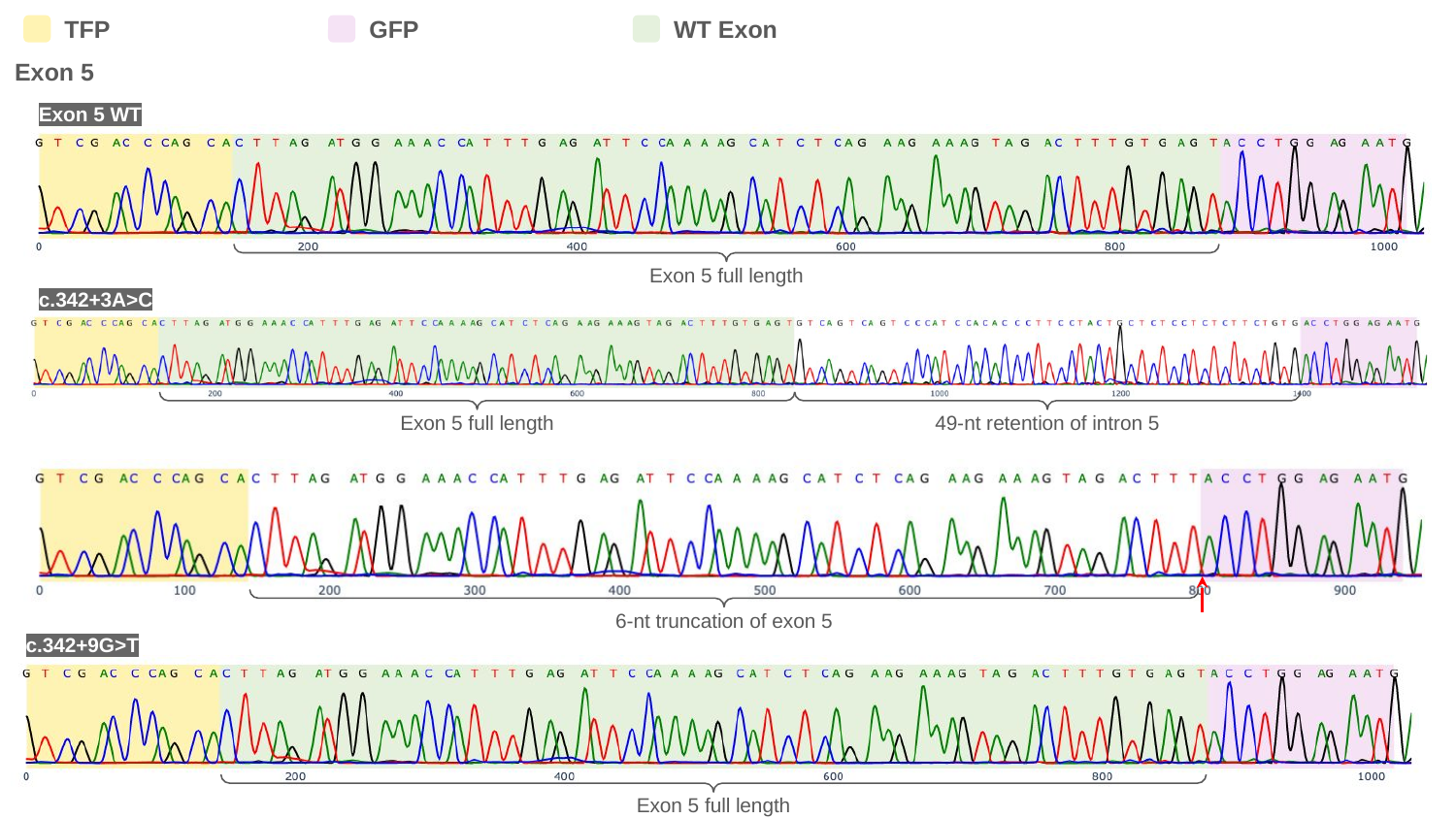

TFP
GFP
WT Exon
Exon 5
Exon 5 WT
Exon 5 full length
c.342+3A>C
Exon 5 full length
49-nt retention of intron 5
6-nt truncation of exon 5
c.342+9G>T
Exon 5 full length

## Slide 5
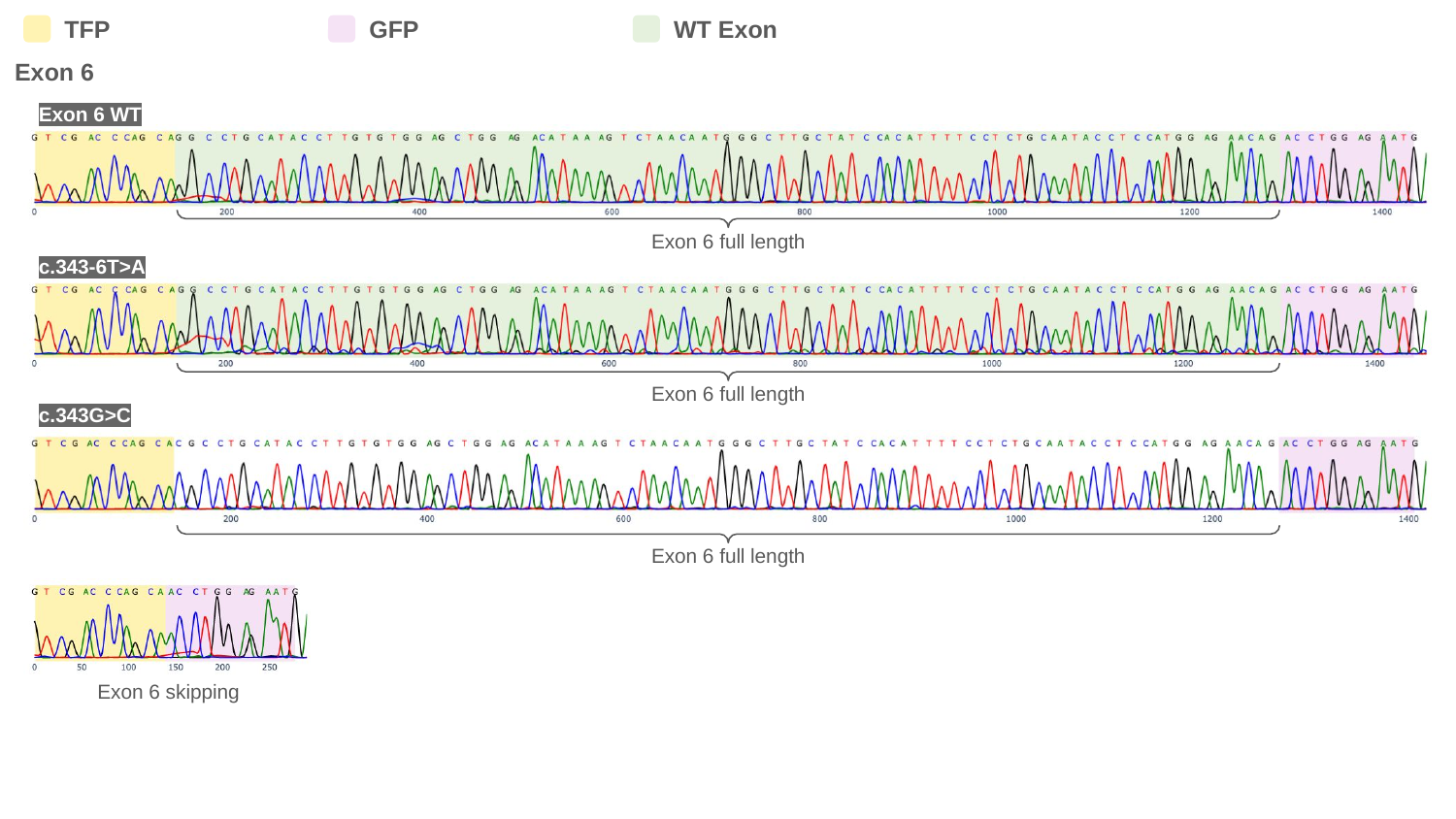

TFP
GFP
WT Exon
Exon 6
Exon 6 WT
Exon 6 full length
c.343-6T>A
Exon 6 full length
c.343G>C
Exon 6 full length
Exon 6 skipping

## Slide 6
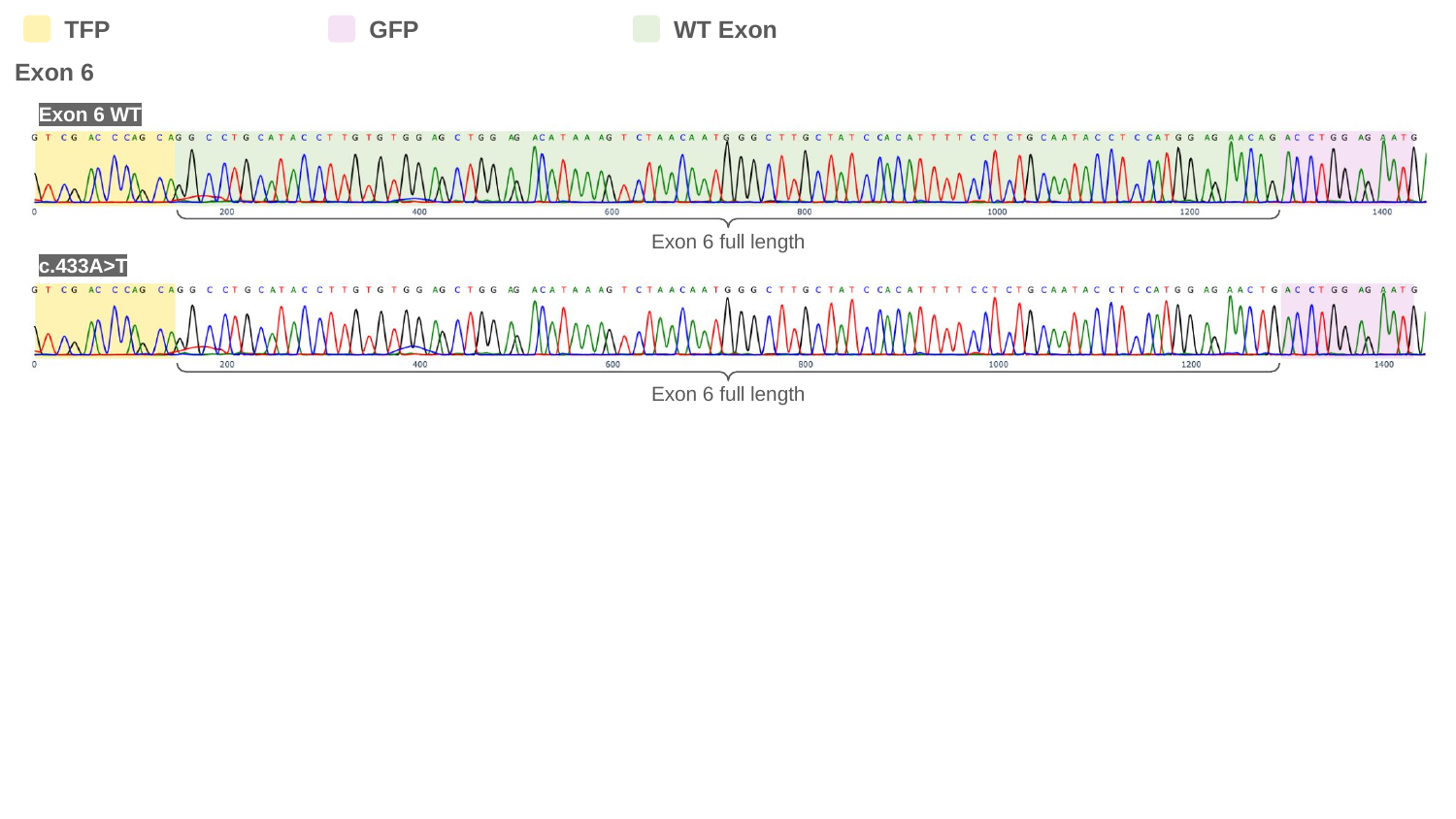

TFP
GFP
WT Exon
Exon 6
Exon 6 WT
Exon 6 full length
c.433A>T
Exon 6 full length

## Slide 7
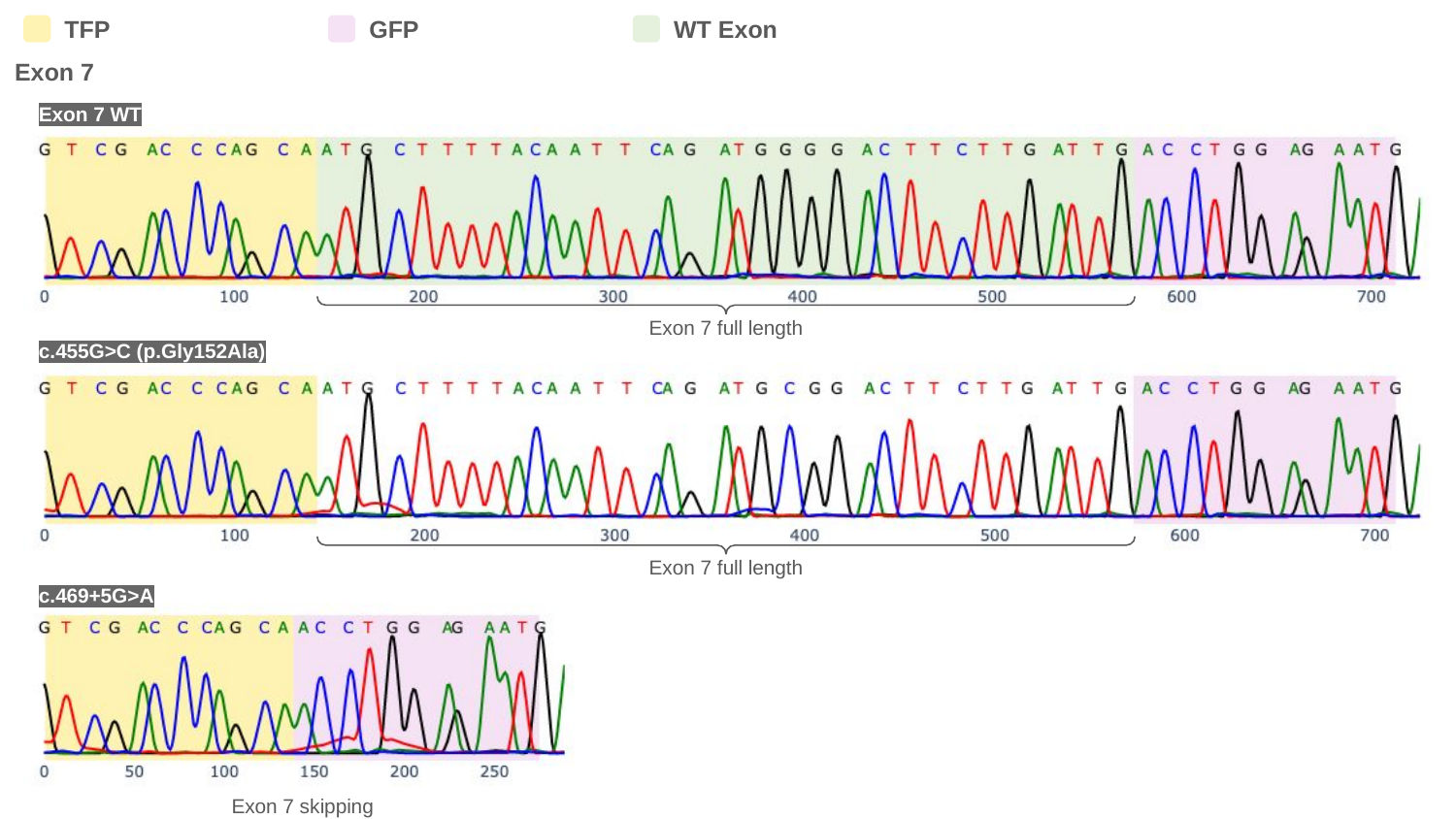

TFP
GFP
WT Exon
Exon 7
Exon 7 WT
Exon 7 full length
c.455G>C (p.Gly152Ala)
Exon 7 full length
c.469+5G>A
Exon 7 skipping

## Slide 8
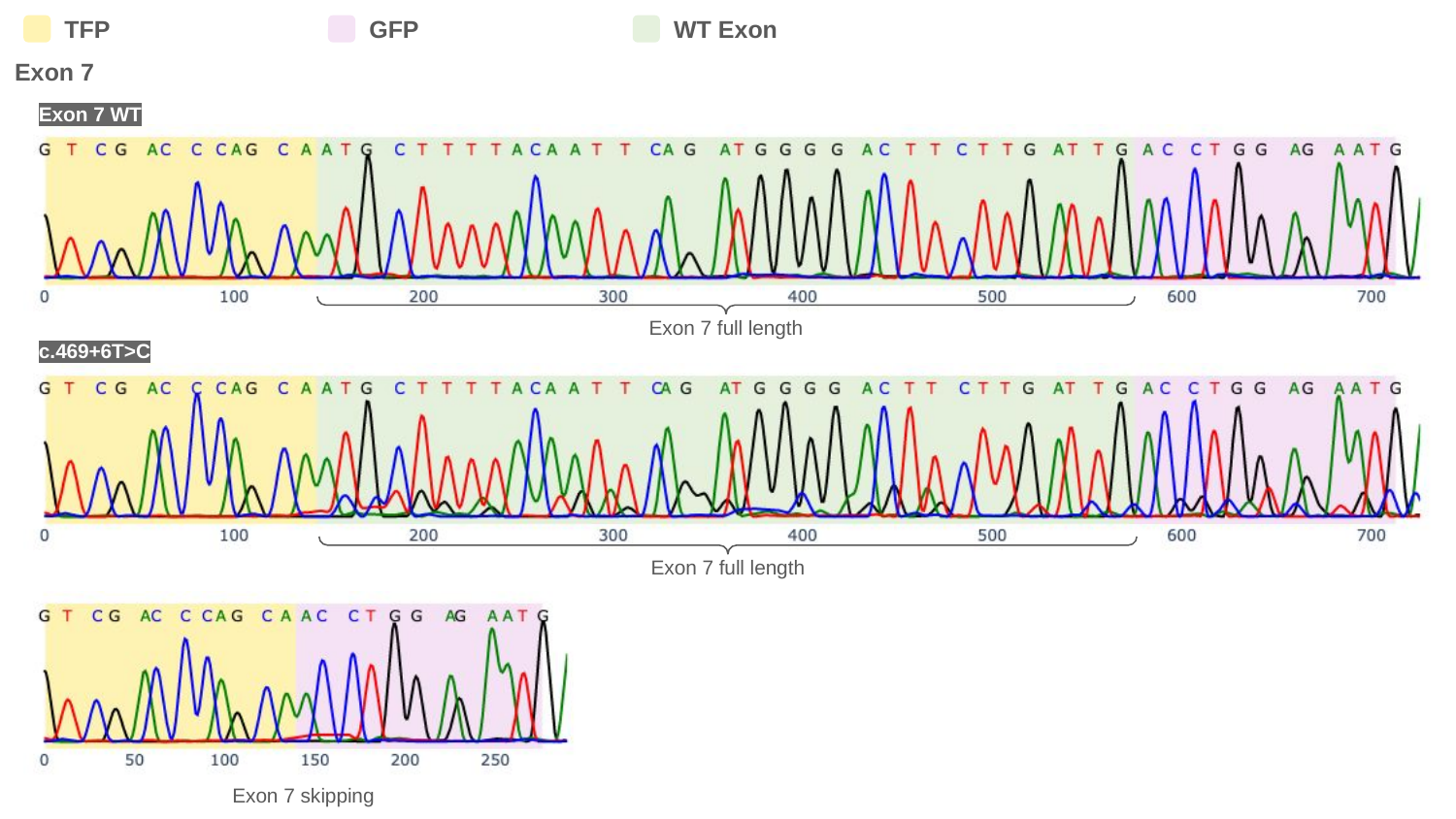

TFP
GFP
WT Exon
Exon 7
Exon 7 WT
Exon 7 full length
c.469+6T>C
Exon 7 full length
Exon 7 skipping

## Slide 9
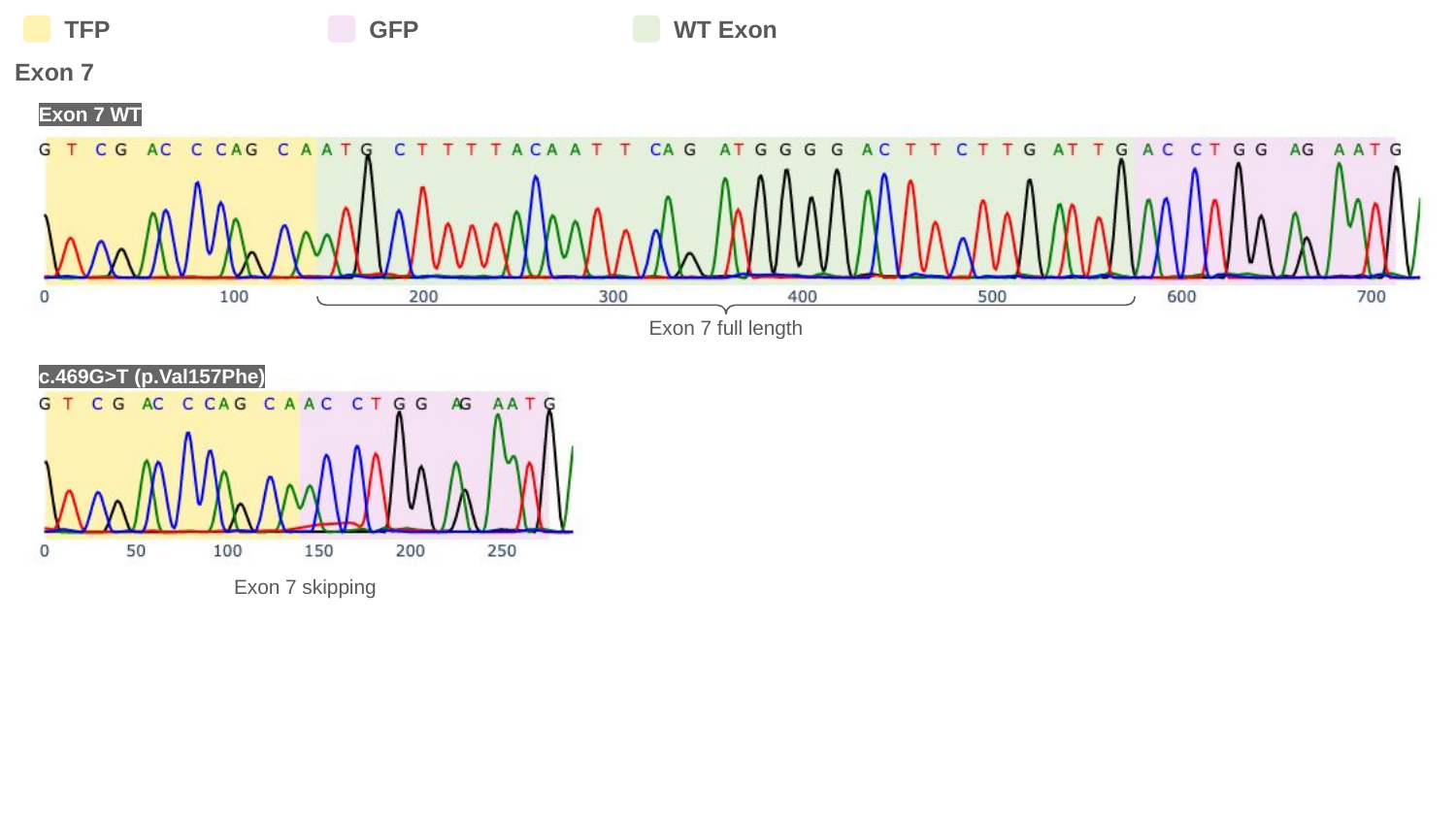

TFP
GFP
WT Exon
Exon 7
Exon 7 WT
Exon 7 full length
c.469G>T (p.Val157Phe)
Exon 7 skipping

## Slide 10
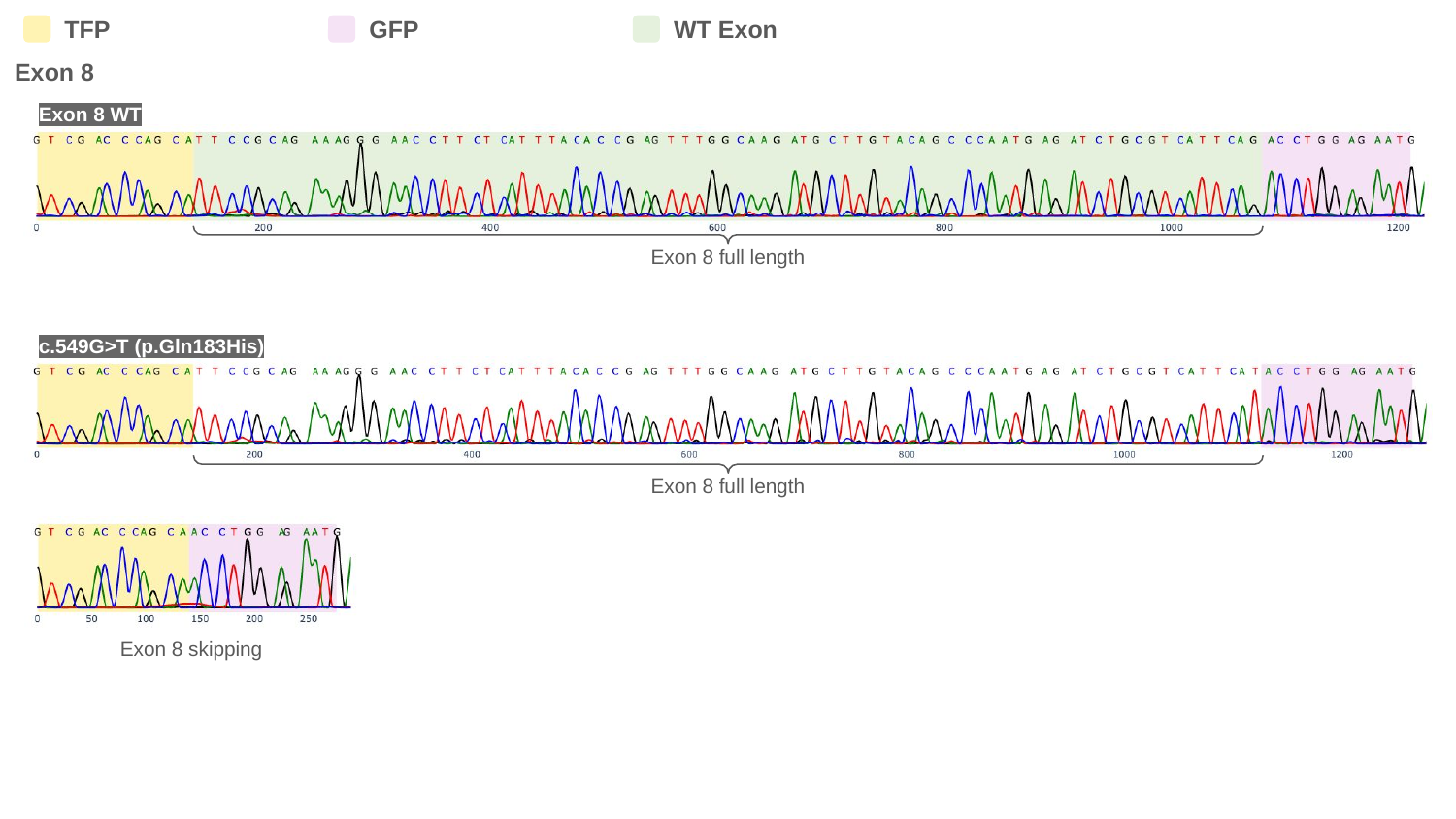

TFP
GFP
WT Exon
Exon 8
Exon 8 WT
Exon 8 full length
c.549G>T (p.Gln183His)
Exon 8 full length
Exon 8 skipping

## Slide 11
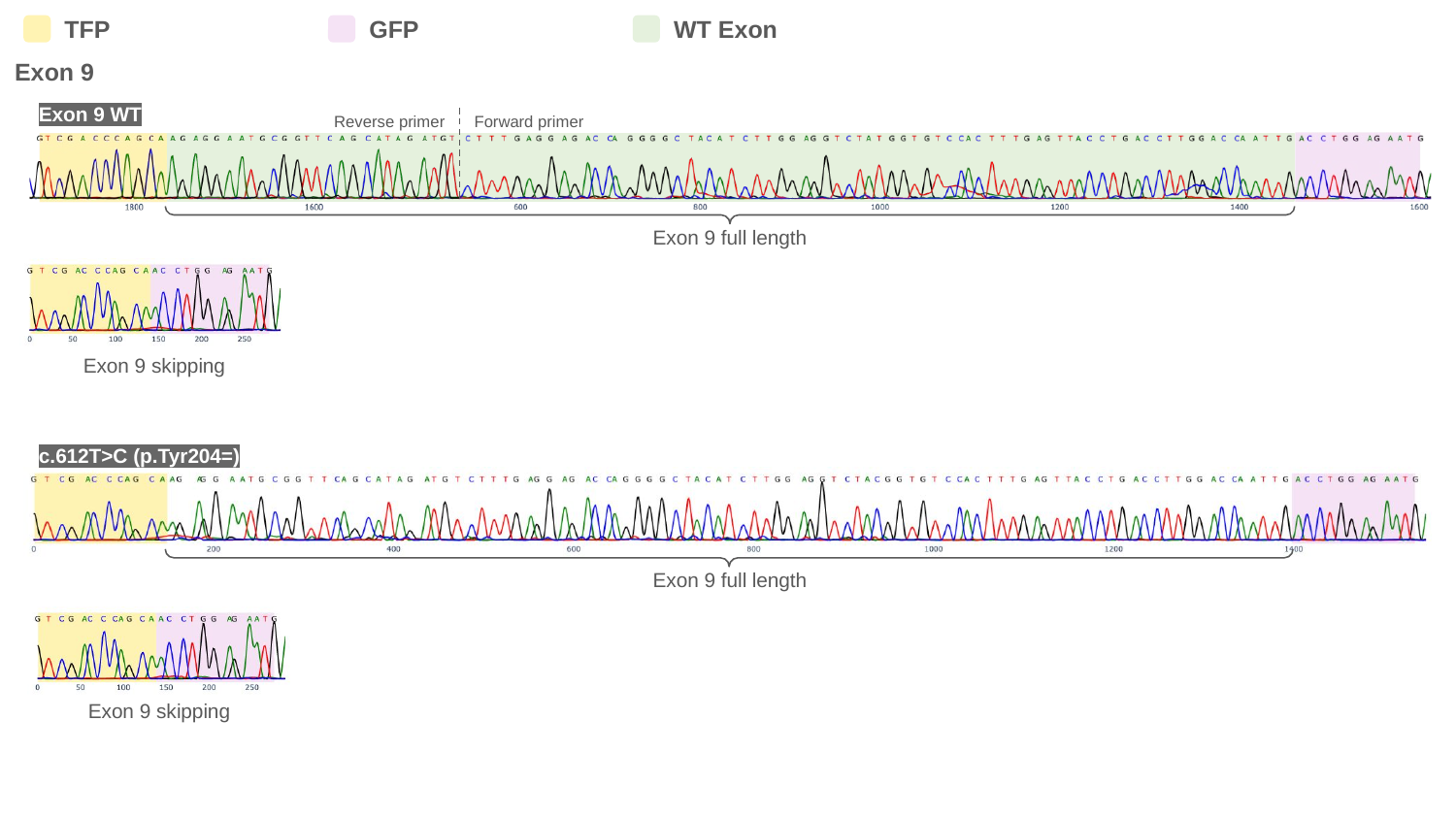

TFP
GFP
WT Exon
Exon 9
Exon 9 WT
Reverse primer
Forward primer
Exon 9 full length
Exon 9 skipping
c.612T>C (p.Tyr204=)
Exon 9 full length
Exon 9 skipping

## Slide 12
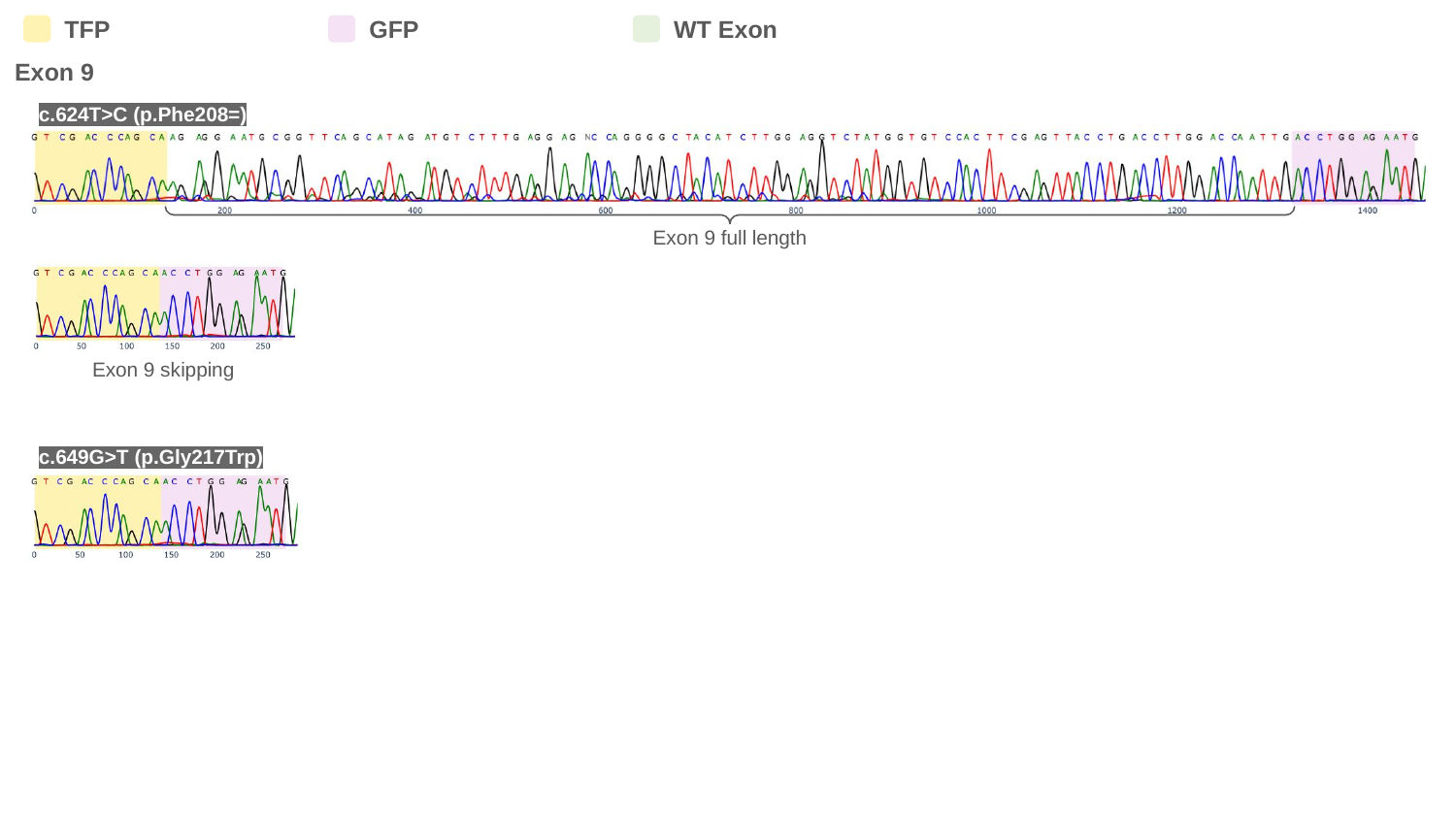

TFP
GFP
WT Exon
Exon 9
c.624T>C (p.Phe208=)
Exon 9 full length
Exon 9 skipping
c.649G>T (p.Gly217Trp)

## Slide 13
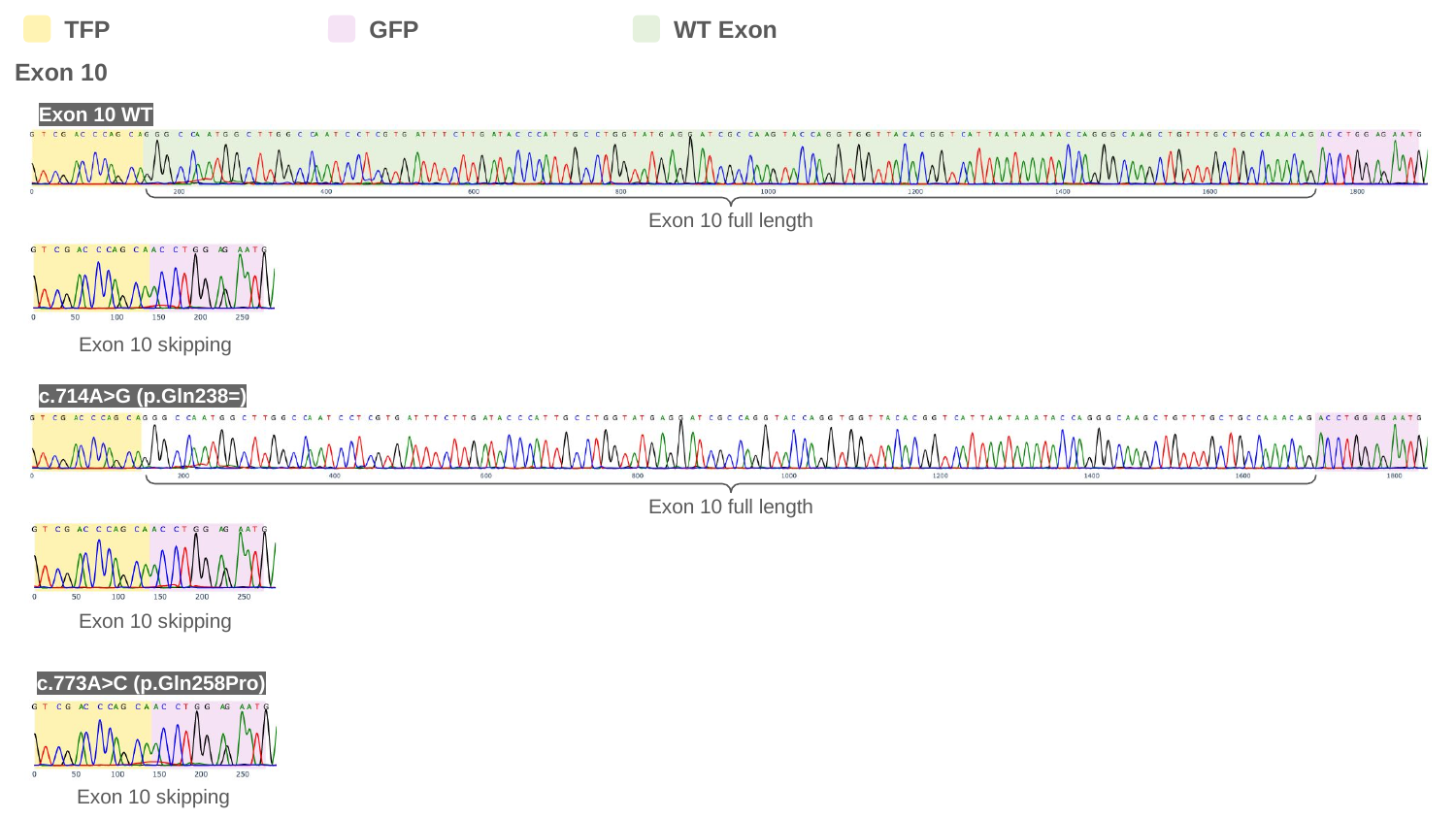

TFP
GFP
WT Exon
Exon 10
Exon 10 WT
Exon 10 full length
Exon 10 skipping
c.714A>G (p.Gln238=)
Exon 10 full length
Exon 10 skipping
c.773A>C (p.Gln258Pro)
Exon 10 skipping

## Slide 14
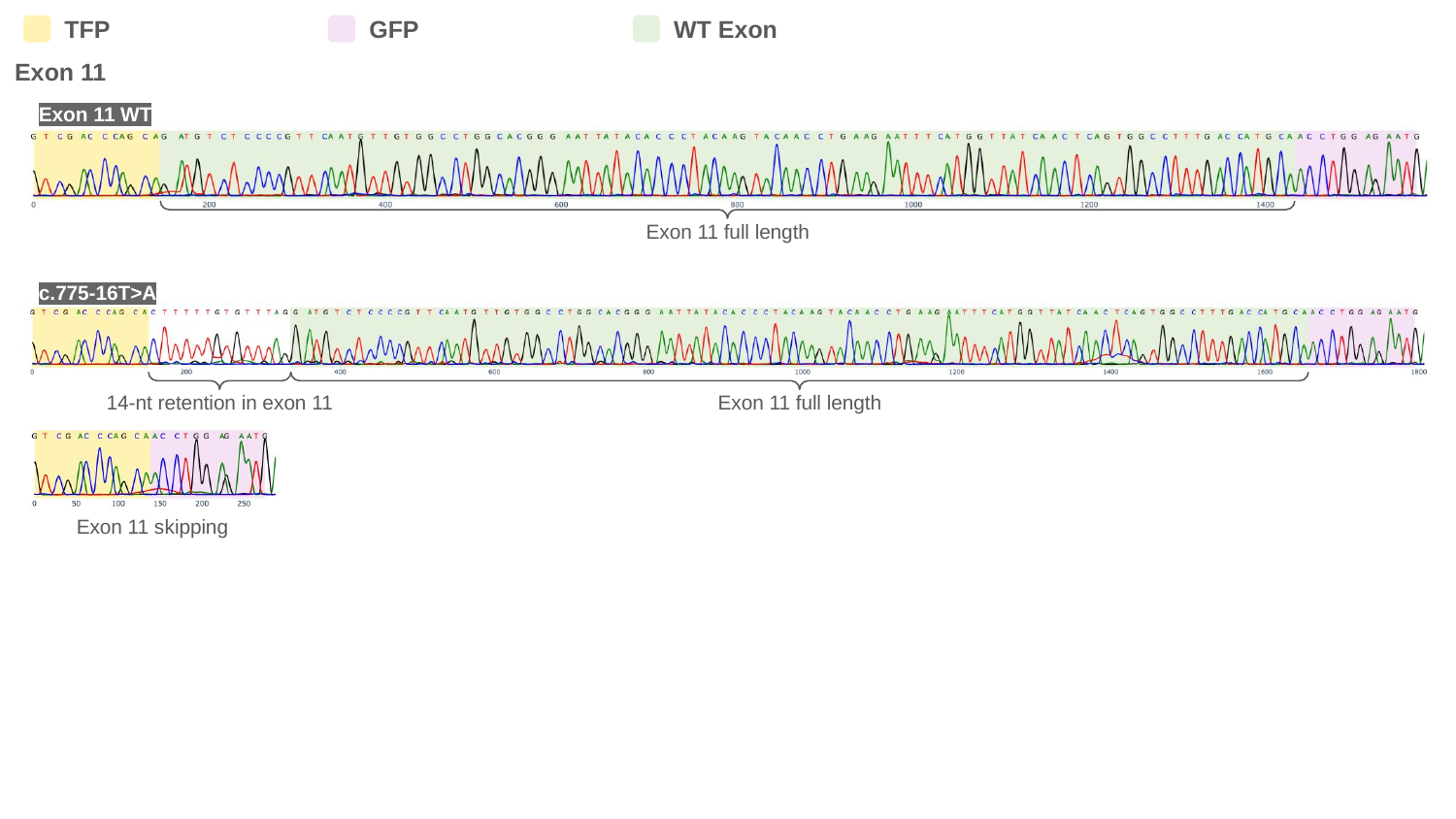

TFP
GFP
WT Exon
Exon 11
Exon 11 WT
Exon 11 full length
c.775-16T>A
14-nt retention in exon 11
Exon 11 full length
Exon 11 skipping

## Slide 15
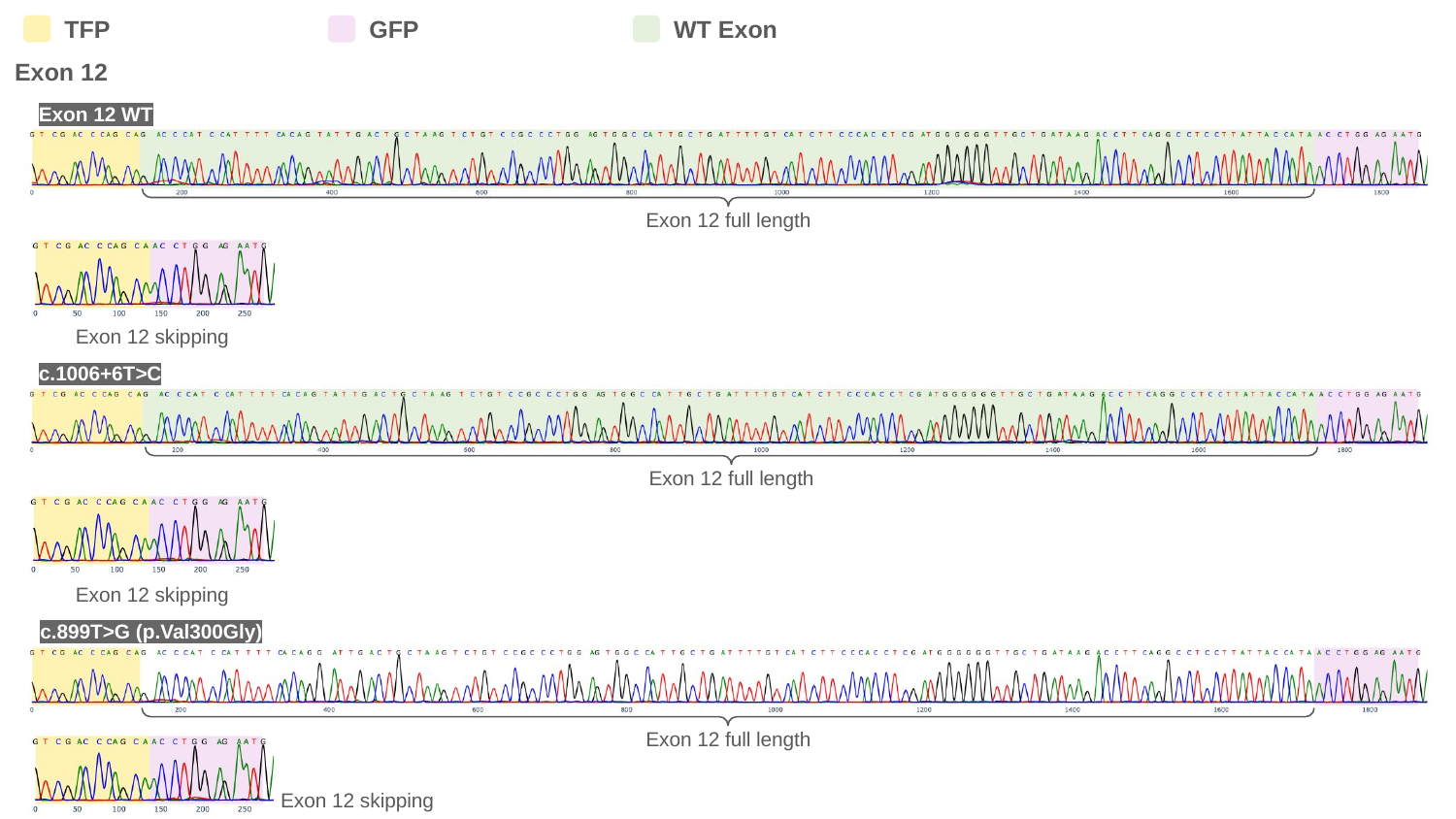

TFP
GFP
WT Exon
Exon 12
Exon 12 WT
Exon 12 full length
Exon 12 skipping
c.1006+6T>C
Exon 12 full length
Exon 12 skipping
c.899T>G (p.Val300Gly)
Exon 12 full length
Exon 12 skipping

## Slide 16
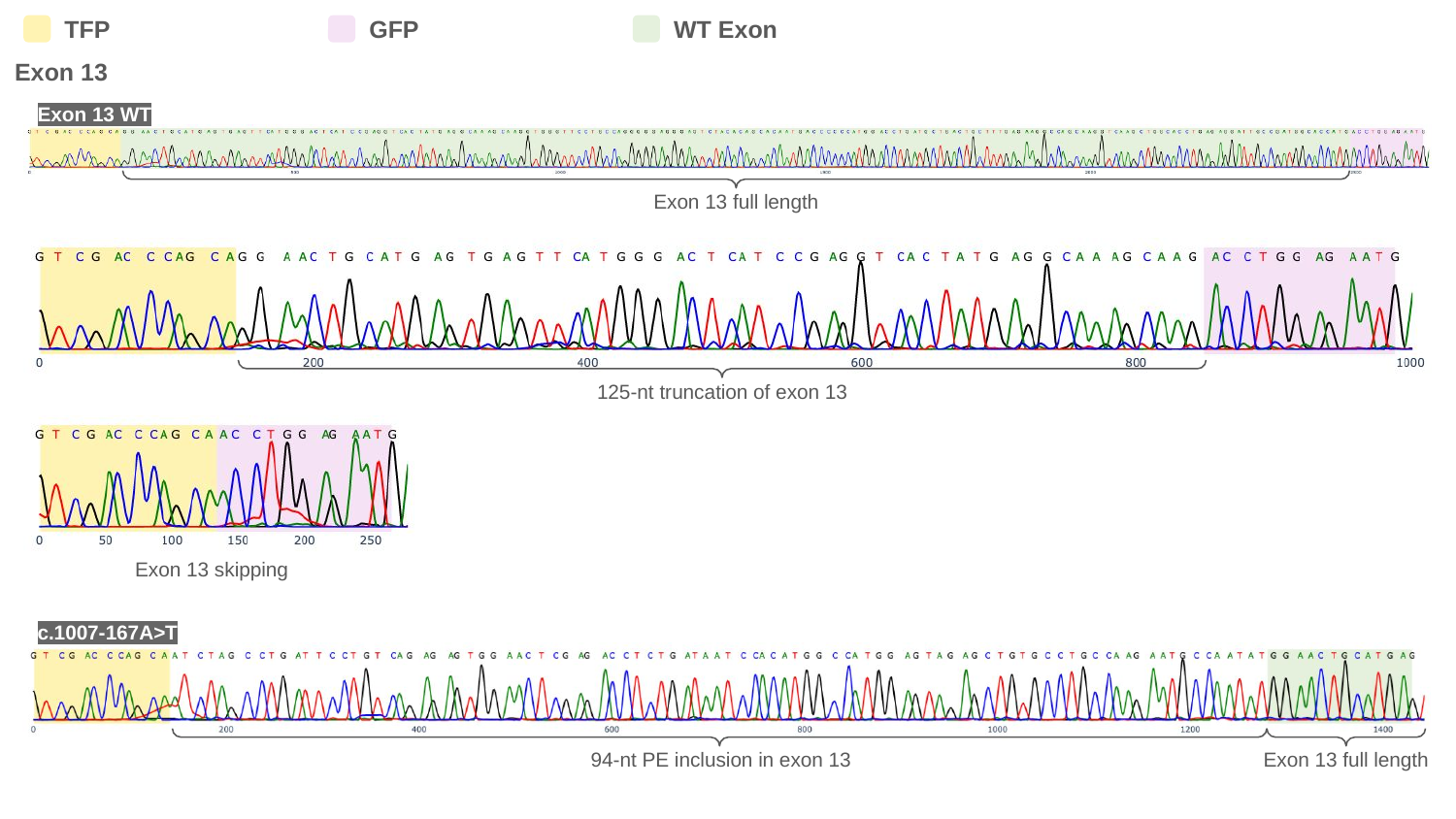

TFP
GFP
WT Exon
Exon 13
Exon 13 WT
Exon 13 full length
125-nt truncation of exon 13
Exon 13 skipping
c.1007-167A>T
94-nt PE inclusion in exon 13
Exon 13 full length

## Slide 17
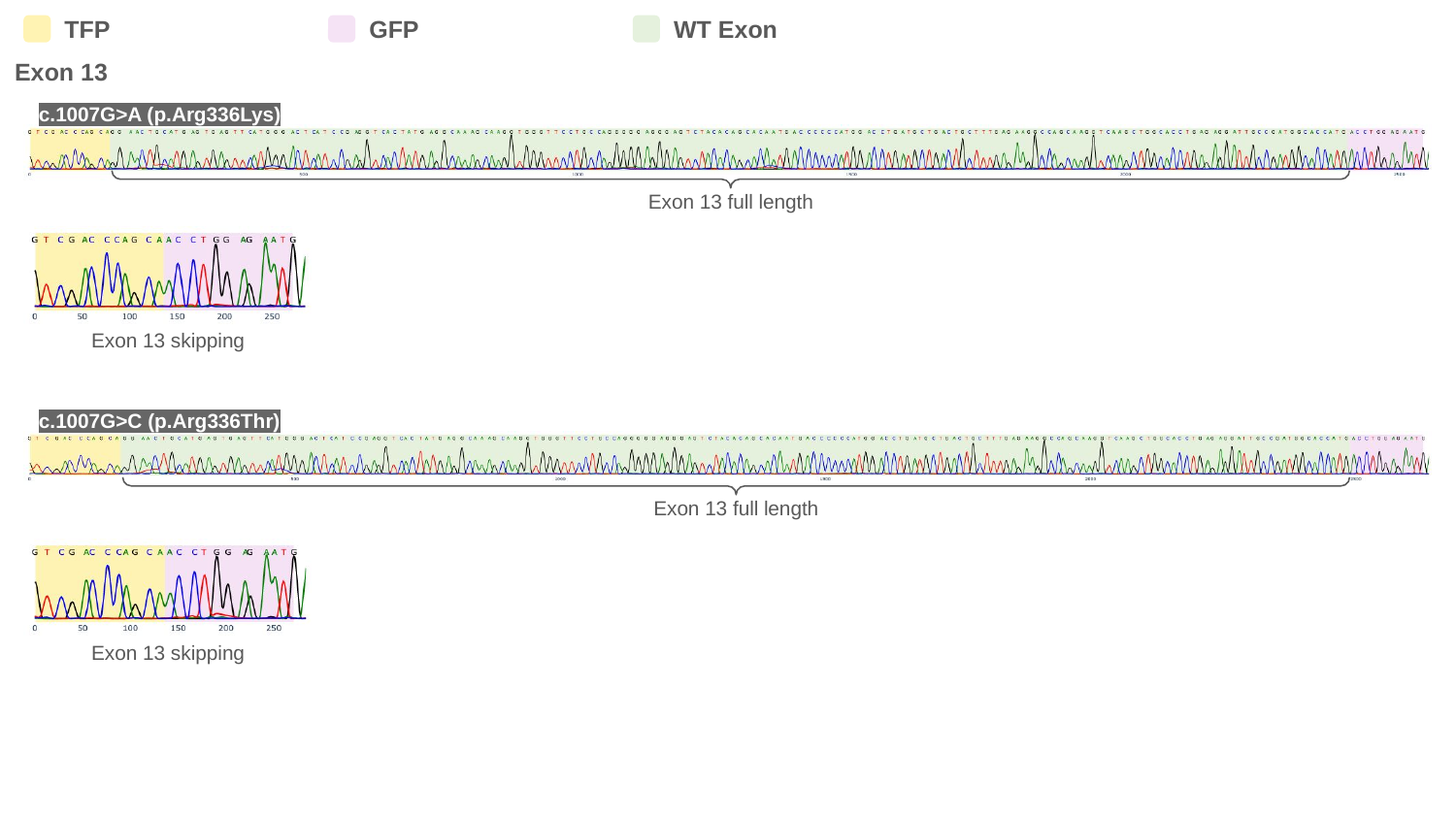

TFP
GFP
WT Exon
Exon 13
c.1007G>A (p.Arg336Lys)
Exon 13 full length
Exon 13 skipping
c.1007G>C (p.Arg336Thr)
Exon 13 full length
Exon 13 skipping

## Slide 18
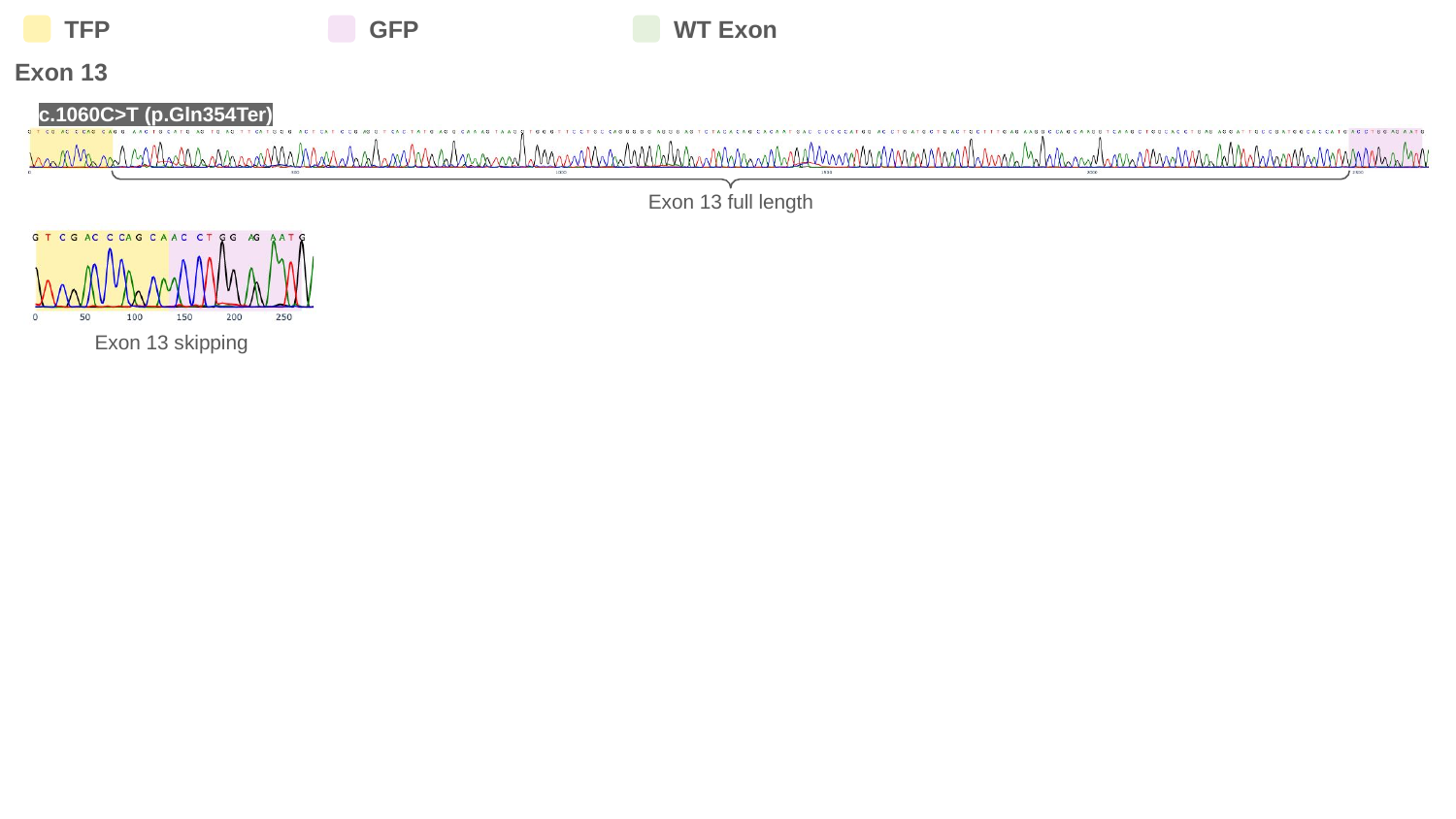

TFP
GFP
WT Exon
Exon 13
c.1060C>T (p.Gln354Ter)
Exon 13 full length
Exon 13 skipping

## Slide 19
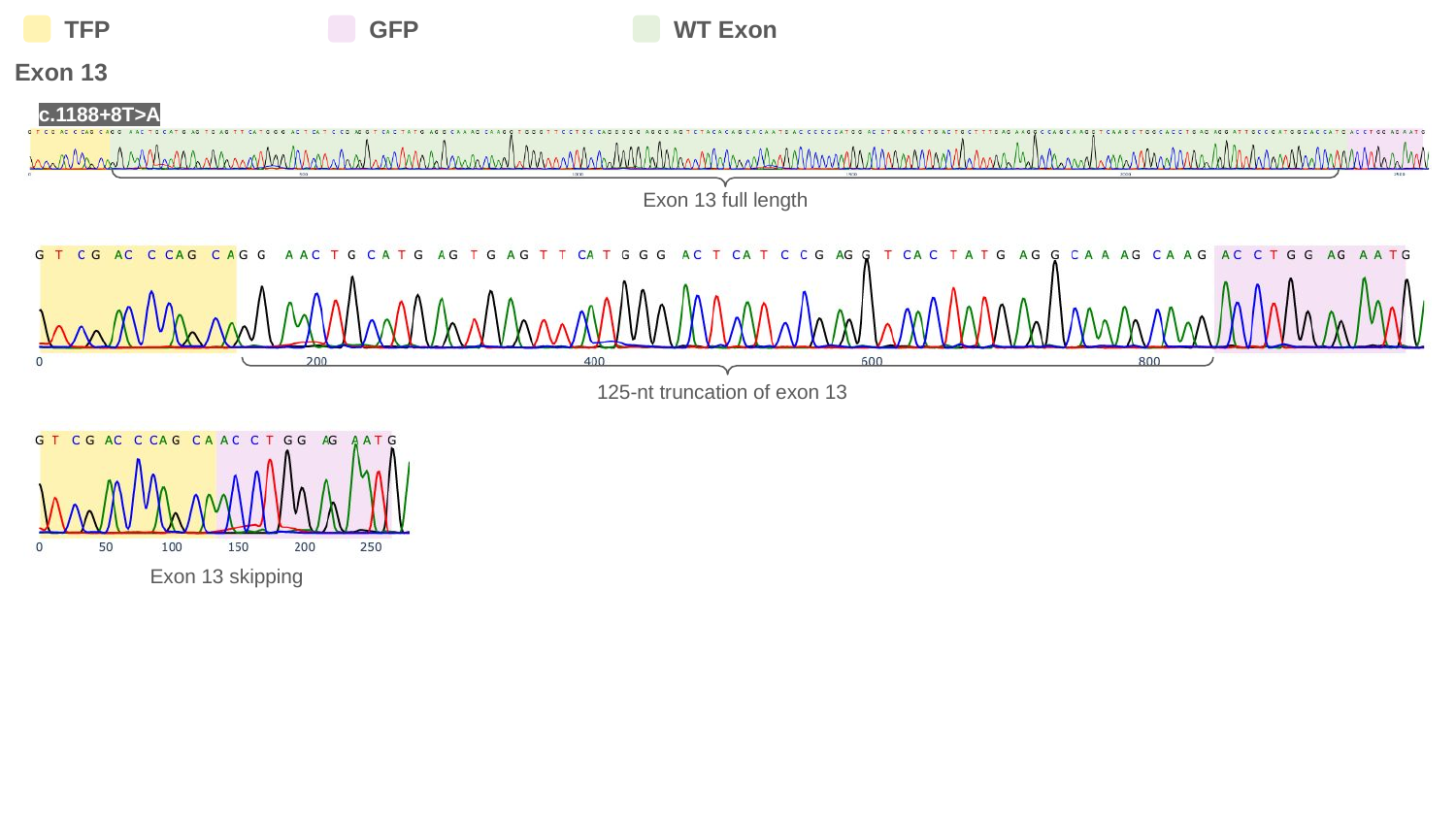

TFP
GFP
WT Exon
Exon 13
c.1188+8T>A
Exon 13 full length
125-nt truncation of exon 13
Exon 13 skipping

## Slide 20
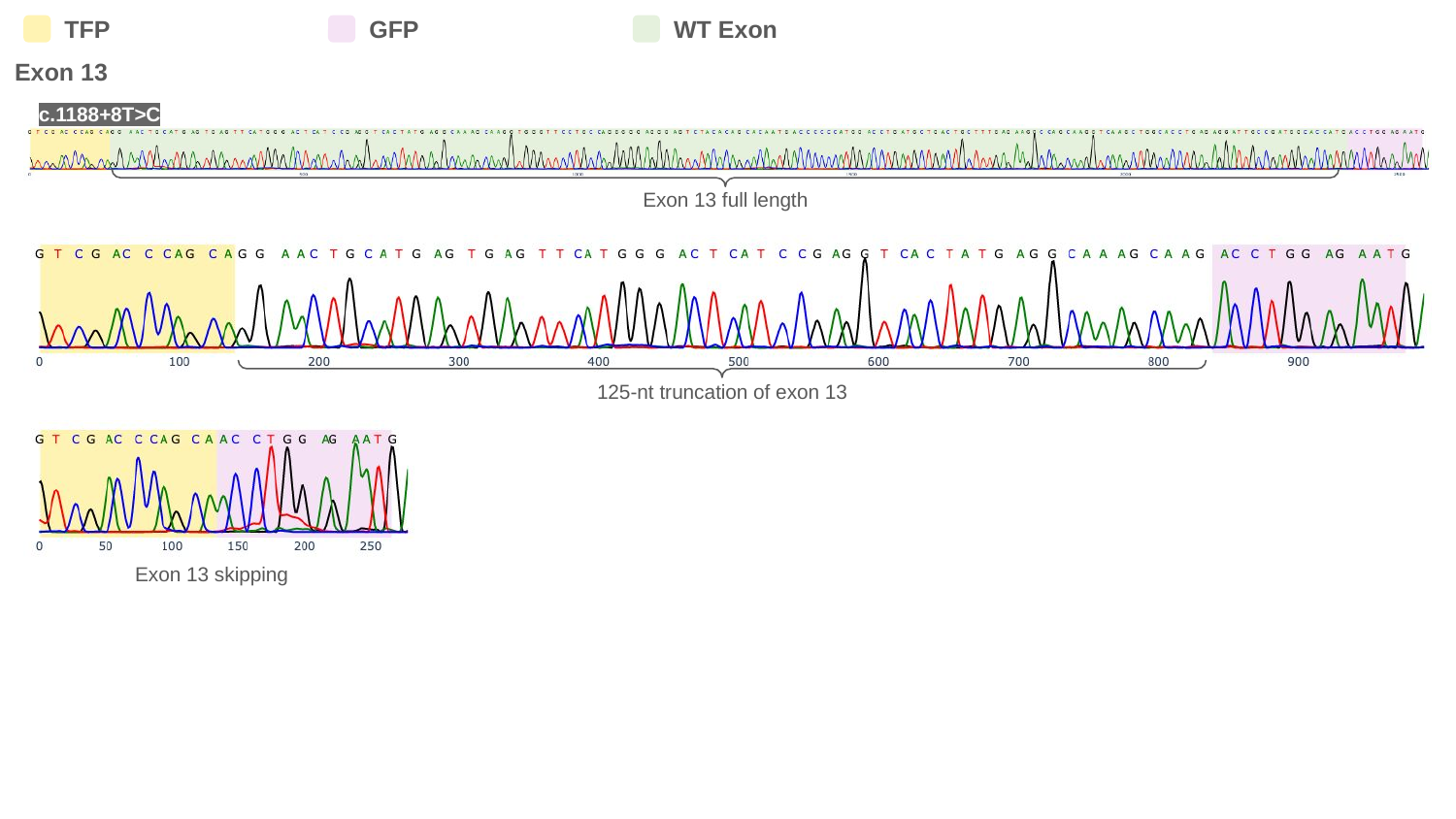

TFP
GFP
WT Exon
Exon 13
c.1188+8T>C
Exon 13 full length
125-nt truncation of exon 13
Exon 13 skipping

## Slide 21
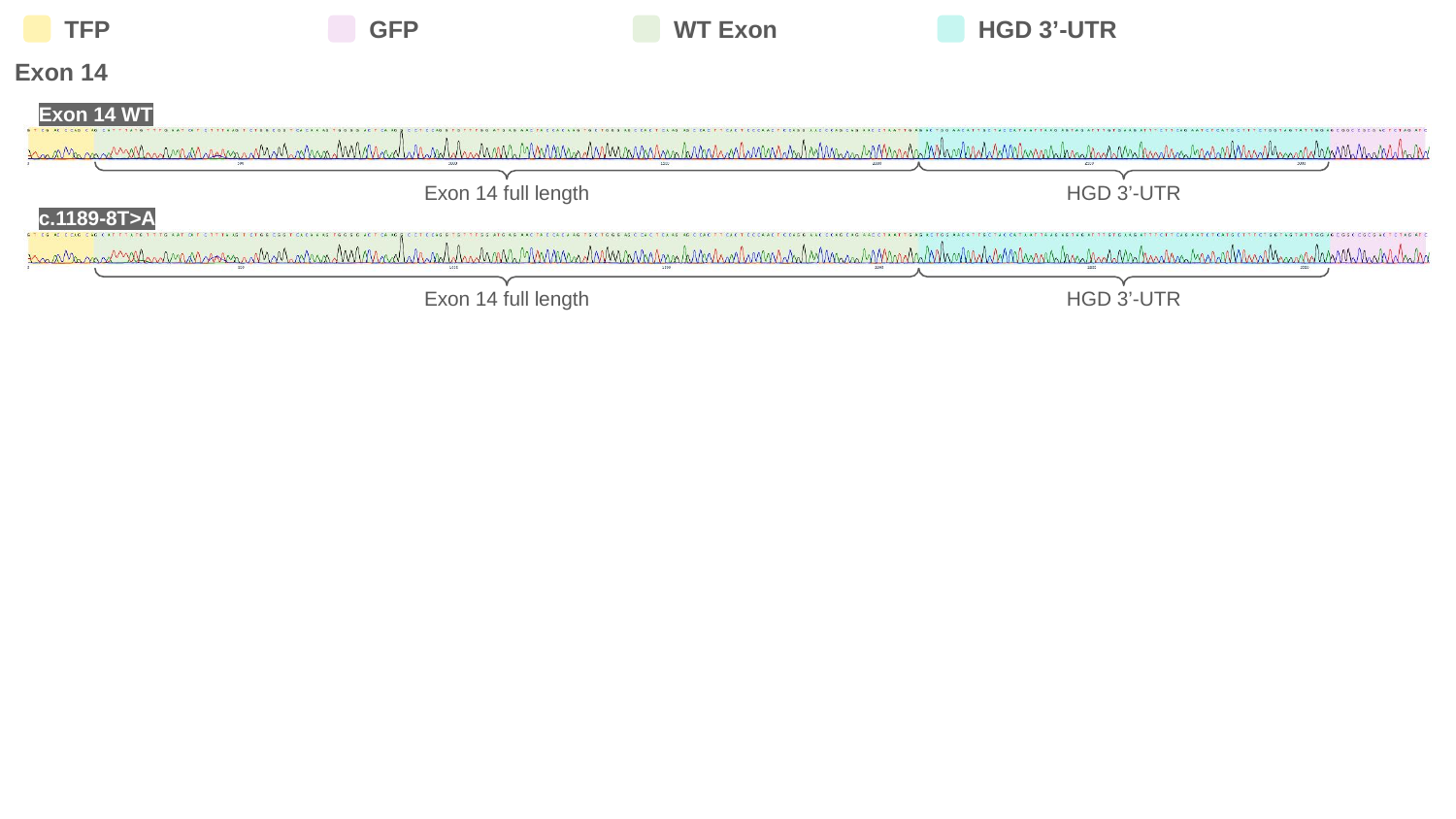

TFP
GFP
WT Exon
HGD 3’-UTR
Exon 14
Exon 14 WT
Exon 14 full length
HGD 3’-UTR
c.1189-8T>A
Exon 14 full length
HGD 3’-UTR
