# Supplementary material for: Functional Characterization of HGD Gene Variants by Minigene Splicing Assay
Source: Int J Mol Sci. 2025 Oct 31;26(21):10639. doi: 10.3390/ijms262110639 (PMC12608343; doi:10.3390/ijms262110639)
Supplement: Supplementary file 1 [file ijms-26-10639-s001.zip › Supplementary/Supplementary S5 (Regulation of exons 9 and 13).pptx]

## Slide 1
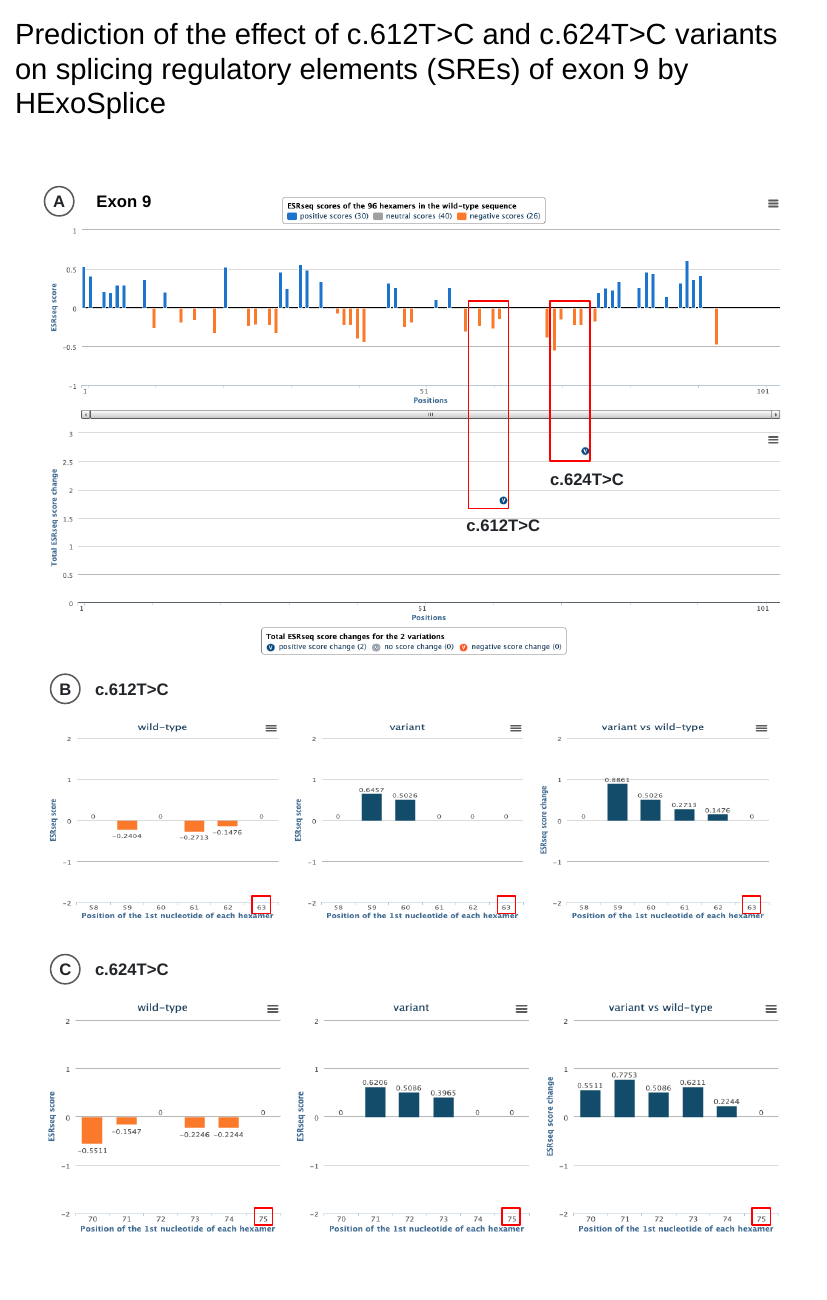

Prediction of the effect of c.612T>C and c.624T>C variants on splicing regulatory elements (SREs) of exon 9 by HExoSplice
А
Exon 9
c.624T>C
c.612T>C
c.612T>C
B
c.624T>C
C

## Slide 2
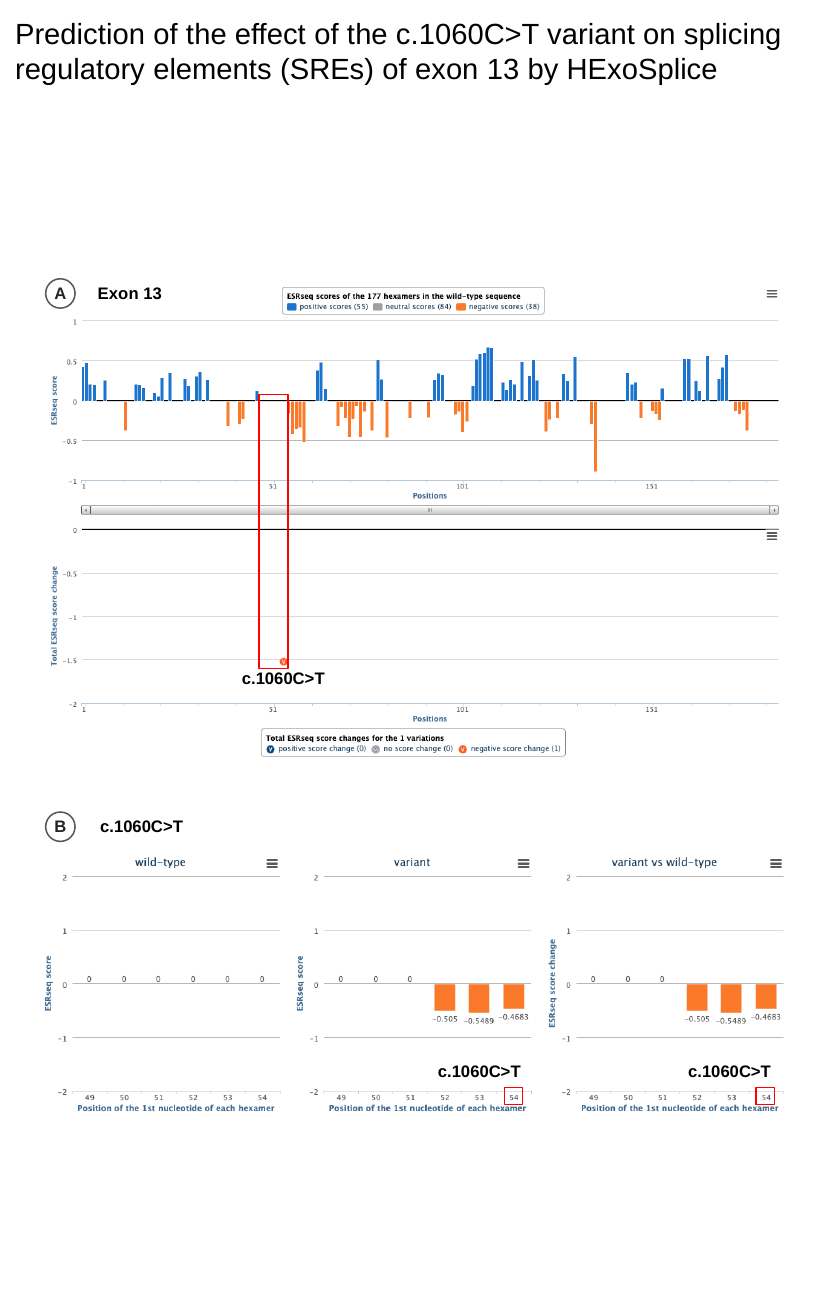

Prediction of the effect of the c.1060C>T variant on splicing regulatory elements (SREs) of exon 13 by HExoSplice
А
Exon 13
c.1060C>T
B
c.1060C>T
c.1060C>T
c.1060C>T
