# Supplementary figures and images for: Functional Characterization of HGD Gene Variants by Minigene Splicing Assay
Source: Int J Mol Sci. 2025 Oct 31;26(21):10639. doi: 10.3390/ijms262110639 (PMC12608343; doi:10.3390/ijms262110639)

Exons 1, 2, 4

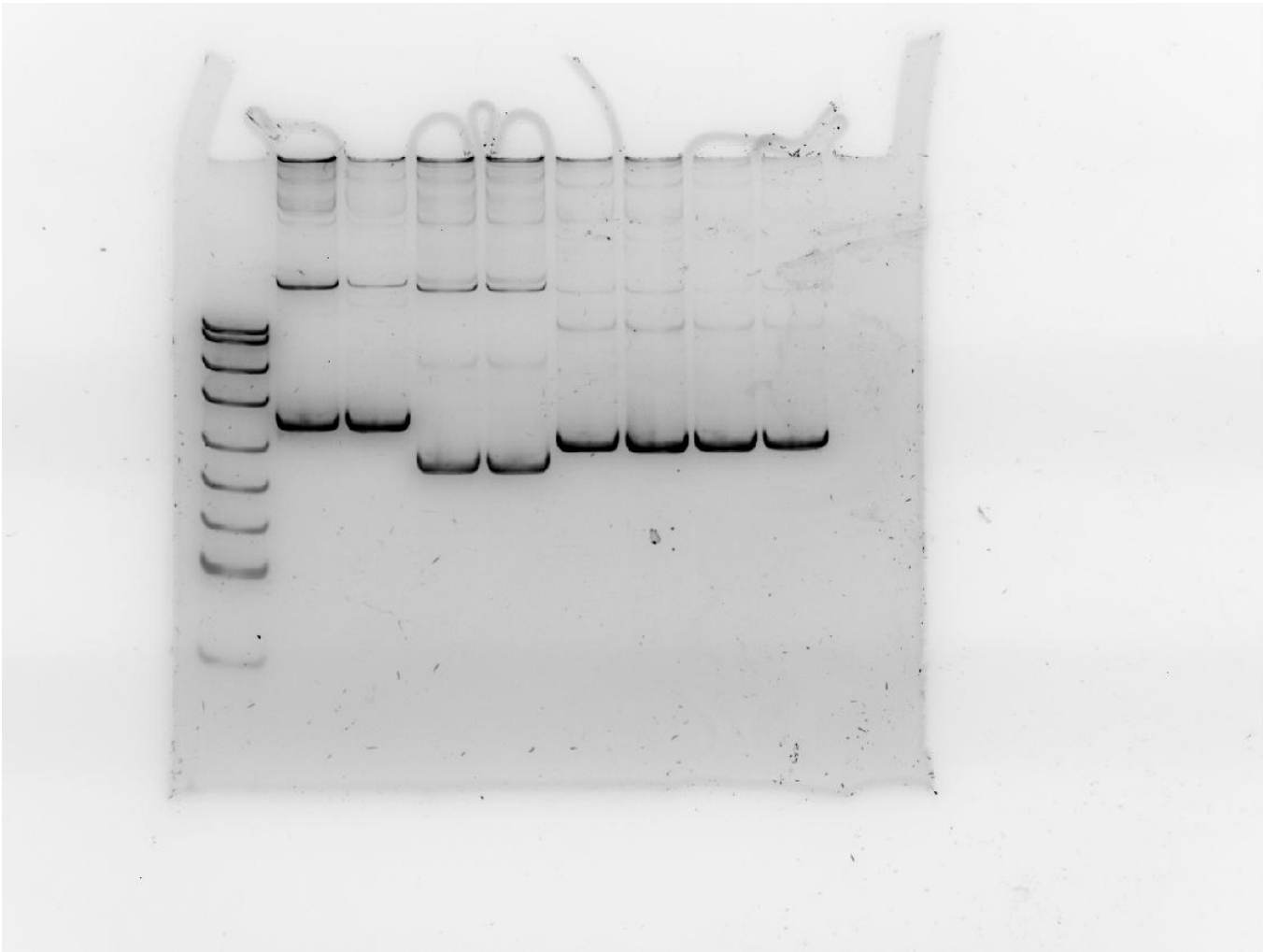

# Exon 3

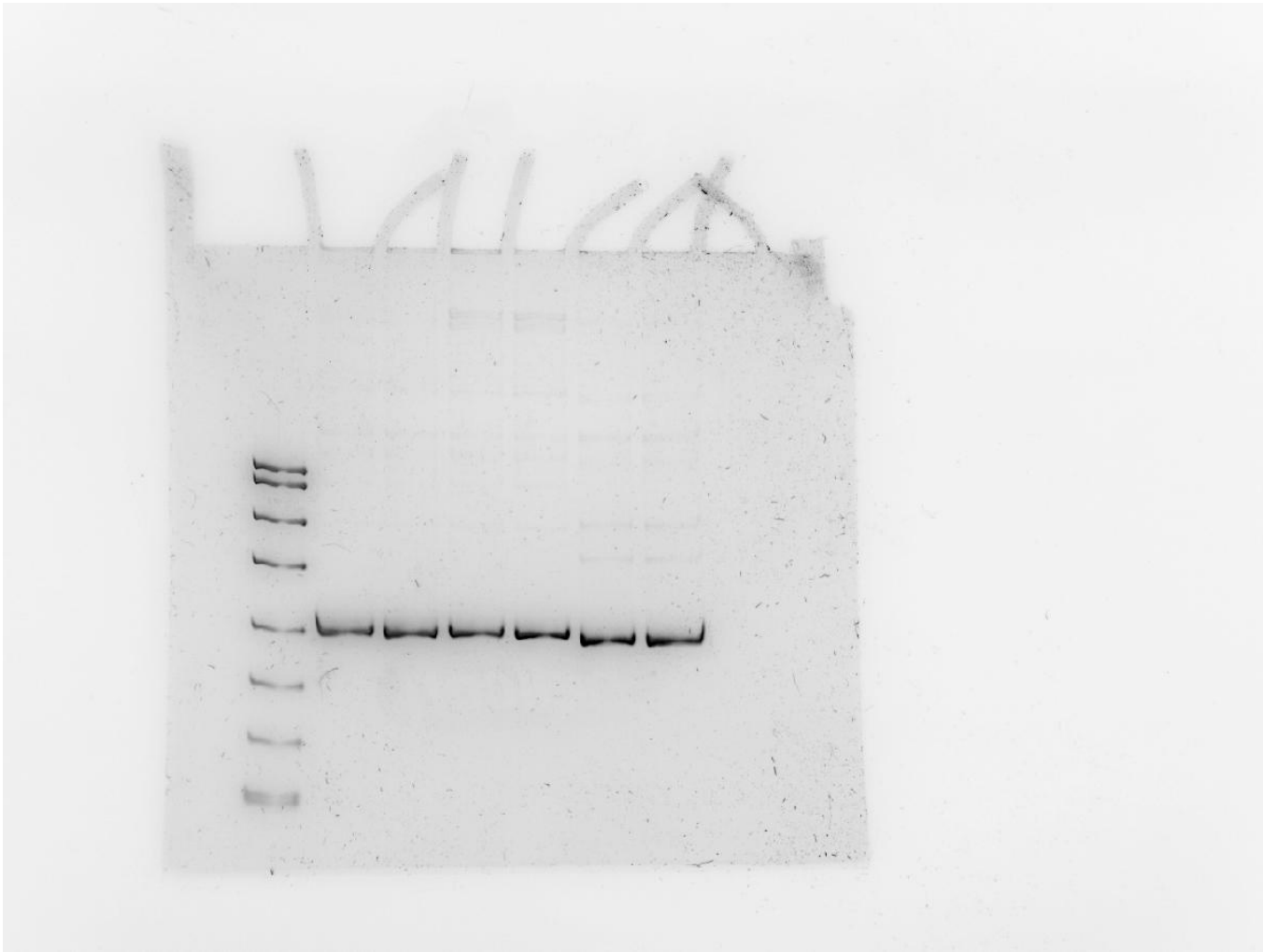

# Exon 5

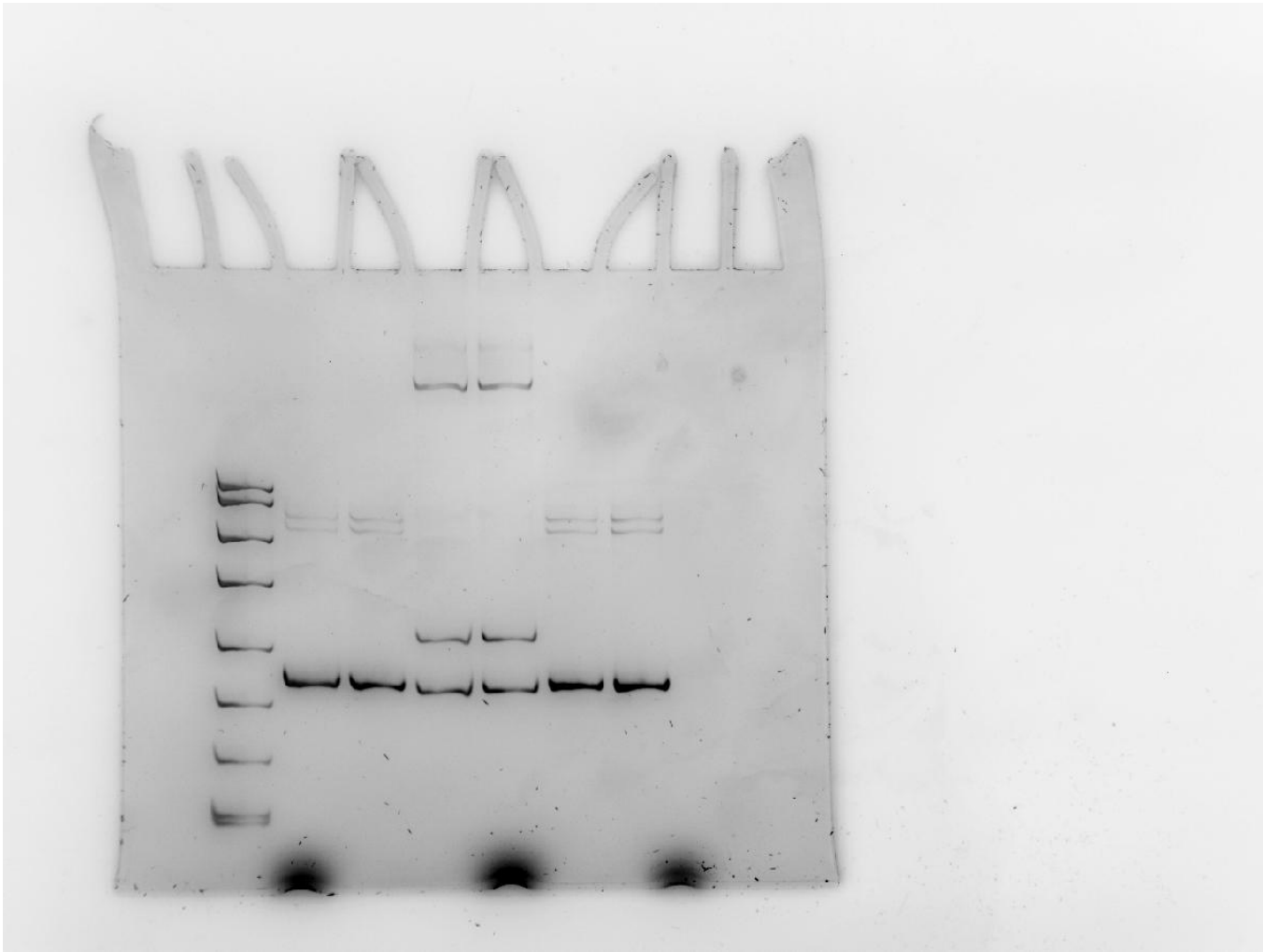

# Exon 6

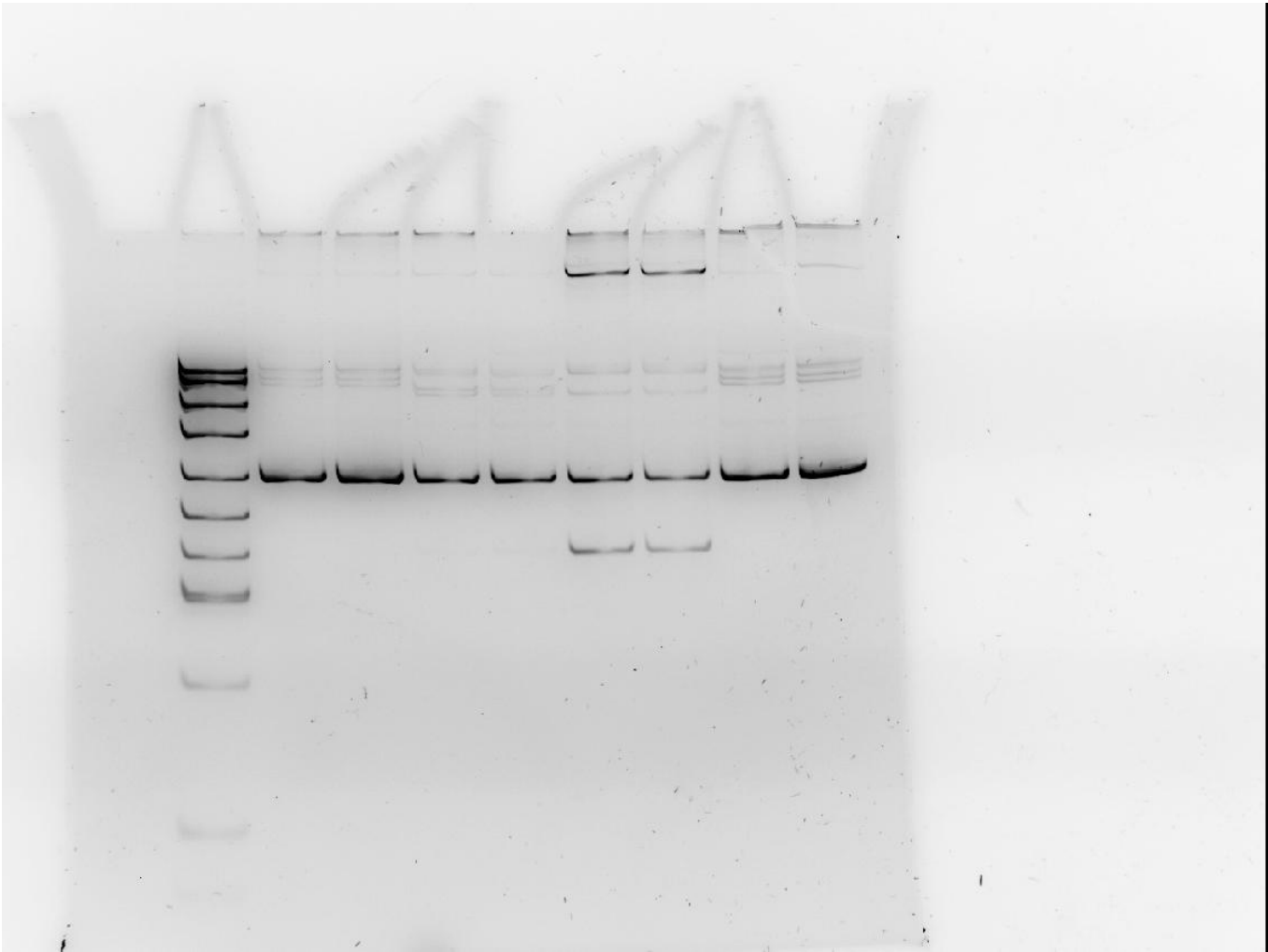

# Exon 7

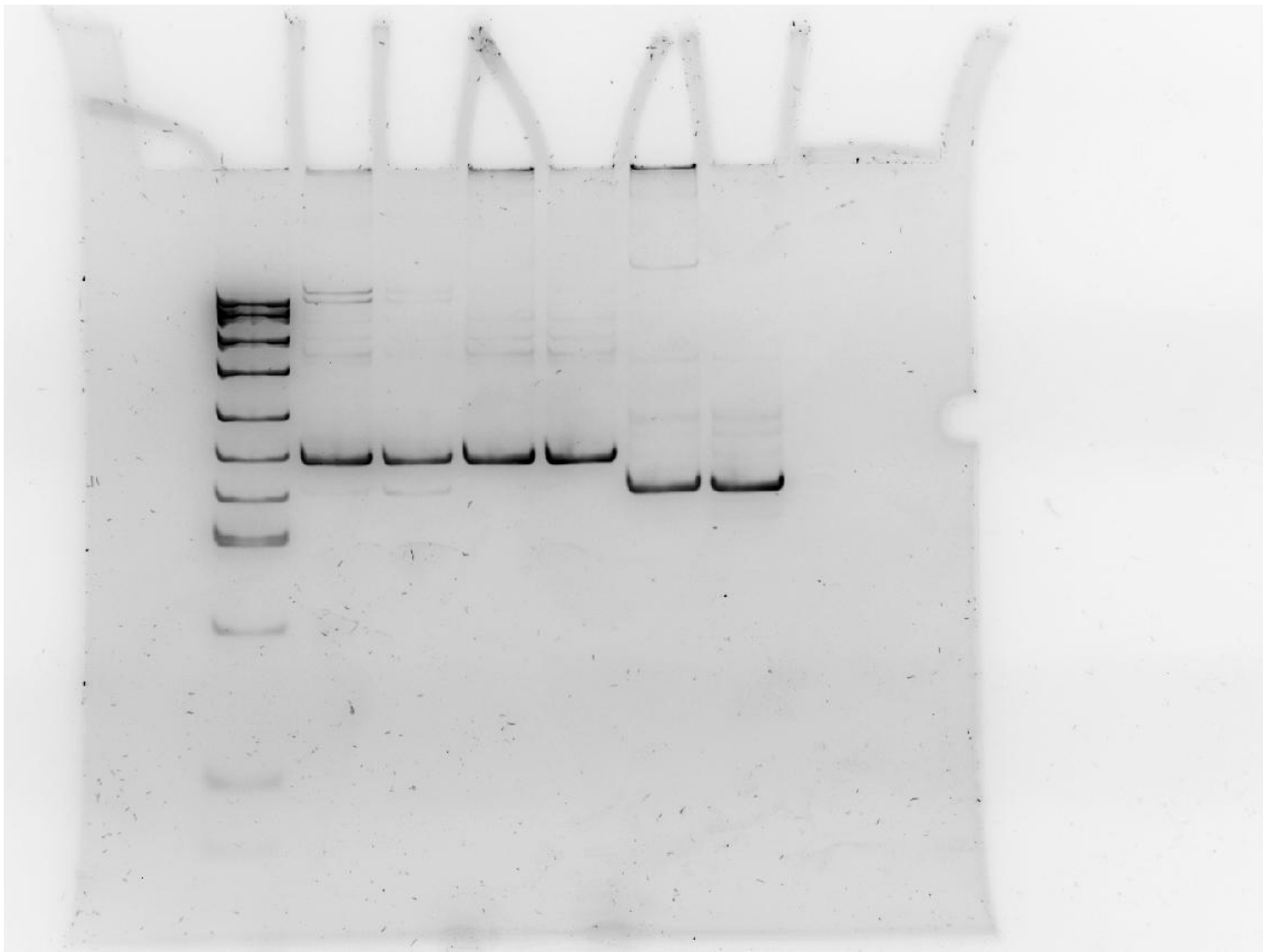

# Exon 7

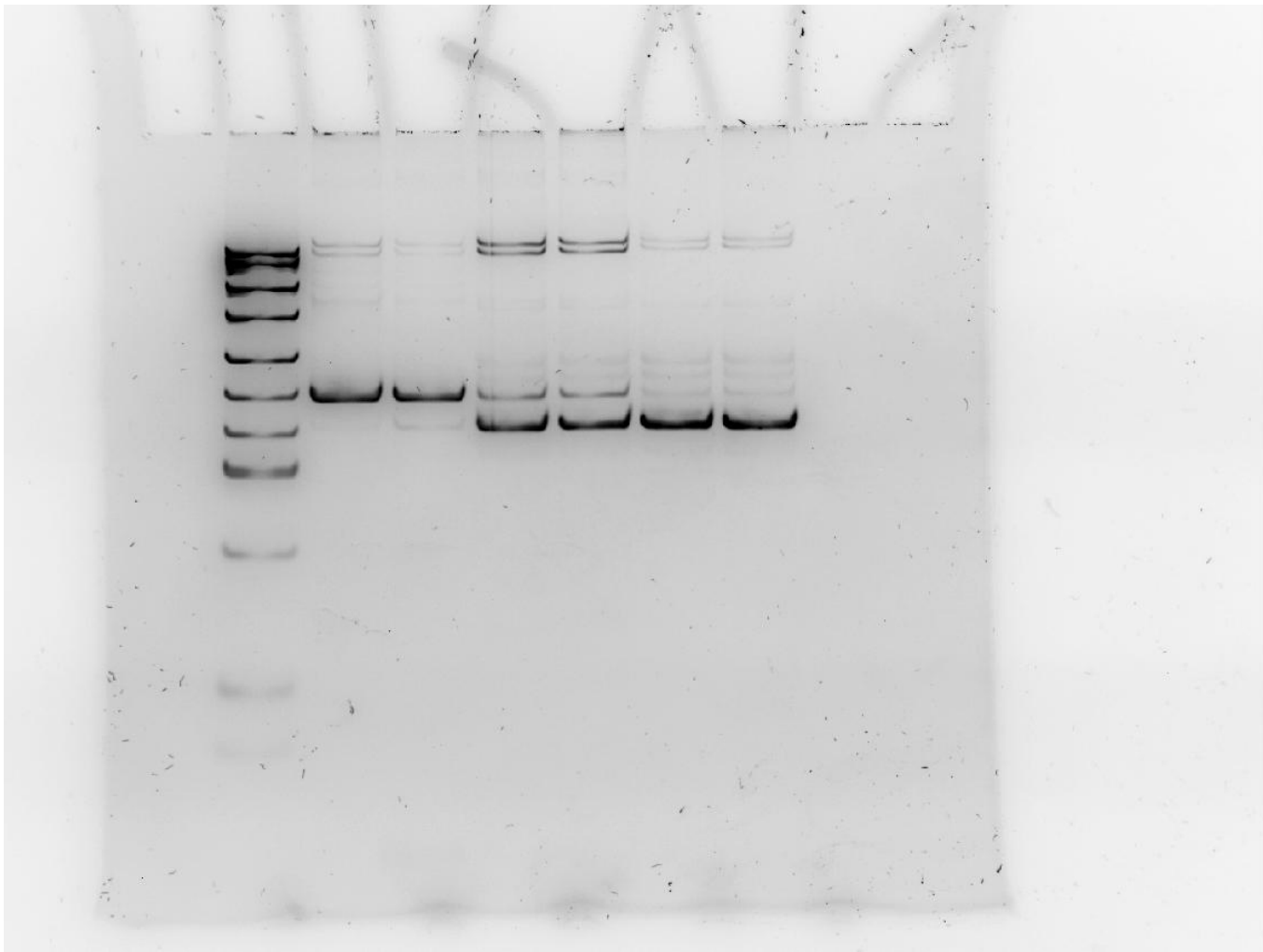

# Exons 8, 11

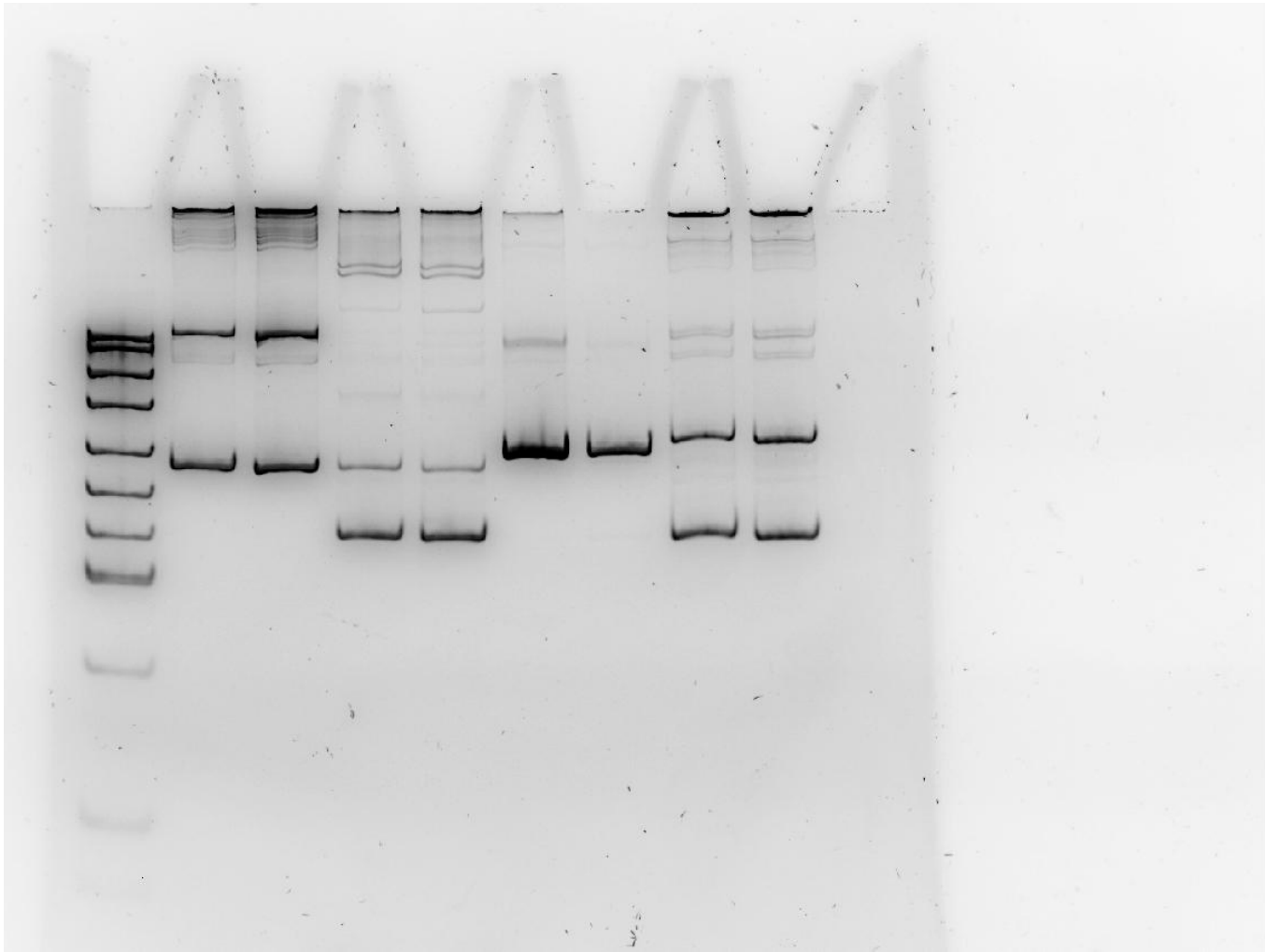

## Exon 9

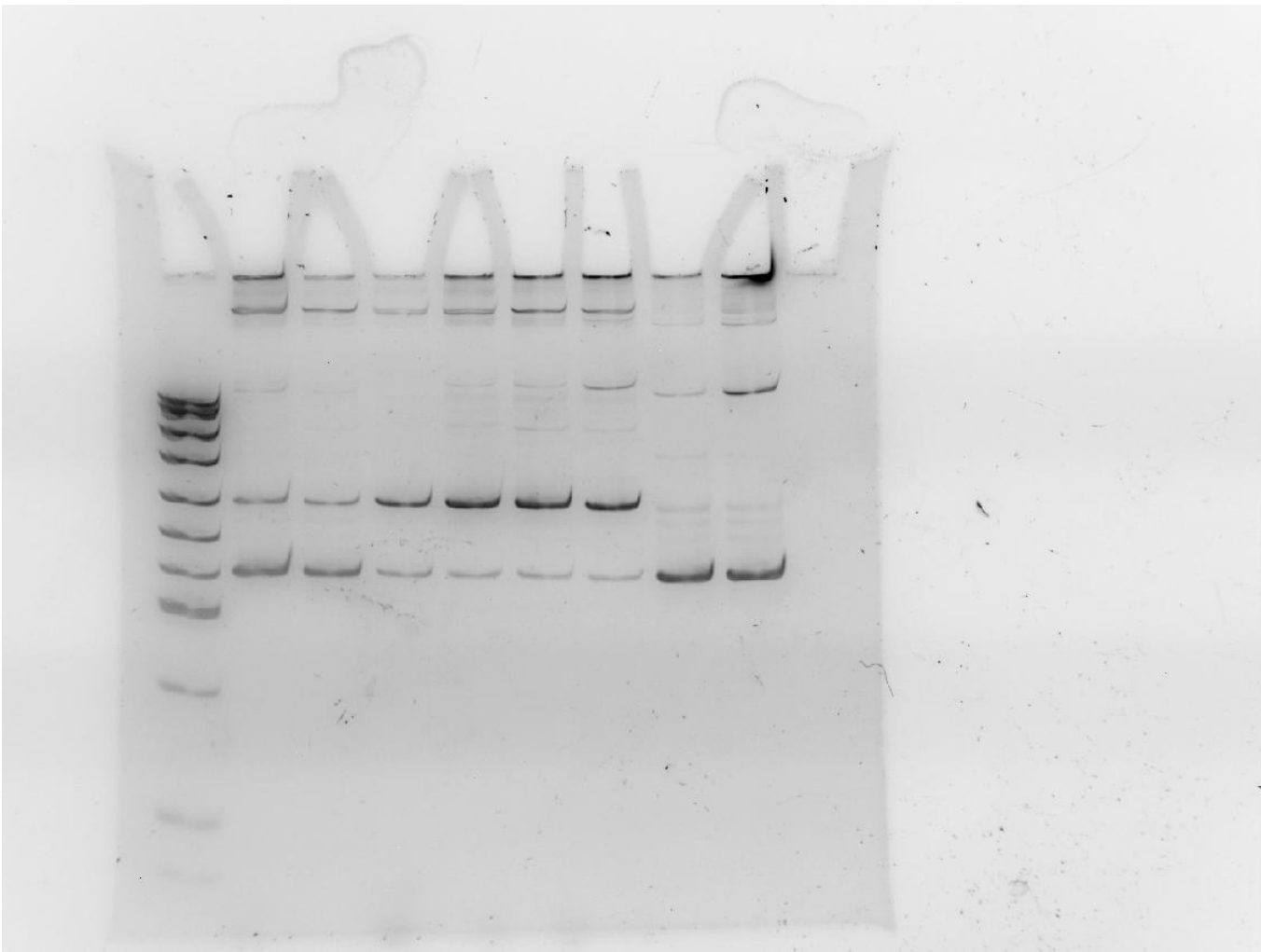

# Exon 10

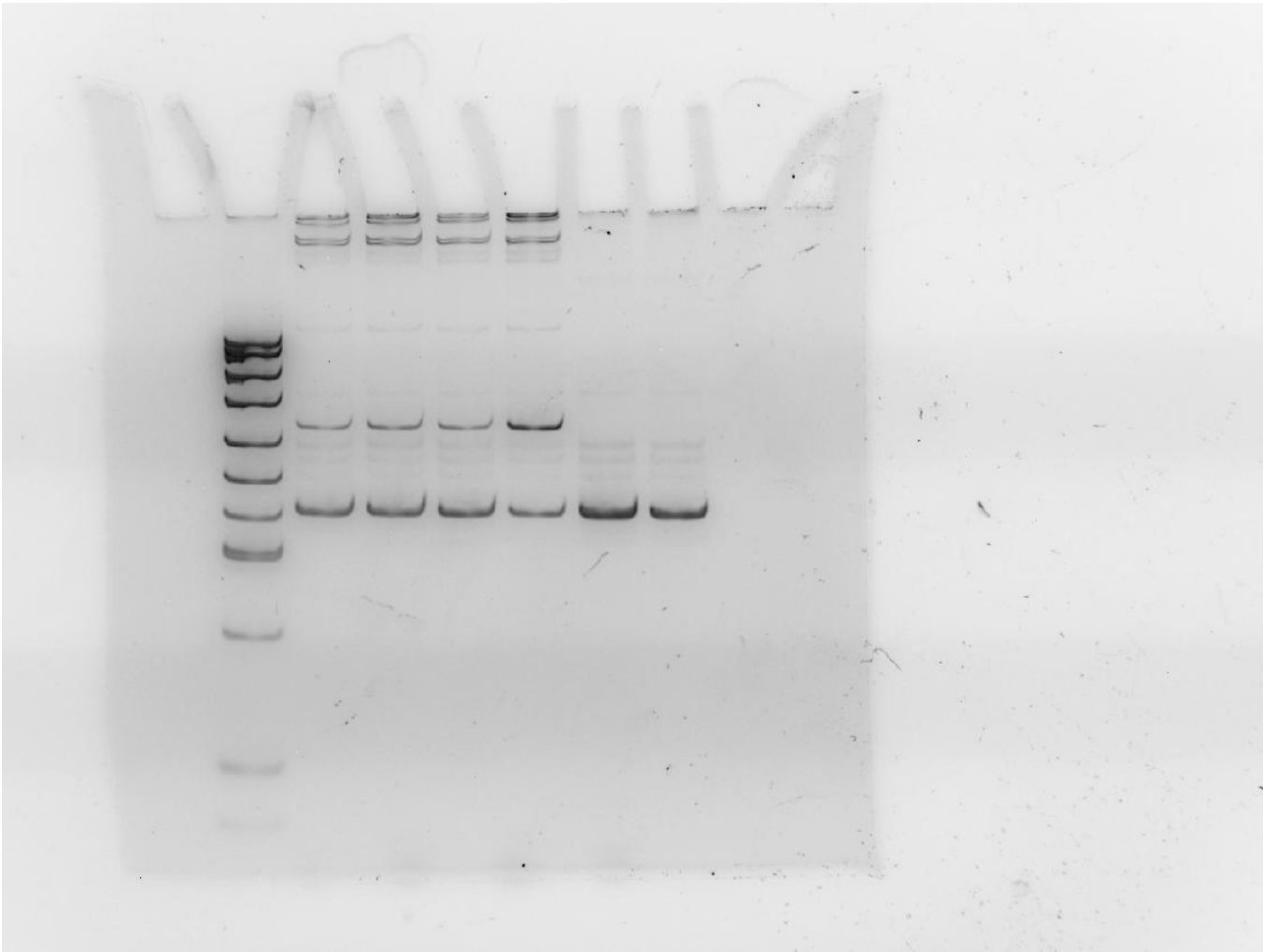

# Exon 12

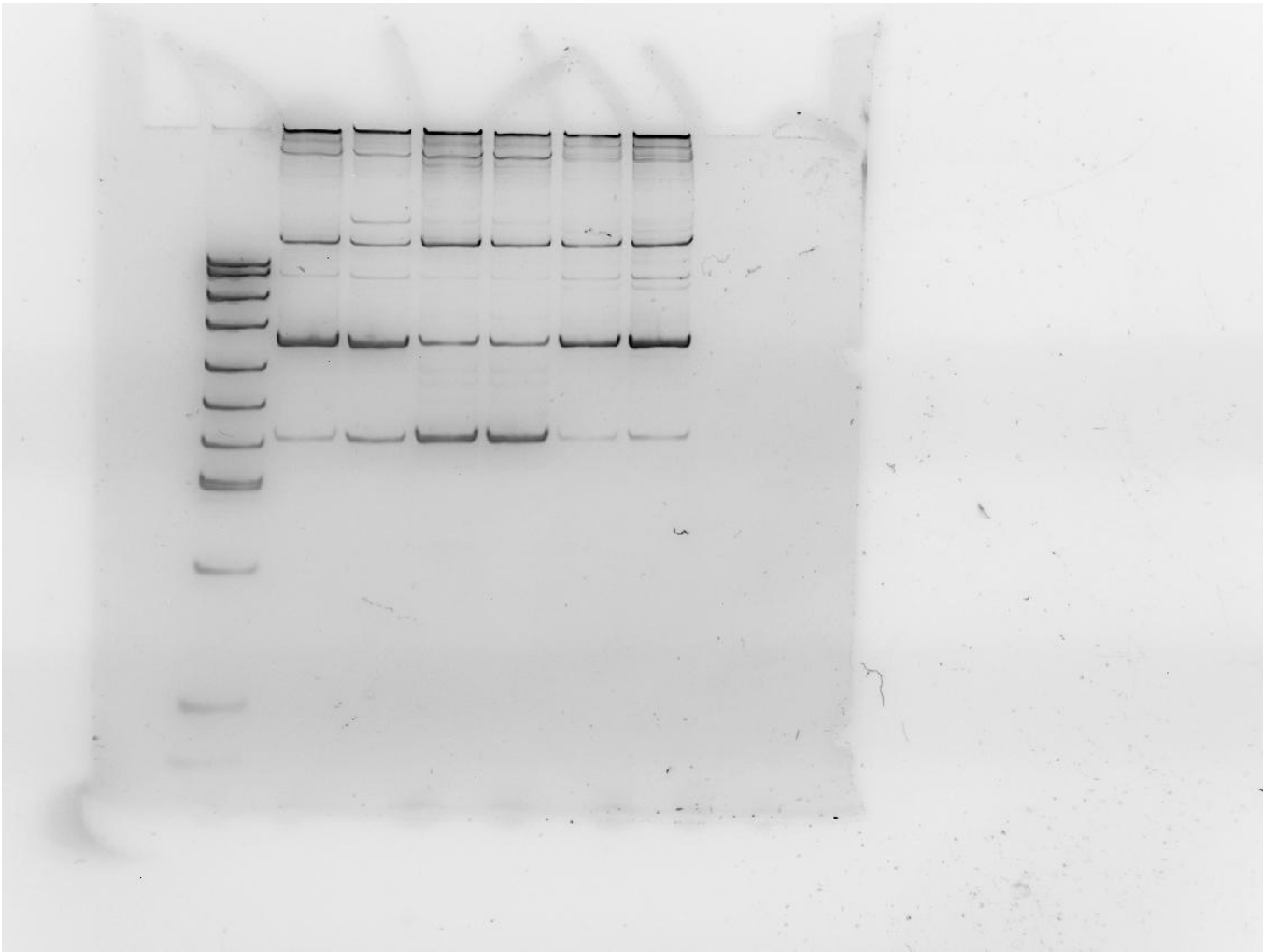

# Exon 13

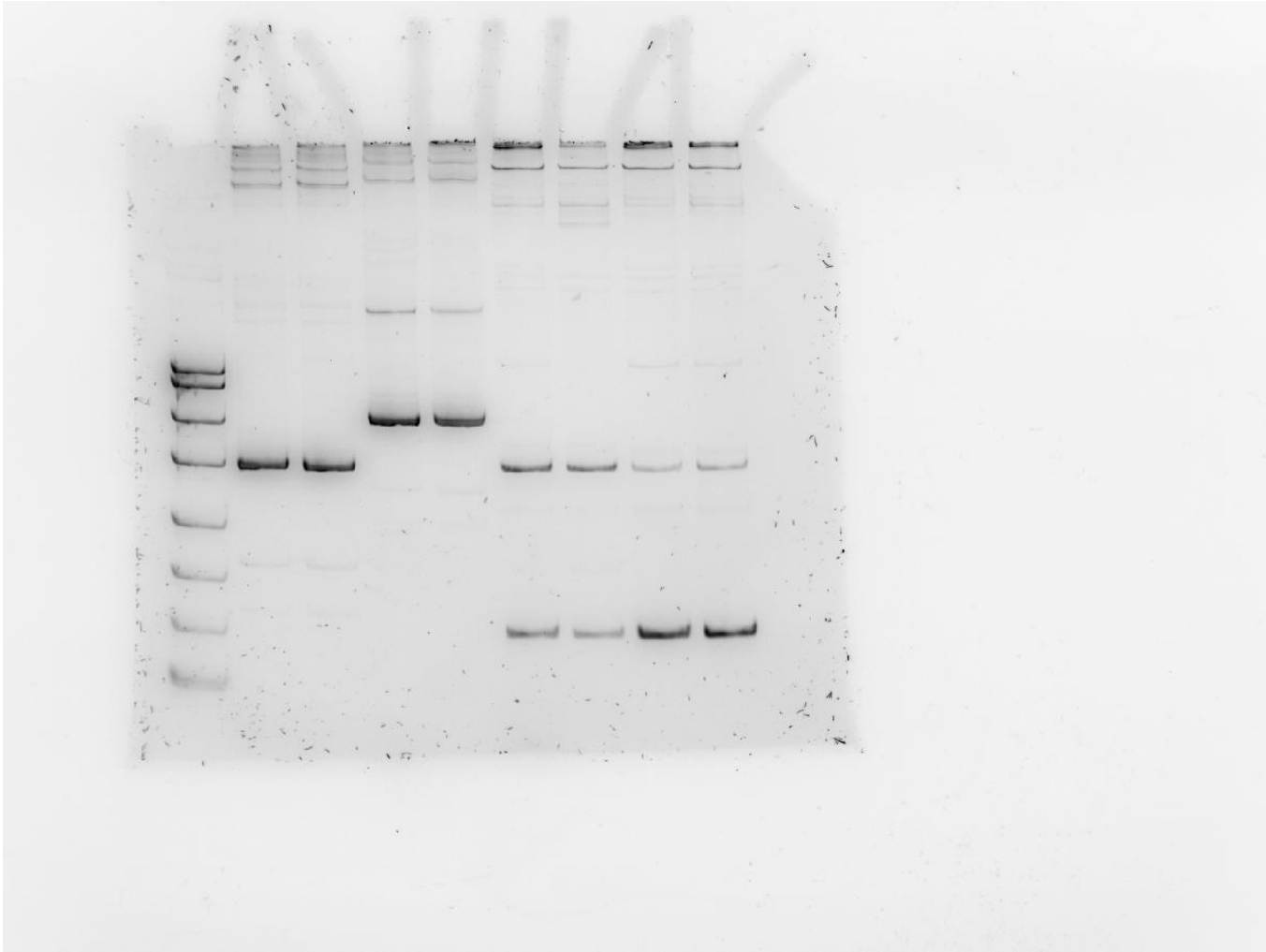

# Exon 13

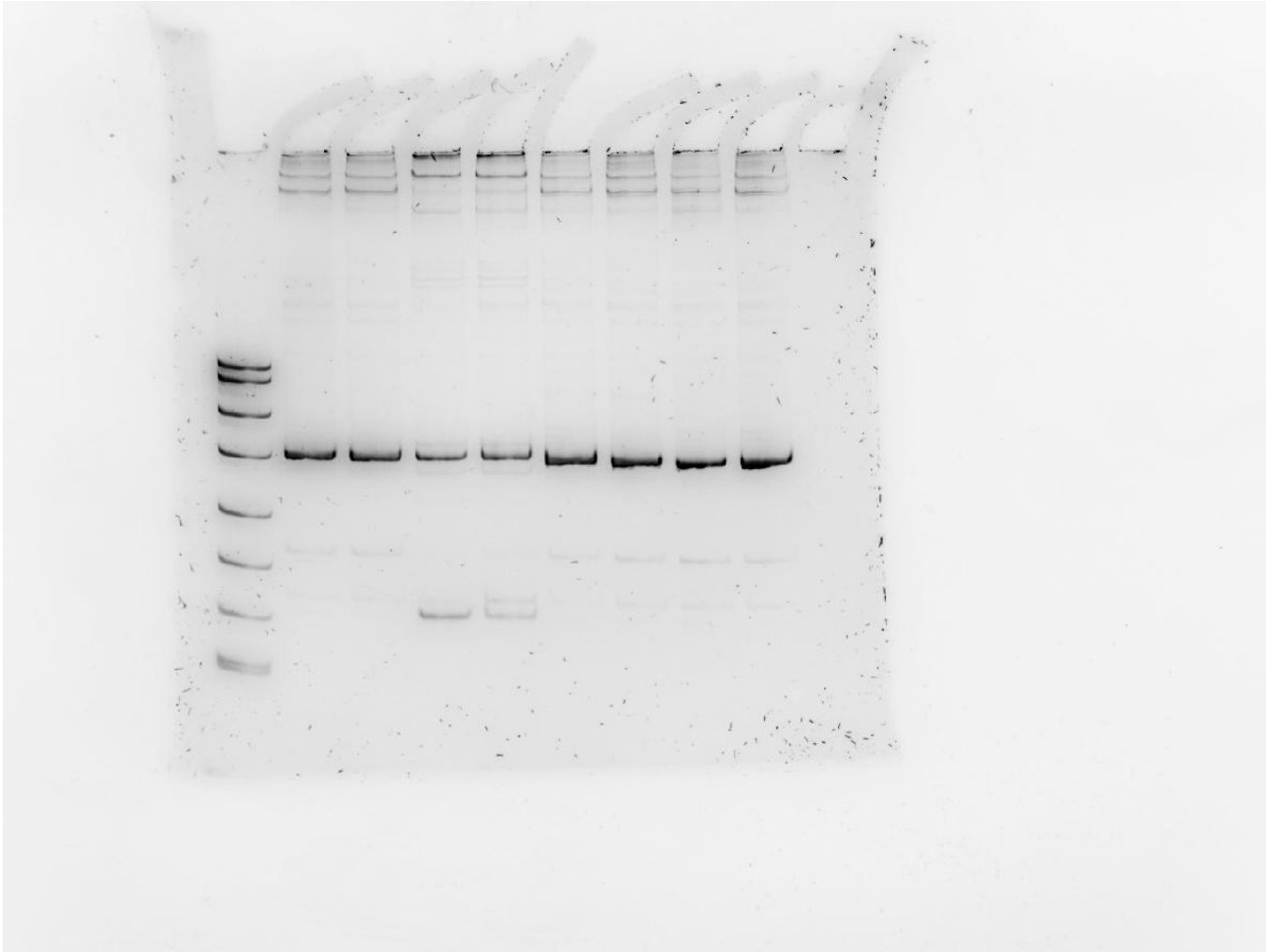

# Exon 14

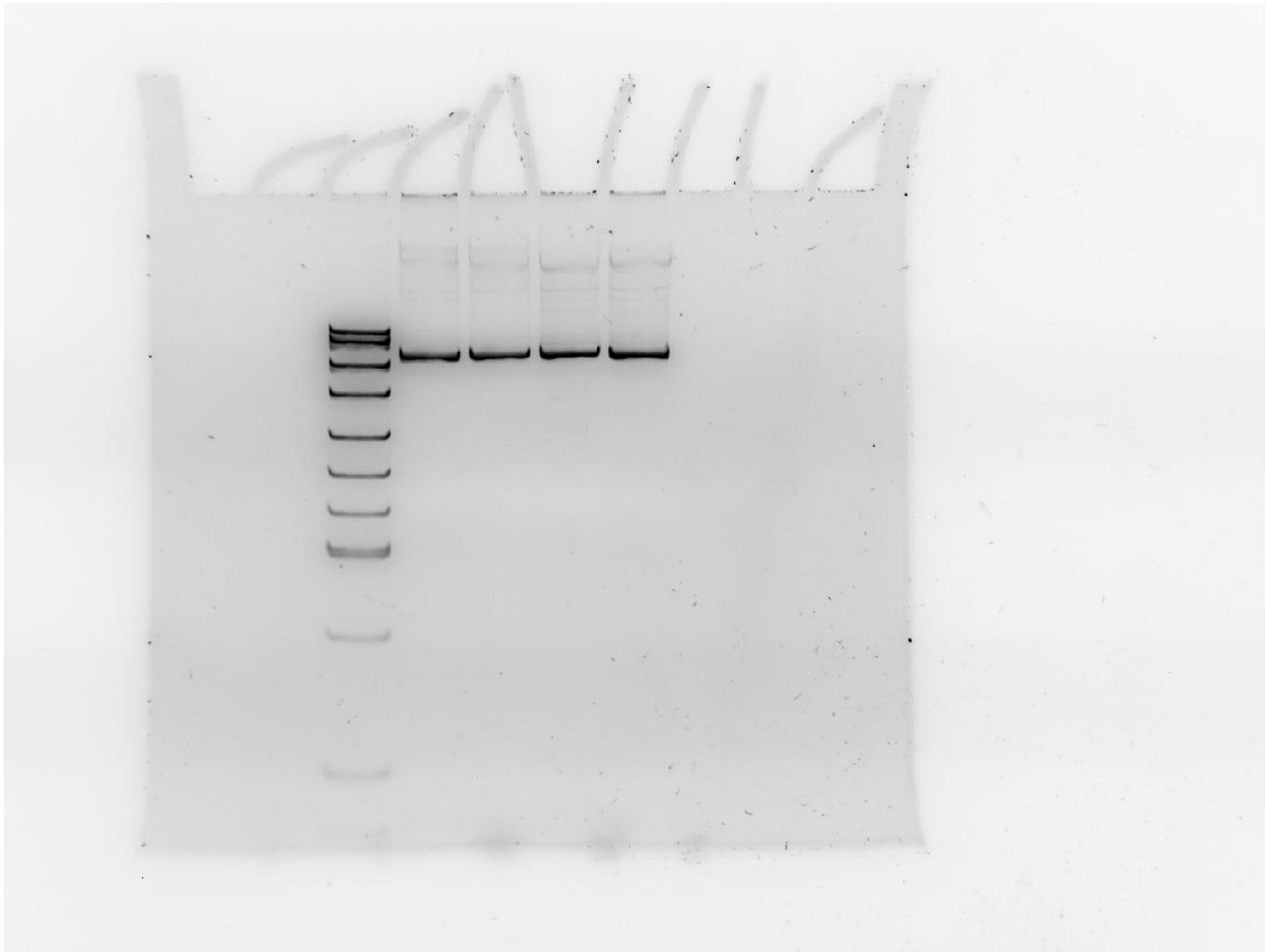

Supplement: Supplementary file 1 [file ijms-26-10639-s001.zip › Supplementary/PAAG.pdf]

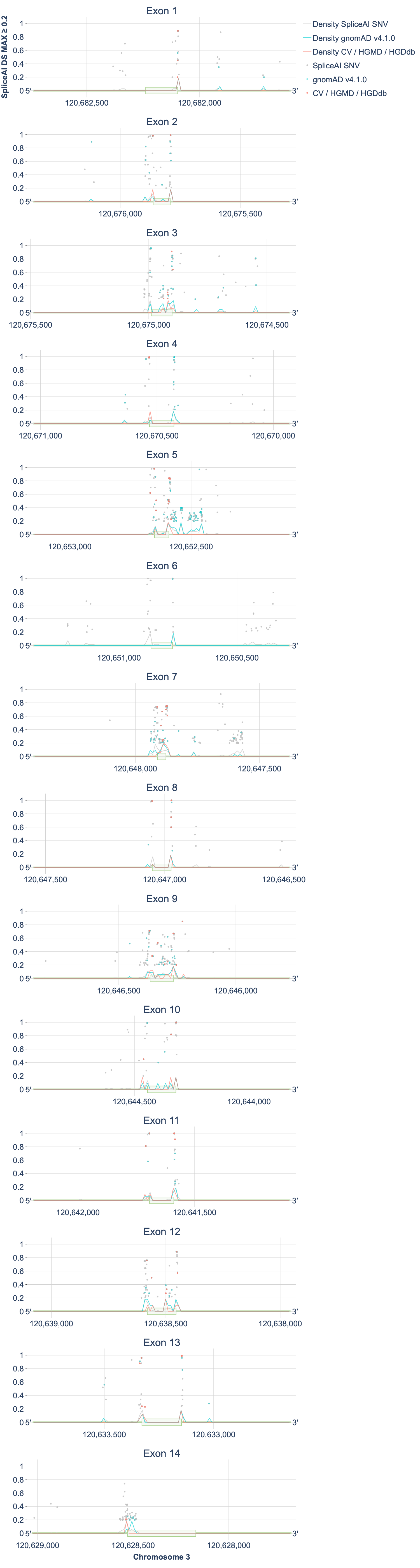

Supplement: Supplementary file 1 [file ijms-26-10639-s001.zip › Supplementary/Supplementary S6 (500).png]
